# Cis-acting lnc-eRNA SEELA directly binds histone H4 to promote histone recognition and leukemia progression

**DOI:** 10.1186/s13059-020-02186-x

**Published:** 2020-11-03

**Authors:** Ke Fang, Wei Huang, Yu-Meng Sun, Tian-Qi Chen, Zhan-Cheng Zeng, Qian-Qian Yang, Qi Pan, Cai Han, Lin-Yu Sun, Xue-Qun Luo, Wen-Tao Wang, Yue-Qin Chen

**Affiliations:** 1 MOE Key Laboratory of Gene Function and Regulation, State Key Laboratory for Biocontrol, School of Life Sciences, Sun Yat-sen University, Guangzhou, 510275 China; 2 The First Affiliated Hospital, Sun Yat-sen University, Guangzhou, 510080 China

**Keywords:** Lnc-eRNA, SEELA, Histone H4, Histone recognition, Enhancer activity, Sphingolipid metabolism, *MLL* leukemia

## Abstract

**Background:**

Long noncoding enhancer RNAs (lnc-eRNAs) are a subset of stable eRNAs identified from annotated lncRNAs. They might act as enhancer activity-related therapeutic targets in cancer. However, the underlying mechanism of epigenetic activation and their function in cancer initiation and progression remain largely unknown.

**Results:**

We identify a set of lncRNAs as lnc-eRNAs according to the epigenetic signatures of enhancers. We show that these lnc-eRNAs are broadly activated in *MLL*-rearranged leukemia (*MLL* leukemia), an aggressive leukemia caused by a chromosomal translocation, through a mechanism by which the HOXA cluster initiates enhancer activity, and the epigenetic reader BRD4 cooperates with the coregulator MLL fusion oncoprotein to induce transcriptional activation. To demonstrate the functional roles of lnc-eRNAs, two newly identified lnc-eRNAs transcribed from the SEELA eRNA cluster (SEELA), SEELA1 and SEELA2, are chosen for further studies. The results show that SEELA mediated cis-activated transcription of the nearby oncogene *Serine incorporate 2* (*SERINC2*) by directly binding to the K31 amino acid (aa) of histone H4. Chromatin-bound SEELA strengthens the interaction between chromatin and histone modifiers to promote histone recognition and oncogene transcription. Further studies show that the SEELA-SERINC2 axis regulated aspects of cancer metabolism, such as sphingolipid synthesis, to affect leukemia progression.

**Conclusions:**

This study shows that lnc-eRNAs are epigenetically activated by cancer-initiating oncoproteins and uncovers a cis-activating mechanism of oncogene transcription control based on lnc-eRNA-mediated epigenetic regulation of enhancer activity, providing insights into the critical roles of lnc-eRNAs in cancer initiation and progression.

**Supplementary information:**

**Supplementary information** accompanies this paper at 10.1186/s13059-020-02186-x.

## Background

Enhancers are essential DNA elements that positively modulate the transcription of target genes [1, 2] and are transcribed to noncoding RNAs as eRNAs [3, 4]. Recently, a novel class of eRNAs referred to as lnc-eRNAs has been identified [5]. Lnc-eRNAs are annotated lncRNAs in which initiation sites overlap with enhancer regions. Compared to other types of eRNAs, lnc-eRNAs are acknowledged to be more stable [5]. Importantly, the genomic loci of lnc-eRNAs overlap with those of tissue-specific enhancers [6], and lnc-eRNA expression is tightly correlated with the epigenetic signatures of enhancers that are controlled by histone modifiers [7]. Dysregulation of enhancer activity, including abnormal expression of eRNAs, is frequently observed in cancer [8, 9], and targeting enhancer activity-related histone modifiers has shown promise in cancer treatment [10]. Notably, extensive genome-wide studies have revealed that lncRNAs are dysregulated in almost all types of cancer [11, 12]; however, whether these lncRNAs act as lnc-eRNAs to regulate cancer initiation and progression remains largely unknown.

*Cis* regulation is a general regulatory mechanism for eRNAs in cancer [13–15]. In *cis* regulation, eRNAs are preferentially localized to their transcription sites and modulate enhancer activity at the chromatin level [16] via mechanisms including chromatin remodeling [17], RNA polymerase II (POL II) pause release [18], and recruitment of general regulators [19–21], suggesting that the proper localization of lnc-eRNAs on chromatin is important for their function. Thus, exploring the chromatin retention mechanism will improve our understanding of lnc-eRNA regulation.

*MLL*-rearranged leukemia, caused by Mixed Lineage Leukemia (*MLL*) gene translocation-induced expression of the MLL fusion protein, has the worst prognosis and is the most aggressive among leukemia subtypes [22]. The MLL fusion oncoprotein transcriptionally activates oncogenes to induce hematologic malignancy by interacting with BRD4 [23], which is a member of the bromodomain and extraterminal motif (BET) protein family and an epigenetic reader. Notably, *HOXA9*, one of the most important genes downstream of the MLL fusion oncoprotein [24], is reported to rewire gene regulatory networks by mediating the establishment of leukemia-specific enhancers [25], and the MLL fusion oncoprotein itself has the ability for enhancer binding [26]. These observations suggest that enhancer activity may play a pivotal role in *MLL* leukemia progression. However, whether MLL fusion oncoproteins activate lnc-eRNAs and the mechanism underlying how these lnc-eRNAs affect disease progression warrants demonstration.

In this study, we identified a set of lncRNAs acting as lnc-eRNAs that are activated in *MLL* leukemia through a HOXA initiation-BRD4 transcriptional activation mechanism, by which the MLL fusion downstream HOXA cluster initiates enhancer establishment, and the epigenetic reader BRD4 cooperates with the coregulator MLL fusion oncoprotein to induce enhancer transcription. We further showed that the newly identified lnc-eRNA, SEELA, directly interacted histone H4 to localize on chromatin and strengthened the binding between the histone modifier and chromatin to promote histone recognition and enhancer activity. As a result, SEELA activated its nearby oncogene *SERINC2* and influenced disease progression by affecting sphingolipid metabolism. Collectively, our results revealed that the MLL fusion oncoprotein-driven SEELA directly binds histone H4 to promote histone recognition and enhancer activity in cancer.

## Results

### Identification of a set of lncRNAs acting as lnc-eRNAs according to the epigenetic signatures of enhancers

**Fig. 1:**
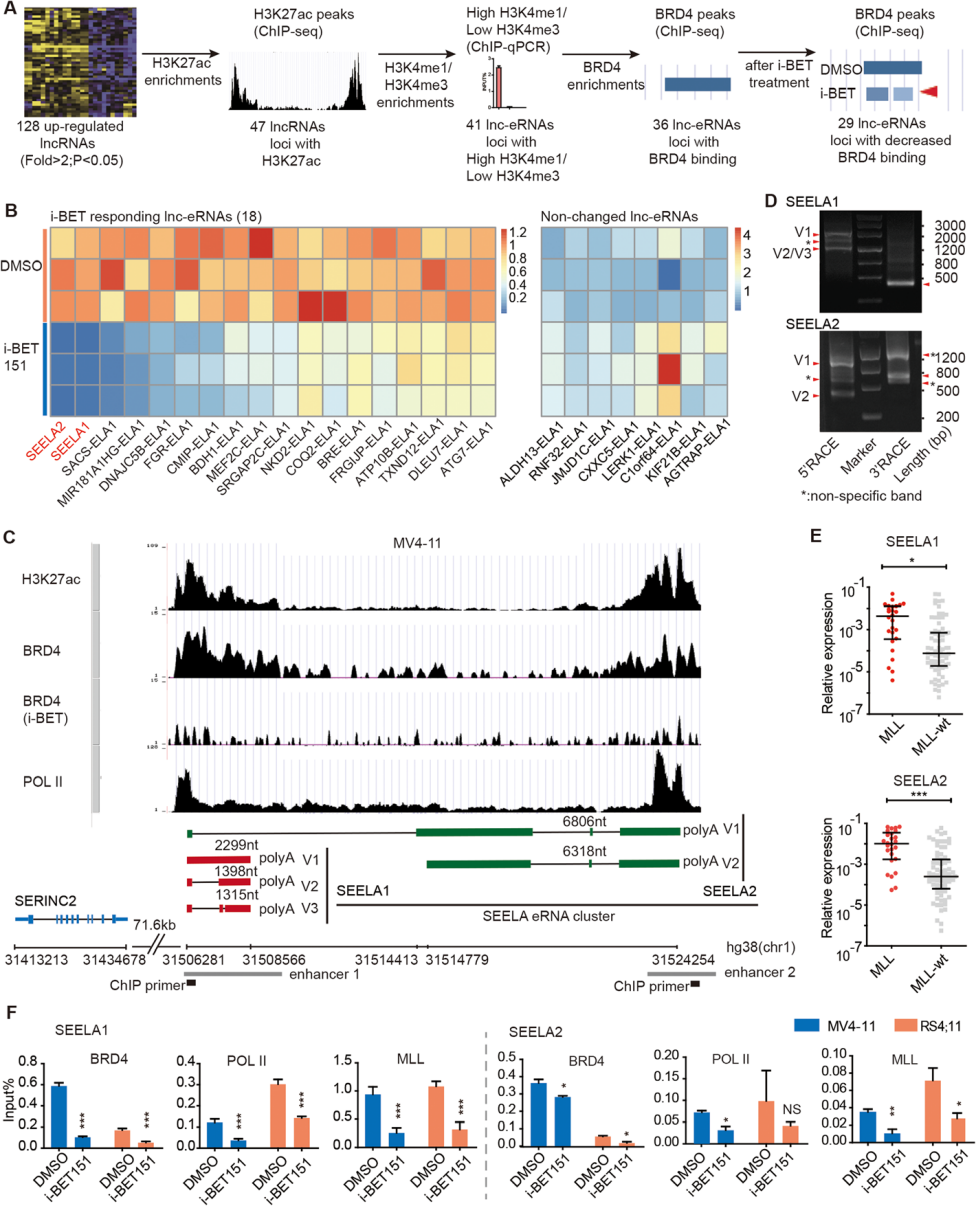
A set of lncRNAs are identified as lnc-eRNAs and are activated through abnormal enhancer transcription. **a** Analysis of the gene loci of 128 upregulated lncRNAs with H3K27ac, H3K4me1, H3K4me3 and BRD4 enrichment. **b** Heatmaps of the qPCR assay results showed the expression levels of lnc-eRNAs in MV4-11 cells treated with i-BET151. Eighteen lnc-eRNAs were downregulated, 8 lnc-eRNAs were unchanged, and 3 lnc-eRNAs were not detected in three independent experiments. **c** The epigenetic environment of *SEELA* and its genomic relationship with *SERINC2*. *SEELA* is located 71.6 kb downstream of *SERINC2*, and its gene locus is marked by H3K27ac, BRD4 and POL II in two enhancer regions (as shown in schematic diagrams (gray)). The ChIP-qPCR primers were designed to target the gene body of *SEELA* (black). **d** DNA agarose gels showing that there were three and two variants of SEELA1 and SEELA2, respectively, according to the results of 5′ and 3′ RACE assays. *Nonspecific band. **e** The expression of SEELA was significantly upregulated in patients with *MLL* leukemia (*n* = 26) compared with *MLL*-wt (*n* = 75) patients (*MLL*-wt, leukemia patients not harboring *MLL *gene translocation). (Mann-Whitney test; *P < 0.05; ***p < 0.001). The expression level was calculated using the 2^-ΔCT^ method and was normalized to that of GAPDH. **f** ChIP-qPCR detection of BRD4, POL II, and MLL fusion protein enrichment at the *SEELA1* and *SEELA2* gene loci in MV4-11 and RS4;11 cells after treatment with i-BET151. The error bars indicate the ± SEM values (*P < 0.05; **P < 0.01; ***P < 0.001; ns, no significant difference) in three independent experiments

Abnormal expression of lncRNAs has been reported in multiple cancer types [27]; however, whether these lncRNAs act as lnc-eRNAs remains unclear. To identify lncRNAs that are transcribed from enhancers as lnc-eRNAs in *MLL* leukemia, we analyzed the lncRNA expression profile and enhancer epigenetic profile. Starting from a verified set of 128 upregulated lncRNAs from our previous microarray study [28], we first identified that the gene loci of 47 lncRNAs were marked by H3K27ac (Additional file 1: Table S1), a histone mark of active enhancers (as assessed by ChIP-seq data from the *MLL* leukemia cell line MV4-11), indicating that these lncRNAs might act as lnc-eRNAs. In addition, two other key histone marks (H3K4me1 and H3K4me3) [5] of enhancers were also measured by ChIP-qPCR. The results showed that 41 of 47 candidate lnc-eRNA loci showed high enrichment of H3K4me1 and relatively low enrichment of H3K4me3 (Fig. 1a and Additional file 2: Fig. S1), confirming that these lnc-eRNAs were transcribed from enhancer regions. These lnc-eRNAs were named GENE-Enhancer-LncRNAs (ELAs) according to their predicted target genes. The prediction of lnc-eRNAs target genes was based on previous report [6] and the genomic distance. Briefly, the nearest gene located within 100 kb of lnc-eRNA locus is considered as its target gene. We then selected 36 lnc-eRNAs according to the enrichment level of the epigenetic reader BRD4 of H3K27ac-enriched enhancer and obtained 29 lnc-eRNAs candidates using sensitivity to the BRD4 inhibitor i-BET151 treatment as a filter (Fig. 1a and Additional file 1: Table S1). Further qRT-PCR validation showed that the expression levels of 18 of these 29 lnc-eRNAs were decreased after i-BET151 treatment (Fig. 1b), indicating that a large proportion of upregulated lncRNAs could act as lnc-eRNAs and were transcribed through BRD4 regulation. We classified these 18 lnc-eRNAs as a group of “iBET-responsive lnc-eRNAs” and speculated that they might be involved in *MLL* leukemia progression.

### Lnc-eRNA SEELA is activated through oncoprotein-driven transcription

To further explore the potential roles of lnc-eRNAs, we selected two lnc-eRNAs, SERINC2-ELA1 and SERINC2-ELA2 (named SEELA1 and SEELA2, respectively), that displayed the greatest decrease in expression after i-BET151 treatment (Fig. 1b) for further study. *SEELA1* and *SEELA2*, annotated as *LDC1P* and *LINC01226*, respectively, in the human reference genome GRCh38/hg38, are located on chromosome 1p35.2. ChIP-seq data of the large-scale (including *SEELA1/2*, *SERINC2*, and two other nearby genes *FABP3* and *TINAGL1*) and zoomed-in regions (only including *SEELA1/2* and *SERINC2*) showed specific enrichment of H3K27ac and BRD4 at the *SEELA1/2* locus and POL II enrichment at the *SERINC2* and *SEELA1/2* loci (Fig. 1c and Additional file 2: Fig. S2A). Furthermore, insertion of the H3K27ac-enriched DNA sequence of *SEELA1/2* into the PGL-3 SERINC2 promoter plasmid significantly upregulated luciferase activity (Additional file 2: Fig. S2B). These results are consistent with the idea that SEELA1/2 is transcribed from the active enhancer of the *SERINC2* gene [6]. Both SEELA1 and SEELA2 were 5′ m7G capped and 3′ polyadenylated (Additional file 2: Fig. S2C, D). RNA-seq tracks and RACE assays showed that SEELA1 had three variants (SEELA1-V1 to V3) and SEELA2 has two (SEELA2-V1 to V2) in MV4-11 cells, respectively (Fig. 1d and Additional file 2: Fig. S2E). Notably, SEELA2-V1 shared the same 5′ end with all variants of SEELA1, and there was no POL II or H3K27ac enrichment at the transcription start site of SEELA2-V2, suggesting that both SEELA1 and SEELA2 might be transcribed from the same enhancer (enhancer 1) and then processed into different isoforms. Thus, we called these lnc-eRNAs (including three SEELA1 variants and two SEELA2 variants) as the SEELA eRNA cluster (hereafter called SEELA). Notably, SEELA showed the same expression pattern across leukemia cell lines (Additional file 2: Fig. S2F) and we used the primers (Additional file 2: Fig. S2G) to examine the expression of all SEELA1 or SEELA2 variants in the following study. SEELA was upregulated in *MLL* leukemia patient samples (Fig. 1e). The MLL fusion oncoprotein has been reported to cooperate with the histone acetylation reader BRD4 to control the abnormal transcription of oncogenes [29]; thus, we sought to determine whether activation of SEELA is associated with the MLL fusion protein-BRD4 complex. When cells were treated with i-BET151, the binding of the MLL fusion protein, BRD4, and POL II at the *SEELA* locus was dramatically reduced (Fig. 1f). The *MYC* and *B2M* genes were used as positive and negative controls, respectively, according to a previous report [23] (Additional file 2: Fig. S2H). These results indicated that abnormal transcription of SEELA was induced by the MLL fusion-BRD4 complex via recruitment of POL II to the enhancer locus.

### SEELA binds histone components to activate the target gene *SERINC2 in cis*

**Fig. 2:**
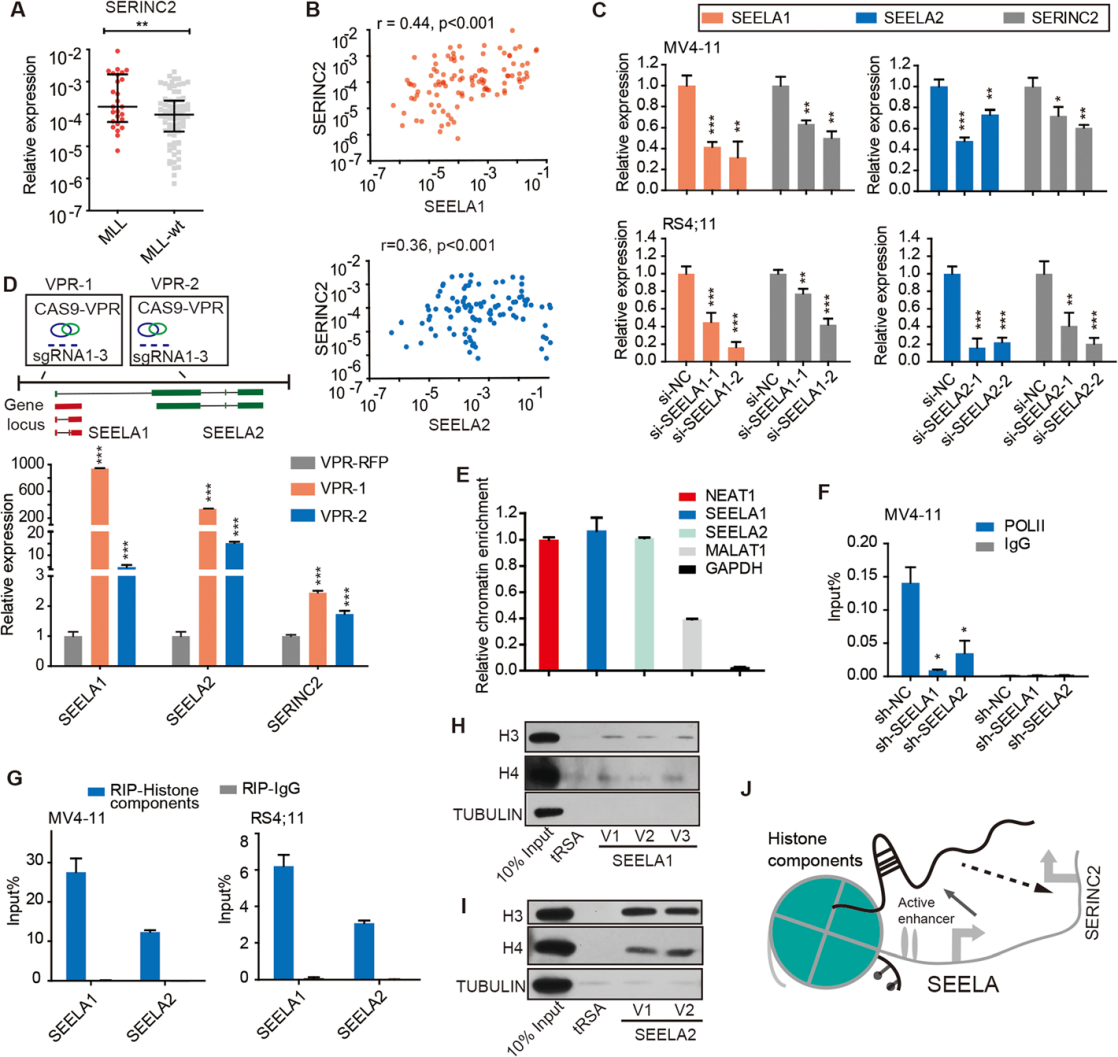
Lnc-eRNA SEELA binds histone components to activate target genes *in cis*. **a** The expression level of SERINC2 was significantly upregulated in patients with *MLL* leukemia (*n* = 26) compared with *MLL*-wt (*n* = 75) patients (Mann-Whitney test, **, *P* < 0.01). The expression level was calculated using 2^-ΔCT^ method and was normalized to GAPDH. **b** Positive correlation of SERINC2 with SEELA was observed in patient samples (*n* = 101). Spearman analysis was used, and *r* = 0.44, *P* < 0.001 (upper) and *r* = 0.36, *P* < 0.001 (bottom). **c** qPCR showed that the mRNA levels of SERINC2 were decreased after knocking down SEELA. Error bars reflect ±SD (**P < 0.01; ***P < 0.001) in three independent experiments. **d** A schematic diagram shows the *SEELA* sites within their loci targeted by sgRNAs of the CRISPR-VPR activation system (upper). qPCR showed that the mRNA levels of SERINC2 and SEELA were significantly upregulated (bottom). Error bars reflect ±SD (**P < 0.01; ***P < 0.001) in three independent experiments. **e** The SEELA was enriched in the chromatin fraction of MV4-11 cells. The relative concentration was adjusted according to the chromatin concentration of positive control NEAT1. MALAT1 was another positive control and GAPDH was negative control. Error bars reflect ± SD in three independent experiments. **f** ChIP-qPCR showed the decreased POL II binding to the SERINC2 promoter after knocking down SEELA. Error bars reflect ±SEM (*P < 0.05) in three independent experiments. An IgG antibody was used as a negative control. **g** RIP-qPCR detection was used to assess the association of histone components with SEELA in MV4-11 and RS4;11 cells. Error bars reflect ± SEM in three independent experiments. An IgG antibody acted as a negative control. **h**, **i** Immunoblot detection of H3, and H4 retrieved by in vitro-transcribed tRSA-tagged SEELA sections from MV4-11 cell lysates. SEELA1 (SEELA1-V1/2/3) and SEELA2 (SEELA2-V1/2) presented significant enrichment. TRSA and beta-tubulin were used as negative controls. **j** A schematic diagram shows that upregulated lnc-eRNA SEELA acted *in cis* to activate *SERINC2* transcription via binding with histone components

Since SEELA was transcribed from the enhancer region of *SERINC2*, we investigated the relationship between SEELA and SERINC2. A positive correlation between SERINC2 and SEELA expression was observed in clinical samples (Fig. 2a, b) and cell lines (Additional file 2: Fig. S3A). This correlation was further validated by modulating the expression of the SEELA (Fig. 2c, d; Additional file 2: Fig. S3B, C). Interestingly, knockdown SEELA1 or SEELA2 by siRNA, respectively, could reduce the expression of each other (Additional file 2: Fig. S3D), further supporting that this eRNA cluster is transcribed coordinately. Furthermore, silencing SEELA had little effect on the expression of two other nearby genes (*FABP3* and *TINAGL1*) that were located near the *SEELA* locus (Additional file 2: Fig. S3E), indicating that SEELA mainly regulated the expression of SERINC2.

The majority of eRNAs are located preferentially on chromatin and function locally to regulate the transcription of neighboring genes [13], although the mechanism remains unclear. We firstly investigated whether SEELA functions at the chromatin level. SEELA was retained in the nuclear fraction (Additional file 2: Fig. S3F) and enriched in the chromatin fraction (Fig. 2e). Binding of POL II at the transcription start site of *SERINC2* was reduced after knockdown of these lnc-eRNAs (Fig. 2f), indicating that SEELA transcriptionally activated *SERINC2* expression *in cis*. We next sought to determine the mechanism by which the lnc-eRNAs are enriched in the chromatin fraction. Given that histones are the main component of chromatin, we detected whether lnc-eRNAs bind chromatin by interacting with histone components. RIP analysis using anti-histone H3 antibodies showed that SEELA was highly enriched in the histone component immunoprecipitation samples (Fig. 2g; Additional file 2: Fig. S3G, H), whereas two other highly expressed lncRNAs, HOTAIRM1 and ENST00000413525, used as negative RNA controls, were not associated with histone components (Additional file 2: Fig. S3I). A tRSA RNA pull-down assay [30] using the full-length sequences of all variants of the two lnc-eRNAs further confirmed that SEELA interacted with histones H3 and H4 (Fig. 2h, i). Collectively, these results suggested that the upregulated SEELA acted *in cis* by binding with histone components, as shown in Fig. 2j.

### SEELA directly binds K31 amino acid of histone H4

We next sought to determine which histone component binds SEELA. As SEELA is transcribed from loci enriched in histone marks H3K27ac and H3K4me1, we speculated that SEELA might bind these two modifications. Unexpectedly, no preferential binding to H3K27ac or H3K4me1 was observed (Additional file 2: Fig. S4A). Additionally, we used SEELA1-variant 1 (V1) to further test its ability to bind to H3K4me1 and H3K27ac in RNA, and no difference was observed in the SEELA1 pull-down assay when we mutated K4, K27, and a control site (K36) from K residues to M residues (Additional file 2: Fig. S4B), suggesting that enhancer modification might not be the essential histone characteristic required for SEELA binding.

**Fig. 3:**
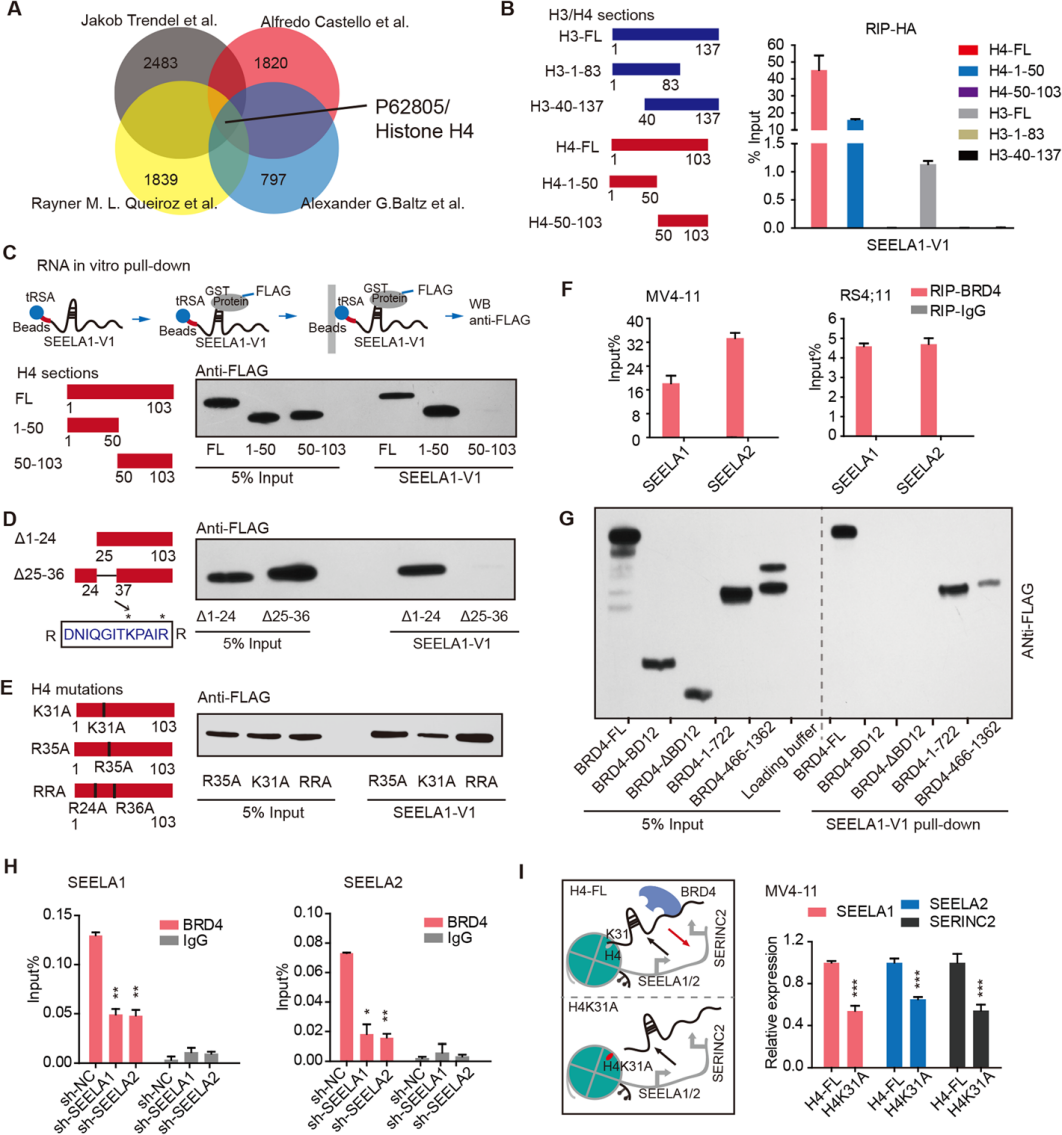
SEELA binds K31 aa of histone H4 to promote the enhancer recognition of histone modification reader. **a** The reported RNA-binding proteomes identified histone H4 (P62805) as a RNA-binding protein candidate. **b** A schematic diagram shows the truncated fragments of H3 and H4 (left). RIP-qPCR for SEELA1-V1 pull-down by HA-tagged H3 and H4 sections in 293 T cells (right). The anti-HA antibody was used in RIP-qPCR assays. **c**–**e** The in vitro tRSA RNA pull-down assay with the purified different mutants of H4 proteins showed that the K31 aa of H4 mainly interacted with SEELA1-V1. **f** RIP-qPCR detection was used to assess the association of BRD4 with SEELA in MV4-11 and RS4;11 cells. Error bars reflect ± SEM in three independent experiments. An IgG antibody acted as a negative control. **g** Immunoblot detection of Flag-tagged BRD4 truncated fragments retrieved by *in vitro*-transcribed tRSA-tagged SEELA1-V1 from 293 T cell lysates. BRD4 (aa 1-1362, aa 1-722, and aa 466-1362) presented significantly higher enrichment of SEELA1-V1. **h** ChIP-qPCR assays after knocking down SEELA showed that the enrichments of BRD4 at the *SEELA* locus were downregulated in MV4-11. Error bars reflect ±SEM (*P < 0.05; **P < 0.01) in three independent experiments. An IgG antibody was used as a negative control in the ChIP assay. **i** A schematic diagram (left) shows stable overexpressed HA-tagged H4-full length or H4K31A into MV4-11 cells. The binding of SEELA is disrupted in H4K31A mutants, as a result, the missing of the SEELA-induced SERINC2 expression. The expression of SEELA and SERINC2 (right) were downregulated in H4K31A mutant expression cells versus that in the H4-full length expression cells. Error bars reflect ± SD (***P < 0.001) in three independent experiments

We then sought to determine whether lnc-eRNAs interact with specific proteins of histone components. By scanning the available public RNA-binding proteomes [31–34], we found that four independent studies identified histone H4 (Fig. 3a) as an RNA-binding protein. RIP assays with HA-tagged histone H4 or H3 fragments as bait showed that only the H4 fragment with amino acids 1-50 (H4, 1-50 aa) coprecipitated with SEELA1 (Fig. 3b and Additional file 2: Fig. S4C), implying that SEELA1 binds to histone H4 (1-50 aa). However, a discrepancy was found between the binding of SEELA to truncated 1-50 aa and full-length H4. This difference could be the reason that SEELA is associated with chromatin and that truncated H4 might not incorporate into nucleosomes as efficiently as full-length and, hence, might bind less SEELA than full-length H4. But we could not fully exclude the potential contribution of the C terminus of histone H4 in binding SEELA.

In vitro RNA pull-down assays showed that SEELA1, rather than tRSA or SEELA1 antisense RNA, coprecipitated with full-length H4 (Additional file 2: Fig. S4D). Additionally, neither recombinant GST protein nor GST-tagged full-length H3 protein interacted with SEELA1 (Additional file 2: Fig. S4E). Furthermore, the 1-50 aa region of H4 showed high affinity for SEELA1 (Fig. 3c), indicating that SEELA1 directly binds the 1-50 aa of H4. Using the histone H4 truncation mutants (H4-Δ1-24, H4-Δ25-36), we found that the 25-36 aa region of H4 directly interacted with SEELA1 (Fig. 3d). The amino acid sequence of this region is “DNIQGITKPAIR,” and arginine (R) and lysine (K) are amino acids that preferentially bind RNA [35]. Substitution of K31 of H4 with alanine decreased the interaction of H4 with SEELA1, but mutation of either R35 or the control amino acids (R24 and R36) did not affect the binding of H4 to SEELA1 (Fig. 3e). Consistent with this result, the RIP assay showed that mutation of K31 significantly reduced the association between H4 and SEELA1 (Additional file 2: Fig. S4F), suggesting the importance of H4K31 in mediating RNA binding. Among all histone components, the histone H4 protein is highly conserved, and H4K31 is conserved among almost all types of eukaryotes, including nematodes, drosophila, plants, and human (Additional file 2: Fig. S4G). This conservation suggests that the RNA interaction domain of H4 may play a pivotal role in eukaryotic histone function. The nucleosome is an octamer containing two copies of each of the core histone proteins (H2A, H2B, H3, and H4) [36]. H4K31 lies at the N-terminus of the histone H4α1 helix and is exposed on the outer surface of the nucleosome (as shown in Additional file 2: Fig. S4H). The location of H4K31 may provide the space for the SEELA-H4 interaction, which could be a possible mechanism for the specificity of H4K31 in lnc-eRNA binding.

### SEELA acts as a modular scaffold to promote enhancer recognition of histone modifiers

The observation above showed that lnc-eRNAs bind histone H4 to achieve specific chromatin localization, which is the premise for their function in activating nearby genes *in cis*. We next sought to determine how lnc-eRNAs affect enhancer activity to regulate transcription. Enhancer activity is driven by binding of histone modifiers. Notably, the majority of these modifiers and readers, including MLL3/4, CBP/P300, TIP60, CHD7, and BRD4, were identified in previous studies to have RNA binding ability [31–34, 37, 38]. Thus, we speculated that H4-binding lnc-eRNAs may act as the key mediators to strengthen histone modifier recruitment to affect enhancer activity and oncogene activation. To address this question, we selected BRD4 to verify its interaction with SEELA. BRD4 is the transcriptional regulator of SEELA, making it more spatially accessible to SEELA. We found that BRD4 directly bound to SEELA in the RIP and tRSA pull-down assays (Fig. 3f and Additional file 2: Fig. S5A-D). Specifically, pull-down of SEELA1 with a series of truncated BRD4 fragments showed that SEELA1 might bind the 466-722 aa region of BRD4 (Fig. 3g and Additional file 2: Fig. S5E). Importantly, BRD4 enrichment at enhancer loci was significantly downregulated after knockdown of SEELA1 or SEELA2 (Fig. 3h), suggesting that SEELA bound histone H4 to influence histone modification reading via BRD4 recruitment. Furthermore, we constructed stable MV4-11 cells overexpressing HA-tagged H4-FL or H4K31A. Interestingly, the expression of SEELA and SERINC2, but not FABP3 and TINAGL1, were reduced in H4K31A-expressing cells, indicating that binding of SEELA to H4K31 sustained the expression of SEELA-SERINC2 axis components (Fig. 3i and Additional file 2: Fig. S5F,G). Notably, the SEELA binding site was close to the histone H4 tail, and the acetylated histone H4 tail was found to be a BRD4 binding site, which supported our finding that SEELA acted as a modular scaffold by interacting with the 466-722 aa of BRD4 and K31 aa of histone H4 (Additional file 2: Fig. S5H), implying an important role for lnc-eRNAs. Together, these data suggested that SEELA bound to K31 aa of histone H4 and subsequently induced the occupancy of histone modification reader on chromatin to promote enhancer activity.

### SEELA promotes leukemia progression by affecting the transcription of the oncogene *SERINC2* and, in turn, regulating oncometabolites

**Fig. 4:**
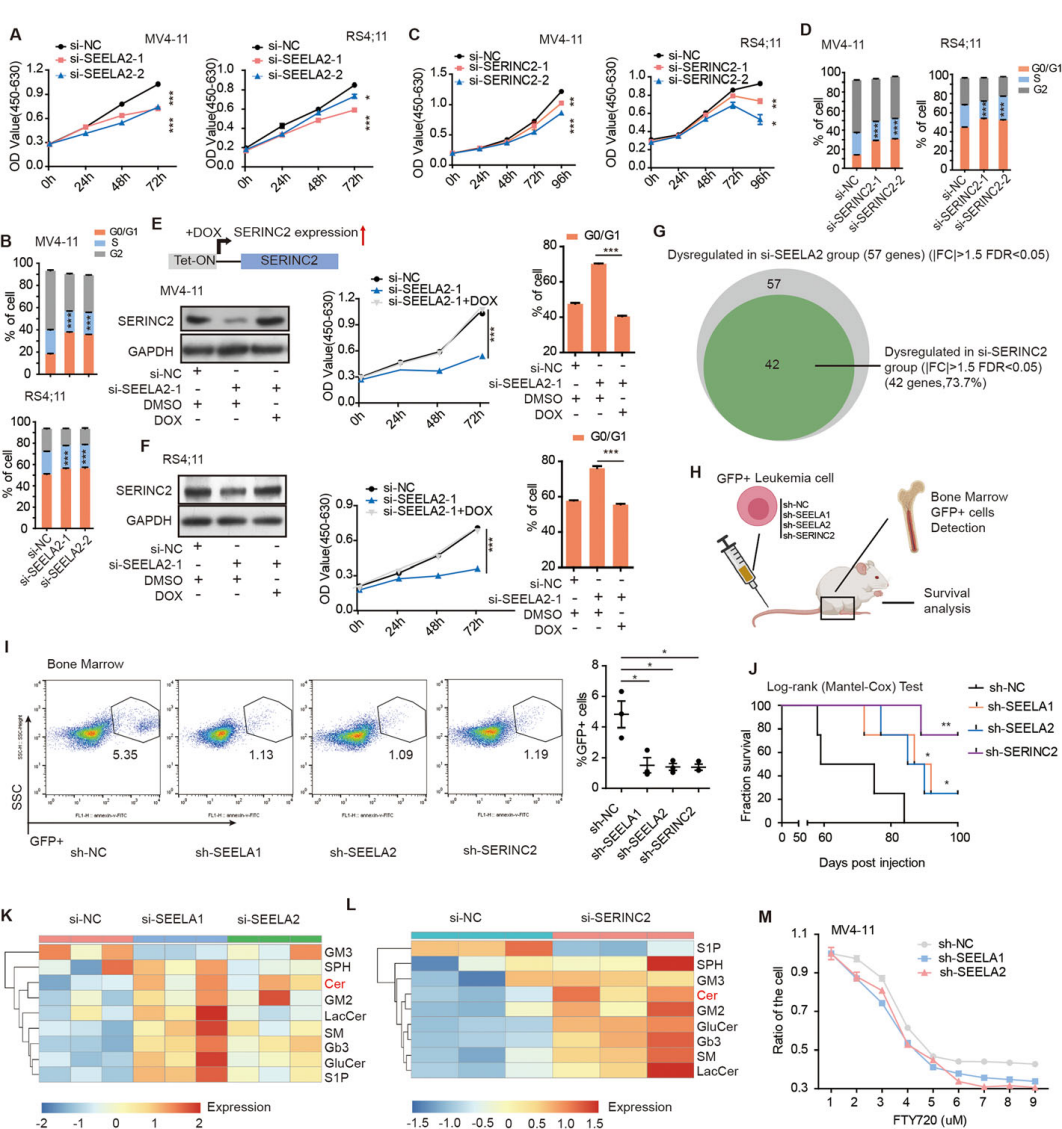
SEELA-SERINC2 axis promotes leukemia progression in vitro and in vivo through sphingolipid metabolism. a-d Inhibition of SEELA2 (**a**, **b**) and SERINC2 (**c**, **d**) disrupted the cell proliferation and induced the accumulation of G0/G1 phase cells via CCK-8 assay and cell cycle assay by flow cytometry in MV4–11 and RS4;11 cells. Error bars reflect ± SEM (*P < 0.05; **P < 0.01; ***P < 0.001) in three independent experiments. **e**, **f** Schematic outline of SERINC2 rescue strategy (e left upper) in MV4-11 (**e**) and RS4;11 (**f**) cells. Western blot showed the protein level of SERINC2 in indicated cells. CCK8 and cell cycle assay showed the cell proliferation and cell cycle arrest of indicated treatment. The error bars indicate the ± SEM values (***P < 0.001) in three independent experiments. **g** Total 57 genes were dysregulated in the si-SEELA group (|FC| > 1.5, FDR < 0.05), and 42 of 57 genes (73.7%) were dysregulated si-SERINC2 group (|FC| > 1.5, FDR < 0.05). **h** A schematic diagram shows the xenotransplantation model. **i** Representative flow cytometry graphs show the decreased percentages of human leukemic GFP+ cells in the bone marrow from mice treated with sh-SEELA, and sh-SERINC2 treated MV4-11 cells relative to the levels observed in control mice (left). A scatter plot shows the statistical values (right). Error bars reflect ± SEM (*P < 0.05). **j** Kaplan-Meier survival curves show that the mice injected with sh-SEELA and sh-SERINC2 survived longer than those of the control sh-NC group (*n* = 4). The *P* values were calculated using a log-rank (Mantel-Cox) test. (*P < 0.05; **P < 0.01). **k**, **l** Heatmaps show the expression of sphingolipids from the mass spectrometry (MS) assay. Most of the sphingolipids were upregulated after knocking down SEELA (si-SEELA1-1 or si-SEELA2-1) (**k**) or SERINC2 (si-SERINC2-1) (**l**) in MV4-11 cells. Ceramides, Cer; sphingomyelins, SM; glucosylceramides, GluCer; lactosylceramides, LacCer; ganglioside, GM2; monosialo-dihexosyl gangliosides, GM3; trihexosylceramide, Gb3; sphingosine, SPH; and sphingosine-1-phosphate, S1P. **m** The survival rate of MV4-11 cells was measured using a CCK-8 kit at 24 h. The sh-NC, sh-SEELA established cells were treated with FTY720 at concentrations from 1 to 9 μM

The observations above showed that SEELA modulated enhancer activity to transcriptionally activate *SERINC2* expression (Figs. 2 and 3). The SERINC family has been suggested to be involved in the synthesis of serine-derived lipids [39], including sphingolipids, which are frequently reported as oncometabolites in cancer progression [40]. Thus, we hypothesized that SEELA may exert its biological effects by activating *SERINC2* expression. To address this question, we performed in vitro and in vivo functional studies. As shown in Fig. 4a–d and Additional file 2: Fig. S6A-C, suppressing SEELA or SERINC2 reduced leukemic cell proliferation and inhibited cell cycle progression. To further demonstrate the *cis* effect of SEELA on SERINC2 expression and leukemia progress, rescue experiments were performed. We overexpressed SERINC2 in the SEELA knockdown cells (si-SEELA2-1) via doxycycline (Dox) induction and lentiviral systems and found that the proliferation inhibition and cell cycle arrest caused by transfection of SEELA siRNAs were reversed (Fig. 4e, f and Additional file 2: Fig. S6D). Additionally, we introduced an eGFP-tagged SERINC2 protein into si-SEELA cells to distinguish its expression from the endogenous protein and found that it could restore the effect of si-SEELA (Additional file 2: Fig. S6E). These results suggested that SERINC2 is an important downstream target of SEELA. To further investigate whether SEELA exerts its oncogenic effects mainly through modulating SERINC2 expression, we performed RNA-seq in MV4-11 cells transfected with si-NC or si-SEELA2-1. Comparing with si-NC, 57 genes were dysregulated in the si-SEELA samples with absolute value of fold change > 1.5 (|FC| > 1.5), FDR < 0.05. In the si-SEELA samples, SERINC2 was significantly downregulated (log2 fold change = − 1.3, FDR = 8.64 × 10^−10^), while the other two neighbor genes *FABP3* or *TINAGL1* did not show obvious variation (Additional file 2: Fig. S6F), consistent with our previous qPCR results. We have also performed RNA-seq in the cells transfected with si-SERINC2-1 in order to see how many dysregulated genes of si-SEELA group are altered in si-SERINC2 group. We found that 42 of 57 dysregulated genes (73.7%) in si-SEELA group were also found to be dysregulated in si-SERINC2 group (|FC| > 1.5, FDR < 0.05) (Fig. 4g and Additional file 3: Table S2), indicating that SEELA and SERINC2 could affect a large number of same downstream gene sets. Based on the RNA-seq data, it can be inferred that SEELA regulates its downstream genes mainly through modulating SERINC2.

We further used a NOD-SCID mouse model to explore the functions of SEELA and SERINC2 (Fig. 4h) in MV4-11 cells transfected with different shRNAs (the effect of SERINC2 shRNA is shown in Additional file 2: Fig. S6G)*.* The percentages of human leukemic GFP+ cells were decreased in the bone marrow (Fig. 4i) of mice implanted with sh-SEELA and sh-SERINC2 MV4-11 cells compared with mice in the sh-NC cell group. In addition, mice in the sh-SEELA1 (*P* < 0.05), sh-SEELA2 (*P* < 0.05), and sh-SERINC2 (*P* < 0.01) groups survived longer than those in the control sh-NC group (Fig. 4j), showing that SEELA and SERINC2 affect leukemia progression in vitro and in vivo.

We then investigated whether SEELA-SERINC2 axis could influence sphingolipid synthesis to drive leukemia progress. Unbiased sphingolipid profiling by mass spectrometry (MS) was performed in MV4-11 cells with suppression of SEELA and SERINC2 expression. The altered profile of sphingolipid metabolism in SEELA knockdown cells strongly resembled that in SERINC2 knockdown cells (Fig. 4k, l and Additional file 2: Fig. S7A, B), further indicating that SERINC2 is the essential downstream effector of SEELA that influences sphingolipid synthesis and leukemia progression. Importantly, ceramides (Cer), tumor-suppressor sphingolipids [41], were found to be upregulated when either SEELA or SERINC2 was knocked down (Additional file 2: Fig. S8A, B). Suppression of SEELA expression also resulted in the sensitivity of MV4-11 cells to FTY720 (Fig. 4m and Additional file 2: Fig. S8C-E), an anticancer drug that induces cell death through ceramide accumulation [42, 43] indicating that the SEELA-SERINC2 axis affects leukemia progression by mediating ceramide accumulation.

### The enhancer activity of *SEELA* is programmed via distal binding of HOXA9/10

**Fig. 5:**
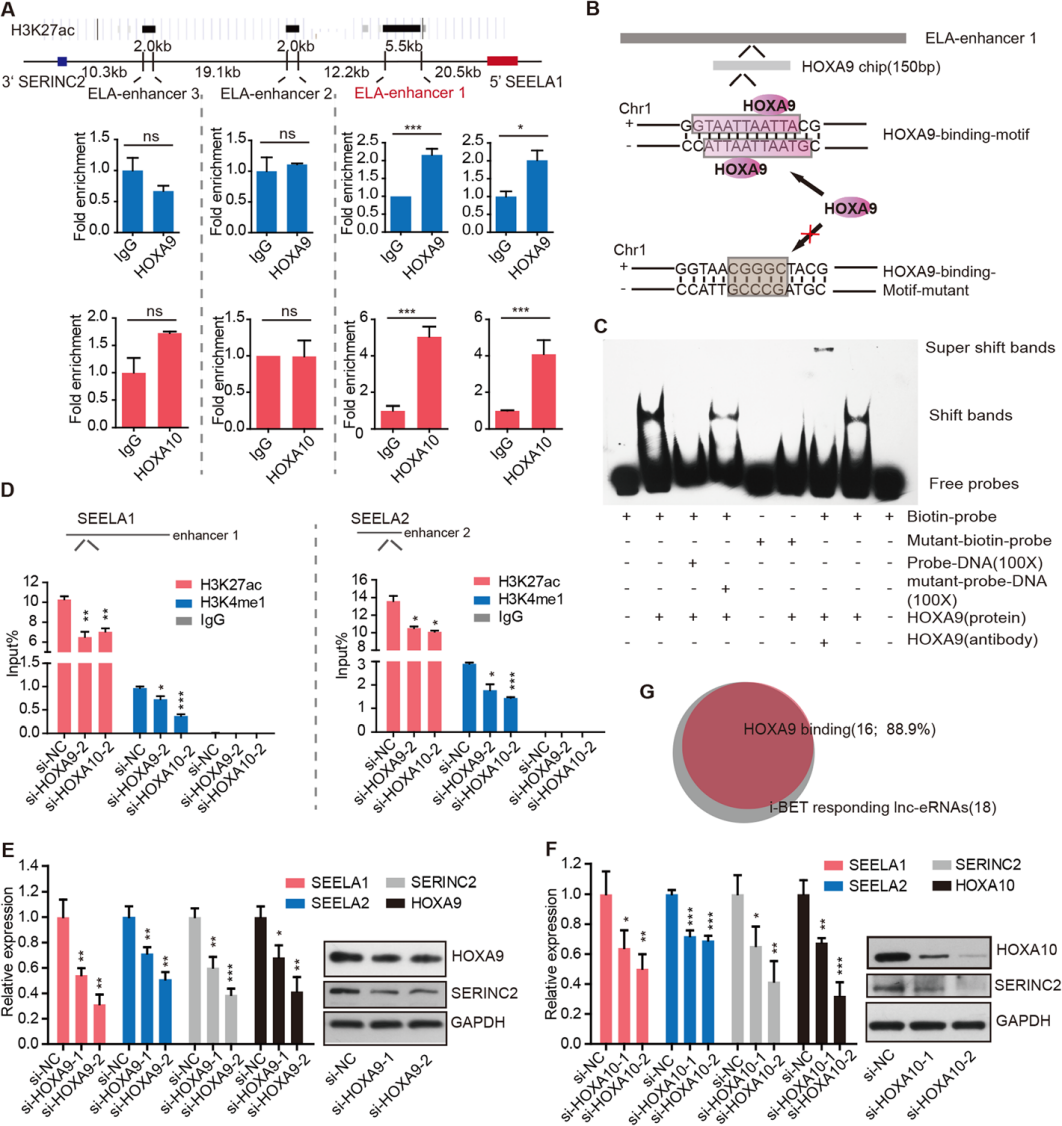
HOXA9/HOXA10 binds to the upstream enhancer of *SEELA* to reprogram the enhancer-related histone mark. **a** A schematic diagram shows the genomic location and the H3K27ac enrichment of ELA-enhancer 1/2/3 (located at the region 20.5 kb, 38.2 kb, and 59.3 kb upstream of *SEELA1*, respectively). ChIP-qPCR assays of HOXA9 (middle) and HOXA10 (bottom) showed that HOXA9/HOXA10 bound to ELA-enhancer 1 instead of ELA-enhancer 2/3; Error bars reflect ± SD (*P < 0.05; ***P < 0.001; ns, no significant) in three independent experiments. An IgG antibody was used as negative controls in the ChIP assay. **b**, **c** A schematic diagram shows the normal and mutant DNA sequences that are predicted in HOXA9 located (**b**). EMSA experiments validated the binding motif of HOXA9/10, which was a 14 bp motif (GGTAATTAATTACG) (**c**). **d** ChIP-qPCR assay results of H3K27ac and H3K4me1 after knocking down HOXA9 and HOXA10, the enrichment of enhancer histone marks in the *SEELA* locus were decreased. Error bars reflect ± SEM (*P < 0.05, **P < 0.01, and ***P < 0.001) in three independent experiments. An IgG antibody was used as a negative control in the ChIP assay. **e**, **f** qPCR and western blotting showed that the mRNA and protein levels of SEELA and SERINC2 were reduced after knocking down HOXA9 (**e**) or HOXA10 (**f**). Error bars reflect ± SD (*P < 0.05, **P < 0.01, and ***P < 0.001) in three independent experiments. **g** Analysis of the chip-seq data showed that 16 of 18 i-BET responding lnc-eRNAs loci were marked by HOXA9

Lnc-eRNAs have been shown to be transcribed from established active enhancer loci. We finally sought to determine the mechanism by which the enhancer activity at lnc-eRNAs loci is programmed in *MLL* leukemia. Given that *HOXA9*, the most important downstream gene in *MLL* leukemia, was reported to have enhancer-binding preference [44] and to rewire leukemia-specific enhancers [25], we speculated that *HOXA* genes might mediate the programming of the *SEELA* enhancer. H3K27ac ChIP-seq identified that three regions located upstream of *SEELA1* were marked by H3K27ac. We named these regions SEELA-enhancer1/2/3 (ELA-enhancer1/2/3). ChIP-qPCR showed that HOXA9/10 preferentially bound ELA-enhancer1 (Fig. 5a) rather than ELA-enhancer2/3 or the *SEELA* locus (Additional file 2: Fig. S9A). Additionally, a 150 bp DNA region within ELA-enhancer1 was enriched with HOXA9, as identified in the ChIP-seq data. Notably, a 14 bp binding motif of HOXA9/10 was found in this region using the JASPAR prediction tool [45] (Additional file 2: Fig. S9B, C). The region contained two HOXA9/10 binding motifs on the two strands due to the opposing position of the DNA strands (Fig. 5b). EMSA was performed to validate the binding of HOXA9 to this motif (Fig. 5c). The levels of H3K27ac and H3K4me1 histone modifications at the *SEELA* locus were reduced after knockdown of HOXA9 and HOXA10 (Fig. 5d), which was followed by a significant reduction in the expression of both SEELA and SERINC2 but not the other two nearby genes *FABP3* and *TINAGL1* (Fig. 5e, f and Additional file 2: Fig. S9D). Collectively, these results indicated that HOXA9/10 controls *SEELA* transcription by programming enhancer activity. To further explore whether HOXA9/10 initiates enhancer-driven activation of global lnc-eRNA loci, we reanalyzed ChIP-seq data of HOXA9 [46] in MV4-11 cells and found that 16 of 18 i-BET-responding lnc-eRNA (Fig. 1b) loci exhibited HOXA9 enrichment (Fig. 5g and Additional file 4: Table S3), implying that most of these lnc-eRNAs could be activated via this HOXA initiation-BRD4 transcriptional activation mechanism and that modulating the expression of these lnc-eRNAs may be an alternative strategy to target the *HOXA* cluster [47] in *MLL* leukemia.

**Fig. 6:**
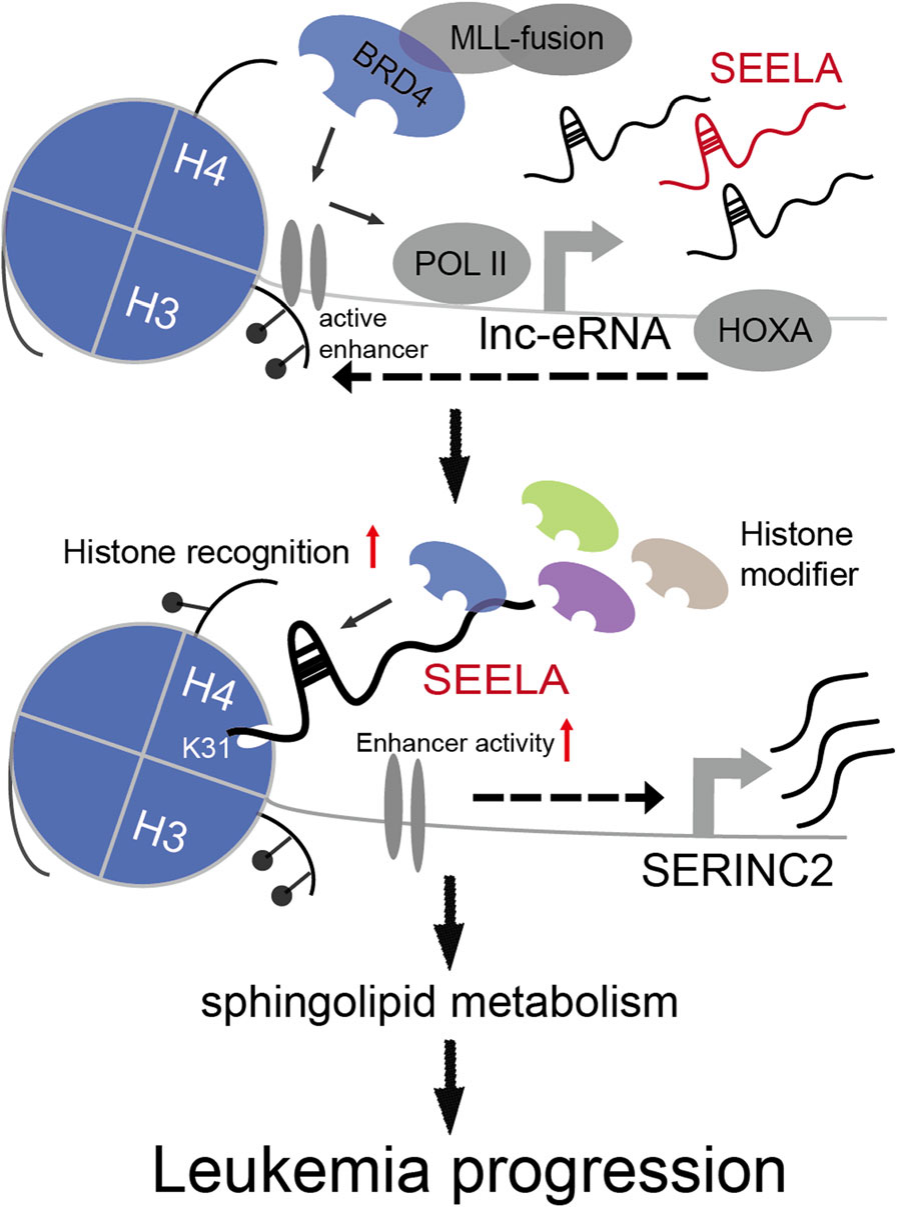
A working model shows the proposed activation and function of lnc-eRNAs

Collectively, our data suggest a model of the activation and function of lnc-eRNAs in leukemia progression. As shown in Fig. 6, lnc-eRNAs are activated via two steps: the HOXA cluster induces enhancer activity by modulating epigenomic signatures, and BRD4 binds histone acetylation marks to promote lnc-eRNA transcription. The upregulated lnc-eRNA SEELA enhances the chromatin occupancy of histone modifiers by directly interacting with K31 aa of histone H4, thereby promoting enhancer activity and SERINC2 expression to affect sphingolipid metabolism during leukemia progression.

## Discussion

Genome-wide expression analysis revealed that lncRNAs are pervasively dysregulated in various types of cancer. However, the biological functions and mechanisms of most lncRNAs are largely unclear. Because of the broad definition of lncRNA, lncRNAs are heterogeneous in their biogenesis, evolution and modes of action [48, 49]. Thus, classifying lncRNAs into subgroups is the first step in investigating lncRNAs. In this study, we identified a large portion of upregulated lncRNAs (41 of 128, 32.0%) as lnc-eRNAs and indicated that these lnc-eRNAs may play roles in *cis* activation of oncogenes in *MLL* leukemia. We further showed that a set of lnc-eRNAs were activated through a HOXA initiation-BRD4 transcriptional activation mechanism, by which the HOXA cluster promotes enhancer histone modification programming, and the epigenetic reader BRD4 cooperates with the coregulator MLL fusion oncoprotein to induce transcriptional activation. Remarkably, as an important regulatory transcription factor in *MLL* leukemia, HOXA9 is reported to rewire leukemia-specific enhancers [25]. However, whether it functions in lnc-eRNA activation remains largely unknown. Our findings provide insight into the regulatory mechanism by which *HOXA* genes and BRD4 activate lnc-eRNAs in the pathogenesis of aggressive hematologic malignancies.

Localization to a precise genomic locus is regarded as one of the key mechanisms by which lncRNAs can regulate gene expression in the nucleus [50]. Although the majority of lnc-eRNAs have been reported to act locally to regulate the transcription of neighboring genes, the mechanisms that control chromatin occupation of lnc-eRNAs remain unknown. Several explanations have been proposed. One possibility is that lncRNAs might identify and interact with regulatory target sites on chromatin via affinity interactions with chromatin-binding proteins [50]. For instance, the lncRNA XIST is tethered to chromatin by interacting with the nuclear matrix protein SAFA; however, SAFA localizes not only on the X chromosome but also on autosomes [51, 52]. Therefore, affinity interactions to chromatin-binding proteins cannot fully account for the specific localization of lncRNAs to precise loci. Another explanation was recently proposed: a study reported that the U1 snRNP interacts with transcriptionally engaged POL II to tether and mobilize lncRNAs to chromatin in a transcription-dependent manner [53]. In this study, we demonstrated that lnc-eRNAs bound histone H4, which seemed to control the chromatin retention and *cis* regulation function of lnc-eRNAs. This is the first time, to our knowledge, that a direct interaction between lnc-eRNAs and histone H4, the main protein component of chromatin, has been reported. These results indicate that lncRNAs are recruited to their regulatory sites by multiple mechanisms.

The biogenesis of eRNAs stems from a number of transcriptional coregulators that bind to H3K27ac through bromodomain (BD) modules to affect chromatin accessibility and transcriptional activation at active enhancers. However, the affinity and selectivity of BD module interactions with acetylated lysines are typically weak [54–56], suggesting that additional mechanisms regulating BD binding at enhancers are required and have important implications for gene regulation. In this study, we showed that two lnc-eRNAs, SEELA1 and SEELA2, bind to histone H4 and subsequently enhance the occupancy of BRD4 on chromatin, which is important for the expression of both SEELA and the downstream target gene *SERINC2*. Thus, we speculate that this BRD4–lnc-eRNA loop enables enhanced chromatin engagement and transcriptional activation. Intriguingly, the binding motif of BRD4, as we found here to be the 466-722 aa region, was different from the previously reported eRNA-interacting domain (1-466 aa) [57], implying that the 466-722 aa region could be an alternative target for new BRD4 inhibitors to affect enhancer activity. Given the newly discovered ability of histone H4 to bind RNA, we believe that histone H4 provides the physical link that tethers lnc-eRNAs to the locus where they are transcribed and guarantees the proper localization of lnc-eRNAs, which is crucial for their functions, such as promoting histone recognition, as we reported here.

*MLL* leukemia is one of the most aggressive leukemia subtypes with the poorest prognosis [58]. Most of the MLL fusion partners are members of the super elongation complex (SEC), a critical regulator of transcriptional elongation [59], suggesting that aberrant control of this process has an important role in leukemogenesis. Our results provide a direct link between the MLL fusion oncoprotein and enhancer transcription, which implies an additional regulatory pathway by which MLL fusion drives leukemogenesis. Importantly, the MLL-BRD4 complex binds to histone acetylation marks to recruit POL II in order to control transcriptional elongation [23], and a small molecule inhibitor of BRD4 (i-BET) has shown promise for use in *MLL* leukemia therapy. We reported that lnc-eRNAs, such as SEELA, are tethered by histone H4 and act as the “assistants” to bind BRD4, facilitating the activation of *SERINC2* by the MLL-BRD4 complex. Our results suggested that lnc-eRNAs are the pivotal mediators of MLL-BRD4-driven oncogene activation, highlighting the potential roles of dysregulated lncRNAs in *MLL* leukemia progression [28, 60]. Furthermore, targeting metabolism is becoming an important strategy for treating diverse cancers [61], and several lncRNAs have been reported to be associated with metabolic pathways [62, 63]. Our study revealed that the lnc-eRNA SEELA influences the biosynthesis of sphingolipids through SERINC2 to promote leukemia progression. This is the first report of the regulation of sphingolipid metabolism by lncRNAs and further implies that targeting sphingolipid metabolism may be an alternative approach to treat *MLL* leukemia. It was worth mentioning that, although the RNA-seq results showed that SEELA and SERINC2 could affect a large number of same downstream genes, we also observed that 8 of the 57 (14.0%) (Additional file 3: Table S2) genes were dysregulated in si-SEELA rather than in si-SERINC2 samples. These 8 genes reside on different chromosome or chromosome arm from *SEELA* locus, showing that they might not be regulated by the SEELA *in cis*. Thus, we cannot exclude whether the lnc-eRNA SEELA could also play a regulatory role on other genes *in trans* at this stage, future studies are still needed to reveal whether there are additional functional targets of SEELA.

## Conclusion

In summary, we demonstrated that lnc-eRNAs, as a subtype of lncRNAs, are dysregulated and functionally active in hematologic malignancies. Importantly, we demonstrated that the lnc-eRNA SEELA is localized on chromatin to induce *cis*-activated expression of the oncogene *SERINC2* via directly binding with K31 aa of histone H4 to strengthen the recognition of histone modifiers. Our results revealed a model for understanding *cis* regulation of lnc-eRNA function in cancer initiation and progression.

## Methods

### Leukemia patient samples collection

The clinical leukemia samples were obtained at the time of diagnosis and with informed consent from the First Affiliated Hospital of Sun Yat-sen University. Sample collection was approved by the Hospital’s Protection of Human Subjects Committee. The detail clinicopathological characteristics of the patients were summarized in Additional file 5: Table S4. The leukemia samples were stored in liquid nitrogen until used.

### Cell culture

Human *MLL* leukemia cells MV4-11, RS4;11 and human embryonic kidney cell line HEK293T cells were purchased from American Type Culture Collection (ATCC, USA). RS4;11 cells were cultured in RPMI-1640 medium (HyClone, USA); MV4-11 cells were cultured in IMDM (HyClone, USA), and HEK293T cells were cultured in DMEM (Gibco, USA). These cell mediums were supplemented with 10% FBS (HyClone, USA). All cells were cultured at 37 °C in a 5% CO2 atmosphere.

### Cell treatment and transfection

The BRD4 inhibitor i-BET151 (Selleck, S2780) was treated in MV4-11 cells at a concentration of 1 μM and the cells were collected after 12 h. The FTY720 (Selleck, S5002) was treated in MV4-11 and RS4;11 cells and the cells were collected after 24 h. The doxycycline (Selleck, S4163) was treated in doxycycline-inducible SERINC2 expression MV4-11 and RS4;11 cells at a concentration of 4 μM. SiRNAs or SERINC2-expressed plasmid were transfected into 4 × 10^5^ cells at a final concentration of 50 nM or 50 ng/μL with Neon™ Transfection System 10 μL Kit using the Neon Transfection System (Invitrogen, USA). HEK293T cells were transfected using the Lipofectamine 2000/3000 (Invitrogen, USA). Cells were collected 48 or 72 h after transfection. The siRNAs sequences were shown in Additional file 6: Table S5.

### RNA isolation and quantitative real-time PCR (RT-PCR)

Total RNA was extracted from bone marrow and cell samples using an Invitrogen™ TRIZOL according to the manufacturer’s instructions. All RNA samples were stored at − 80 °C before reverse transcription and quantitative RT-PCR. RNA was reverse-transcribed into cDNA with the PrimeScript® RT reagent Kit with gDNA Eraser (Takara, Japan). Quantitative RT-PCR for lnc-eRNA and mRNA was performed using the SYBR Premix ExTaq real-time PCR Kit (Takara, Japan) according to the manufacturer’s instructions. All of the data were normalized to GAPDH expression as a control. The expression level for each lnc-eRNA and mRNA was determined using the 2^-△△Ct^ method. The common primers were used to detect the total expression of all the variants SEELA. The primers were shown in Additional file 6: Table S5.

### Plasmid construction

The BRD4-CDS-full length plasmid was purchased from Addgene (#90331). The SERINC2, H3, H4, and BRD4 or H3/H4 mutants were PCR-amplified from MV4-11 cDNA. Then the PCR products were cloned into eukaryotic expression vector pCDH-MSCV-MCS-EF1-Puro-copGFP, pCW57.1 (doxycycline-inducible lentiviral expression of SERINC2) or pcDNA3 (SEELA1-V1). For purification of recombinant proteins in vitro, the full-length cDNA were cloned into pET-N-GST-Thrombin-C-His vector (Beyotime, China). For stable SEELA and SERINC2 knockdown vectors, the DNA oligos encoding shRNAs were synthesized and cloned into the pGreenPuro™ eukaryotic expression vector, sh-NC as the negative control. The primers were shown in Additional file 6: Table S5.

### Lentiviral preparation and infection

Lentivirus carrying shRNAs or SERINC2 CDS was made in the 60-mm culture dish by transfecting packaging cell HEK293T with Lentivector Expression Systems (System Biosciences, Germany) consisting of pPACKH1-GAG, pPACKH1-REV, and pVSV-G. Virus was harvested 48 and 72 h after transfection. Lentivirus Precipitation Solution (System Biosciences, Germany) was used to precipitate virus. The shRNAs sequences were shown in Additional file 6: Table S5. For stable expression assays, 3 × 10^5^ MV4-11 or RS4;11 cells were prepared for each infection system. The cells were centrifuged and resuspended in 300 μL virus suspension, followed by incubation at 37 °C and 5% CO_2_ for 48 h. Then cells were centrifuged washed, and resuspended in fresh medium containing 1 μg/mL puromycin (Selleck, S7417) and 1% penicillin-streptomycin (Thermo Fisher, USA). To confirm target knockdown, cells were collected for qRT-PCR analysis.

### Immunoblotting

Total protein was extracted from cells using RIPA lysis buffer (Beyotime, China) with 1× complete ULTRA (Roche, USA). Proteins were resolved by 10% or 12% Bis-Trispolyacrylamide gels and then transferred to polyvinylidene fluoride membranes. Membranes were blocked in 5% BSA for 1 h, and followed by the appropriate antibody overnight at 4 °C and then incubated with horseradish peroxidase-conjugated secondary antibodies at room temperature for 1 h. Membranes were visualized with an enhanced chemoluminescence detection system. The antibodies were listed in Additional file 7: Table S6.

### Xenotransplantation model

Five-week-old NOD-SCID mice were maintained under specific pathogen-free conditions in the Laboratory Animal Center of Sun Yat-sen University. All experiments on animals were performed according to the institutional ethical guidelines for animal experiments. MV4-11 cells stably expressing sh-NC, sh-SEELA1, sh-SEELA2 or sh-SERINC2 (GFP+ cell populations) were tail vein injected into the mice (3 × 10^6^ cells in 150 μL PBS per mice). Fifty days after inoculation, xenografted mice were sacrificed for analysis. Human cell engraftment (GFP+ cell populations) in bone marrow was evaluated by flow cytometry. The remaining mice were used to perform the survival assay.

### DNA electrophoretic mobility shift assays (EMSA)

The assay was performed using Chemiluminescent EMSA Kit (GS009, Beyotime, China) following the manufacturer’s protocol with slight modifications. In brief, the probe was labeled with biotin and annealed. Two micrograms *Escherichia coli* expressed His-HOXA9 was incubated with 40 nM biotin-labeled probes (or with 1 μm unlabeled probe or mutated probe) in 10 μL 1 × EMSA/Gel-Shift binding buffer at room temperature for 20 min. The samples were then added 1 μL EMSA/Gel-Shift loading buffer (without bromophenol blue) and were separated on 6% PAGE gel. The samples on the gel were then transferred to positive charged Nylon membrane. The membrane was incubated with Streptavidin-HRP Conjugate followed by luminizing with ECL.

### Cell nucleus/cytoplasm and chromatin fraction isolation

Cell nuclear and cytoplasmic fractions were isolated from cell samples using the NE-PER Nuclear and Cytoplasmic Extraction Reagents (Thermo Fisher, USA) according to the manufacturer’s instructions. Cell chromatin fraction was isolated from cell samples according to published protocol [64]. RNA from each fraction was isolated using TRIzol.

### Cell proliferation and cell cycle assay

Cell proliferation was measured using Cell Counting Kit-8 (Dojindo Molecular Technologies, China). Cells were seeded at a density of 20,000 cells per well in 100 μL of complete medium in 96-well plates. Absorbance was measured by a VICTOR™ X5 Multilabel Plate Reader (PerkinElmer, USA) at wavelengths of 480 and 630 nm at 0, 24, 48, 72, and 96 h. For the cell cycle assay, cells were collected and washed once by PBS. Cell pellets were resuspended in 0.5 mL of PI/RNase staining buffer (Dojindo Molecular Technologies, China) and incubated for 30 min at room temperature (RT), and cells were immediately measured and analyzed using flow cytometer (BD Biosciences, USA).

### 5′ RACE and 3′ RACE

Total RNA from MV4-11 cells was extracted using TRIzol according to the manufacturer’s guidelines. The 5′- and 3′-ends of cDNA were acquired using a 5′-FULL RACE Kit with TAP (Takara, Japan) and 3′-FULL RACE Core Set with PrimeScript RTase (Takara, Japan) respectively according to the manufacturer’s instructions. PCR products were obtained and then cloned into pEASY-T3 (TransGen Biotech, China) for further sequencing.

### Protein recombination and purification

Recombinant proteins were expressed in *E. coli* strain BL21 [Transetta (DE3) chemically competent cell (Transgen biotech, CD801)]. In brief, 5 mL Luria-Bertani (LB) culture supplemented with 100 μg/mL ampicillin was inoculated with a single colony at 37 °C. After overnight growth, the culture was diluted 100-fold into 300 mL LB supplemented with 100 μg/mL ampicillin. Protein expression was induced in the presence of 0.4 mM IPTG at 16 °C overnight. Then the cell pellets were collected by centrifugation at 5000 rpm, 4 °C for 10 min and purified recombinant proteins using a His or GST tag Protein Purification Kit (BeaverBeads™) according to the manufacturer’s instruction.

### Chromatin immunoprecipitation (ChIP)

ChIP analyses were performed on chromatin extracts from MV4-11 and RS4;11 cells using a Magna ChIP™ G - Chromatin Immunoprecipitation Kit (17-611) (Merck Millipore, Germany) with primary antibodies (H3K27ac, H3K4me1, H3K4me3, BRD4, MLL, POL II, IgG, HOXA9 and HOXA10) according to the manufacturer’s standard protocol. The antibodies were listed in Additional file 7: Table S6.

### RNA immunoprecipitation (RIP)

In the RIP experiment, anti-FLAG, anti-HA, anti-IgG, anti-BRD4, or anti-H3 antibodies were used along with an EZ-Magna RIP™ RNA-Binding Protein Immunoprecipitation Kit (17-701) (Merck Millipore, Germany) according to the manufacturer’s instructions. In HA-tagged histone H4 or H3 fragments RIP assays, 1 × 10^6^ 293 T cells were transfected into 3 μg H3 or H4 and 200 ng SEELA1-V1 plasmid and collected after 48 h. All proteins for RIP were lysed with cell lysis buffer supplemented with Thermo Scientific™ Halt™ Protease Inhibitor Cocktail (Thermo Fisher, USA). To prepare antibody-coated beads, 50 μL Protein A/G magnetic beads were incubated with 3 μg antibody or control IgG in 500 μL wash buffer at 4 °C for 1 h. Then the beads were washed three times and mixed with the cell lysates in new tubes. The tubes were rotated at 4 °C overnight. Finally, RNA extraction from the beads was further collected by using TRizol according to the manufacturer’s instructions. Reverse transcription and qPCR were performed as previously described. The antibodies were listed in Additional file 7: Table S6.

### CRISPR-VPR activation assay

The sgRNAs targeting *SEELA* locus were cloned into MLM3636 plasmid (Addgene #43860) and co-transfected with SP-dCas9-VPR plasmid (Addgene#63798) into 293 T cells. Cells were collected 48 h after transfection to test the activating effect. The sgRNAs sequences were shown in Additional file 6: Table S5.

### TRSA RNA pull-down assay

The SEELA sequence was cloned into the pcDNA3 plasmid with the tRSA tag at its 5′ end. RNA products were transcribed in vitro using the TranscriptAid T7 High Yield Transcription Kit (Thermo, USA) and were then purified using the GeneJET RNA Purification Kit (Thermo, USA). The RNA pull-down assay was performed using 50 pmol RNA for each sample with the manufacturer’s instructions of Pierce Magnetic RNA-Protein Pull-down Kit (Thermo, USA).

### In vitro RNA pull-down

The 50 pmol tRSA-RNA products were first mixed with 1–3 μg recombinant proteins, and then the mixture was rotated at 4 °C for 1 h in RNA binding buffer according to Pierce Magnetic RNA-Protein Pull-down Kit. Beads were washed three times with RNA wash buffer and then boiled in SDS loading buffer. Finally, the enriched proteins were resolved via SDS-PAGE and analyzed western blotting.

### RNA sequencing

The si-NC, si-SEELA2, and si-SERINC2 samples (5 × 10^6^ cells/sample; three samples each) were obtained from MV4–11 cells. The next-generation sequencing in this study was performed by poly-A RNA-seq on an illumina noveSeq instrument. The sequencing depth of the RNA-Seq is up to 6 Gites in 150-base paired-end mode. For the data analysis, pair-end reads were adapter and quality trimmed using Trim Galore (v0.6.4), and high-quality reads were aligned to reference transcriptome (Gencode v34) and quantified using Salmon (v1.3.0) with fragment GC bias and positional bias corrections [65]. DESeq2 was used for normalizing gene expression level and identifying differentially expressed genes [66].

### Sphingolipid metabolism analysis

The si-NC, si-SEELA and si-SERINC2 sample (2 × 10^6^ cells/sample; three samples each) were obtained from MV4-11 cells. The sphingolipid metabolism (including SM, Cer, GluCer, LacCer, GM2, GM3, Gb3, SPH, S1P) were performed and analyzed by LipidALL Technologies Co. Ltd., China.

### Data analysis

The ChIP-seq and RNA-seq data were downloaded from NCBI GEO database (GSE82116 [67] for H3K27ac analyze; GSE71780 [29] for i-BET responding lnc-eRNAs selection; GSE141372 [68], GSE120715 [69], and GSE82116 [67] for *SEELA* locus analyze; GSE71780 [29] for ELA-enhancer analyze; GSE38339 [70] for HOXA9 binding site analyze) and analyzed by UCSC genome browser (http://genome.ucsc.edu/).

### Statistical analysis

Spearman’s correlation coefficient was used to determine the correlation between the expression levels of SEELA and SERINC2. Mann-Whitney test was used to analyze the SEELA and SERINC2 levels between patients with or without MLL fusion proteins. Fisher’s exact test was used to determine the significance of differentially expressed lnc-eRNA and mRNA levels between two groups. Data are expressed as the mean ± SEM or ± SD of three independent experiments. One-way ANOVA was performed to compare multiple groups, and the LSD *t* test was used to analyze multiple comparisons. Kaplan-Meier method with a log-rank test was used to analyze the mice survival. Two-tailed tests were used for univariate comparisons. For univariate and multivariate analysis of prognostic factors, a Cox proportional hazard regression model was used. *p* < 0.05 was considered statistically significant.

## Supplementary Information

**Additional file 1.** The selection of lnc-eRNAs.

**Additional file 2.** Figure S1-S9(PDF) with figure legends.

**Additional file 3.** Dysregulated genes in si-SEELA and si-SERINC2 group.

**Additional file 4.** The binding sites of HOXA9 in lnc-eRNAs loci.

**Additional file 5.** Clinical characteristics of all samples in the study.

**Additional file 6.** Oligonucleotides for PCR, RNA interference and sgRNAs.

**Additional file 7.** Antibodies used in the research.

**Additional file 8.** Complete western blot images of all figures in the manuscript.

**Additional file 9.** Review history.

## Data Availability

The RNA-seq data of si-NC, si-SEELA and si-SERINC2 have been submitted to the NCBI Gene Expression Omnibus (GEO; http://www.ncbi.nlm.nih.gov/geo/) under accession number GSE157040 [71]. The other ChIP-seq or RNA-seq data (GSE82116 [67], GSE71780 [29], GSE141372 [68], GSE120715 [69], GSE82116 [67], GSE71780 [29], GSE38339 [70]) used in this study are publicly available, as indicated in the “Data analysis” section.
