# Supplementary material for: Cis-acting lnc-eRNA SEELA directly binds histone H4 to promote histone recognition and leukemia progression
Source: Genome Biol. 2020 Nov 3;21:269. doi: 10.1186/s13059-020-02186-x (PMC7607629; doi:10.1186/s13059-020-02186-x)
Supplement: Supplementary file 2 — Additional file 2. Figure S1-S9(PDF) with figure legends. [file 13059_2020_2186_MOESM2_ESM.pdf]

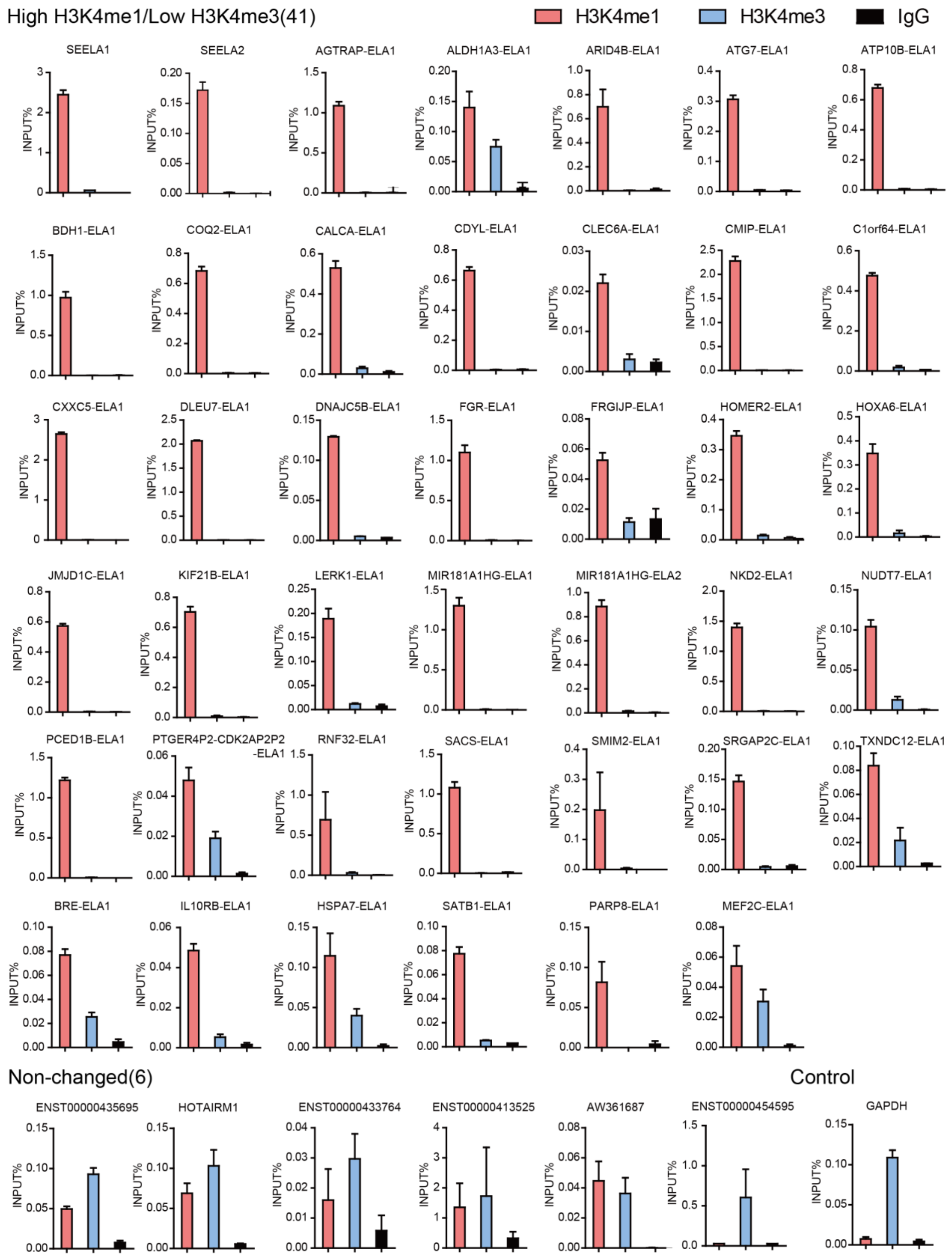

Figure S1. Identification of lnc-eRNAs.

ChIP-qPCR showed that the gene loci of 41 lnc-eRNAs exhibit a high enrichment of H3K4me1 and low enrichment of H3K4me3. GAPDH is an mRNA control. Error bars reflect ±SEM in three independent experiments. An IgG antibody was used as a negative control in the ChIP assays.

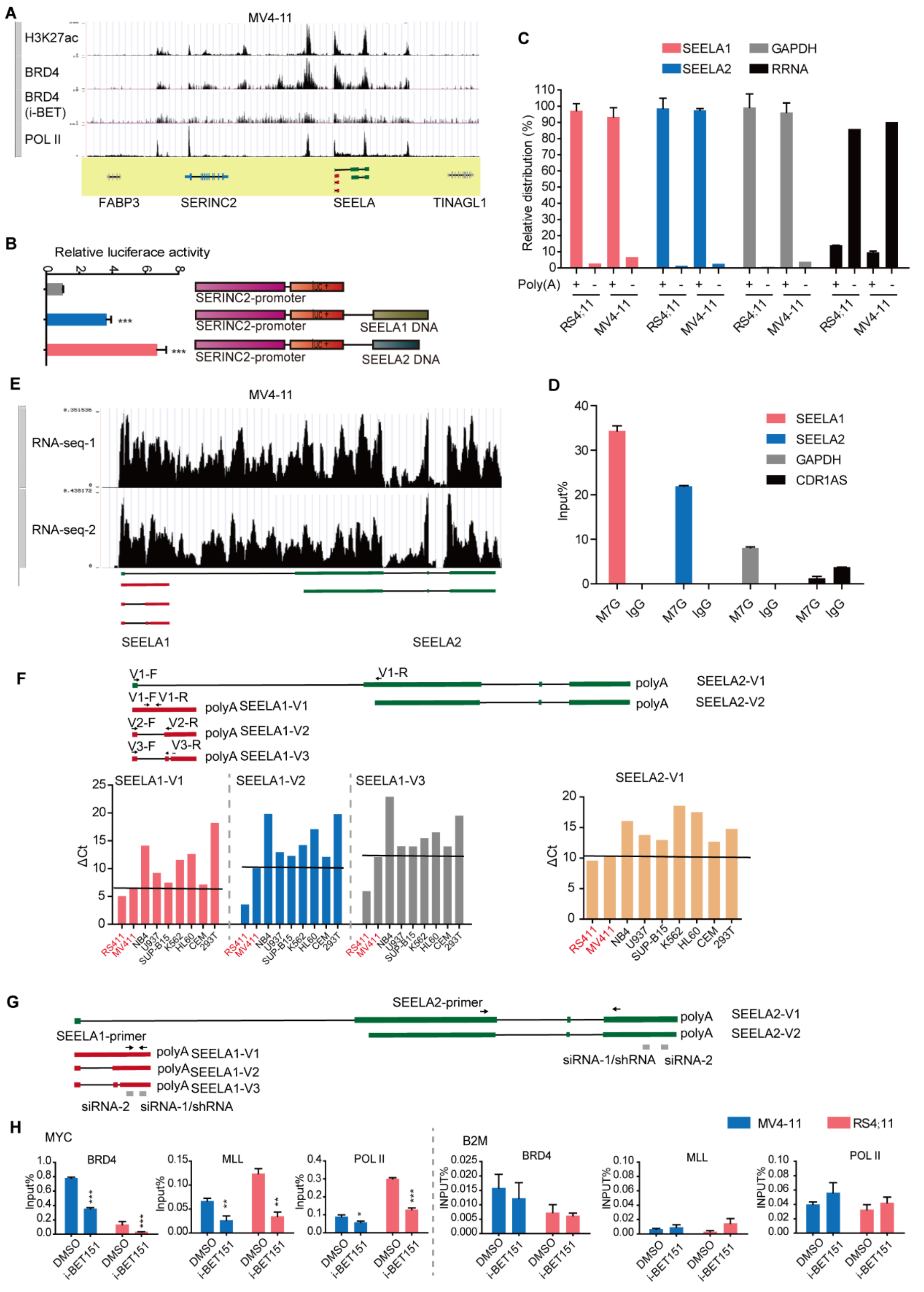

Figure S2. Identification of SEELA transcripts.

a Distribution of H3K27, BRD4, Pol II peaks across the *FABP3*, *SERINC2*, *SEELA* and *TINAGL1* gene.

b The enhancer region DNA of the *SEELA* gene body strongly induces SERINC2 promoter activity in the PGL3 reporter system. Error bars reflect ±SD (***, p<0.001) in three independent experiments (left). A schematic diagram shows the plasmids constructed for promoter activity experiments (right).

c A histogram shows the relative distribution of SEELA in MV4-11 and RS4;11 cells. Poly(A) extraction was

followed by a qPCR assay. GAPDH was the positive control, and rRNA was the negative control.

d The m7G antibody RIP-qPCR assay showed that SEELA1 and SEELA2 were m7G-capped lncRNAs. GAPDH was the positive control, and CDR1AS was the negative control.

e Genome browser view of RNA-seq data at *SEELA* locus.

f A schematic diagram shows the gene loci of five variants of SEELA (upper). The variants of SEELA were detected with different qRT-PCR primers. The results showed that the variants of SEELA were highly expressed and exhibited similar expression pattern in *MLL* leukemia cell lines MV4-11 and RS4;11 (data was analyzed by ΔCT method and normalized to GAPDH).

g A schematic diagram shows the primers used to detect the expression of all the variants and the location of siRNAs/shRNAs of SEELA.

h ChIP-qPCR detection of the enrichment of BRD4, POL II and MLL-fusion protein at the gene locus of *MYC* or *B2M* in MV4-11 and RS4;11 cells after treatment with i-BET151. Error bars reflect ±SEM (*, $P<0.05$; **, $p<0.01$; and ***, $p<0.001$) in three independent experiments.

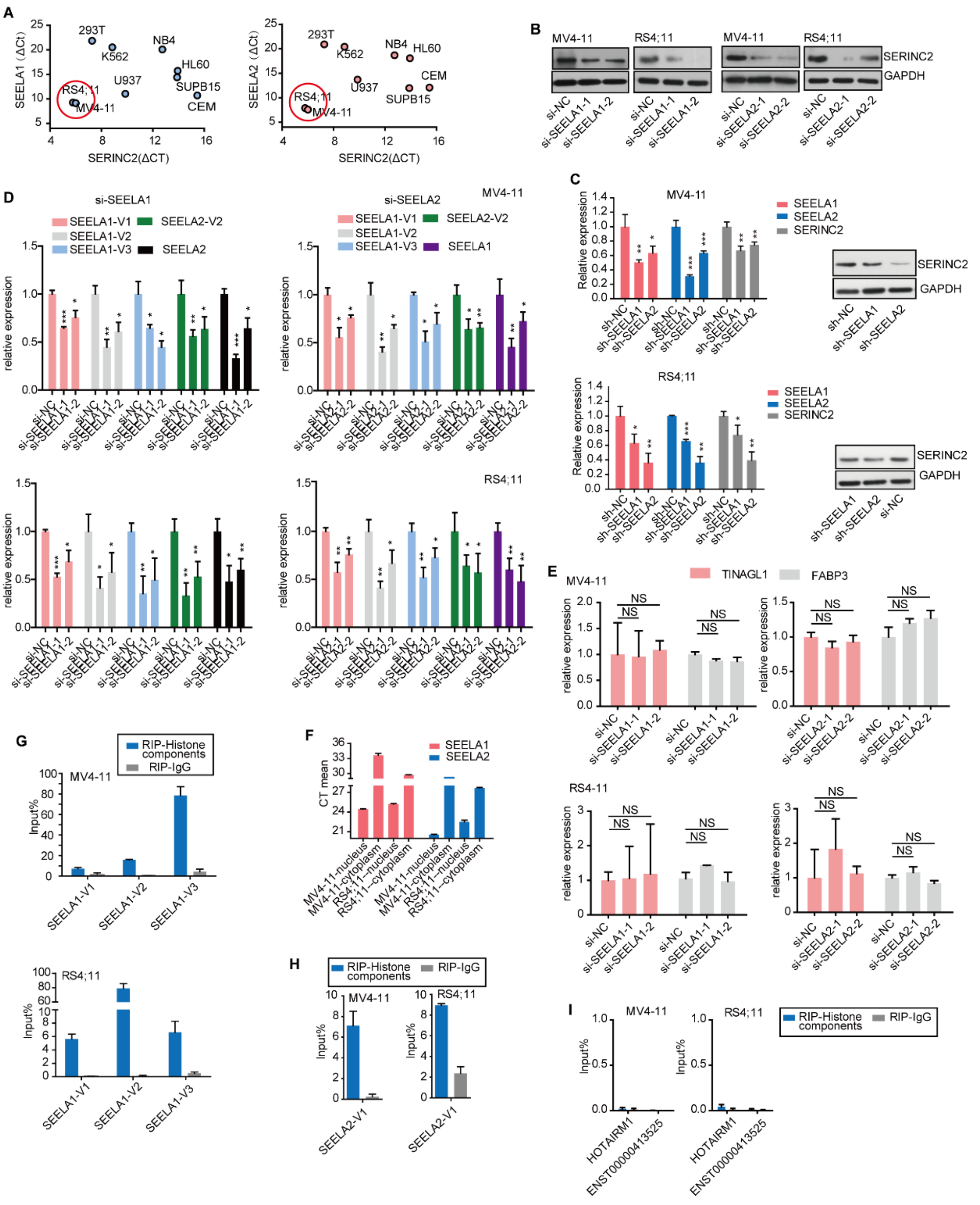

Figure S3. SEELA positively regulates nearby gene and bind histone component

a qPCR detection of ΔCT values show the expression levels of SEELA1, SEELA2 and SERINC2 in several cell lines. The y-axis scales represent the expression levels of SEELA1 (left) and SEELA2 (right), and the x-axis represents SERINC2. The red circles display the high expression levels of these genes in MV4-11 and RS4;11 cells.

b Immunoblot detection showed that the protein levels of SERINC2 were decreased after knocking down SEELA1 or SEELA2 by siRNAs in MV4-11 and RS4;11 cells.

c SERINC2 mRNA and protein were downregulated when SEELA was knocked down with shRNAs in MV4-11 amd RS4;11 cell lines. Error bars reflect ±SD (*, P<0.05; **, p<0.01; and ***, p<0.001) in three independent experiments.

d The relative expression level of SEELA1 and SEELA2 isoforms after knocking down SEELA. The error bars indicate the ±SD (*, $P<0.05$; **, $p<0.01$; ***, $p<0.001$) in three independent experiments.

e Expression levels of TINAGL1 and FABP3 in MV4-11 and RS4;11 cells transfected with si-NC or si-SEELA, respectively. Error bars reflect ±SD (ns: no significant difference) in three independent experiments.

f The CT value of qPCR detection for the nuclear and cytoplasmic expression of SEELA1 or SEELA2.

g,h RIP-qPCR detection for the enrichment of histone components of the variants of SEELA in MV4-11 and RS4;11 cells. An IgG antibody was used as a negative control in the RIP assays.

i RIP-qPCR detection showed the non-enrichment of histone components of the control lncRNAs HOTAIRM1 and ENST00000413525 in MV4-11 and RS4;11 cells. An IgG antibody was used as a negative control in the RIP assays.

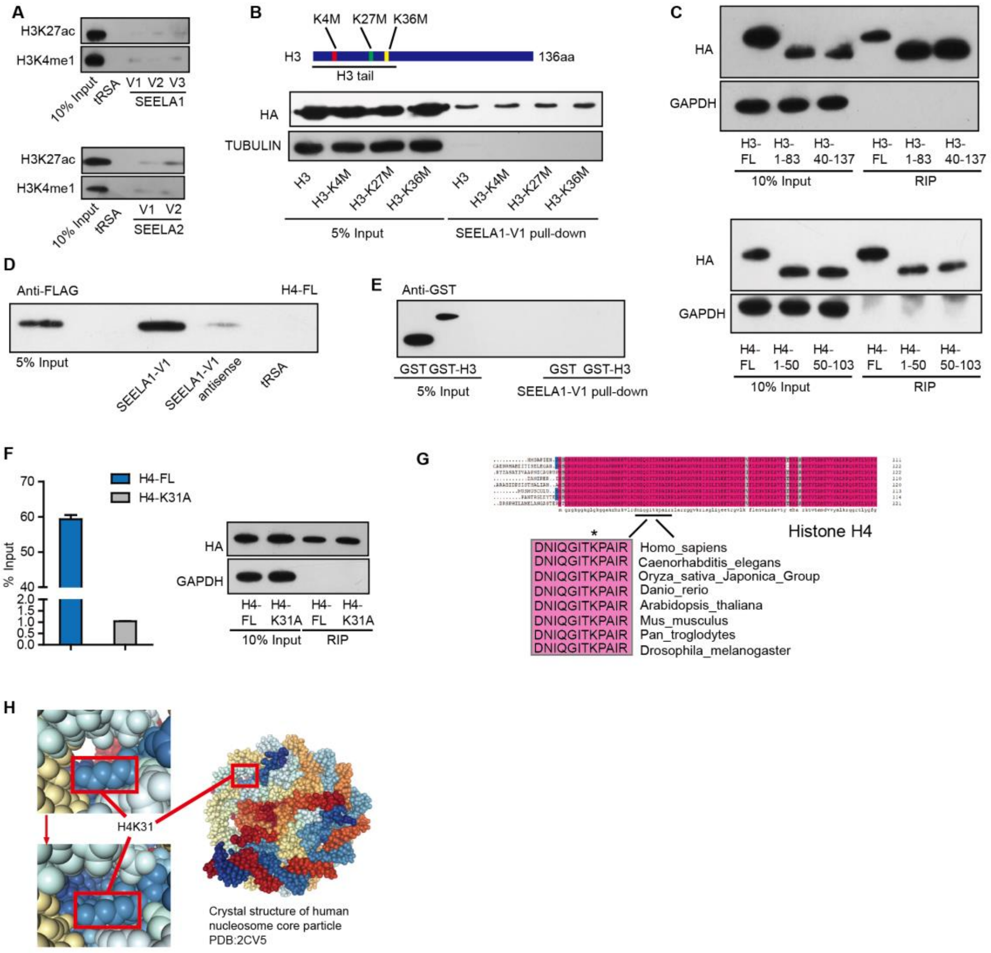

Figure S4. The interaction of SEELA with H4

a Immunoblot detection of H3K4me1 and H3K27ac retrieved by *in vitro*-transcribed tRSA-tagged SEELA variants.

b A schematic diagram shows the mutant sites of the H3 (upper). The mutants of histone H3 (K4M, K27M, and K36M) showed enrichment after SEELA1-V1 pull-down.

c The expression of truncated H3 and H4 fragment in RIP assays.

d The *in vitro* RNA pull-down assays of SEELA1-V1 with H4-FL tRSA and SEELA1-V1-antisense were used as negative control.

e The recombinant GST protein or GST-tagged H3 protein did not bind to SEELA1-V1.

f RIP-qPCR detection of the enrichment of H4-FL and H4-K31A of the SEELA1-V1. Immunoblot detection showed the enrichment of full-length and mutant H4 protein.

g K31 aa of histone H4 is conserved among various types of eukaryotes.

h The structure of human histone shows that K31 of histone H4 is located at the surface of the nucleosome core particle (PDB code: 2VC5).

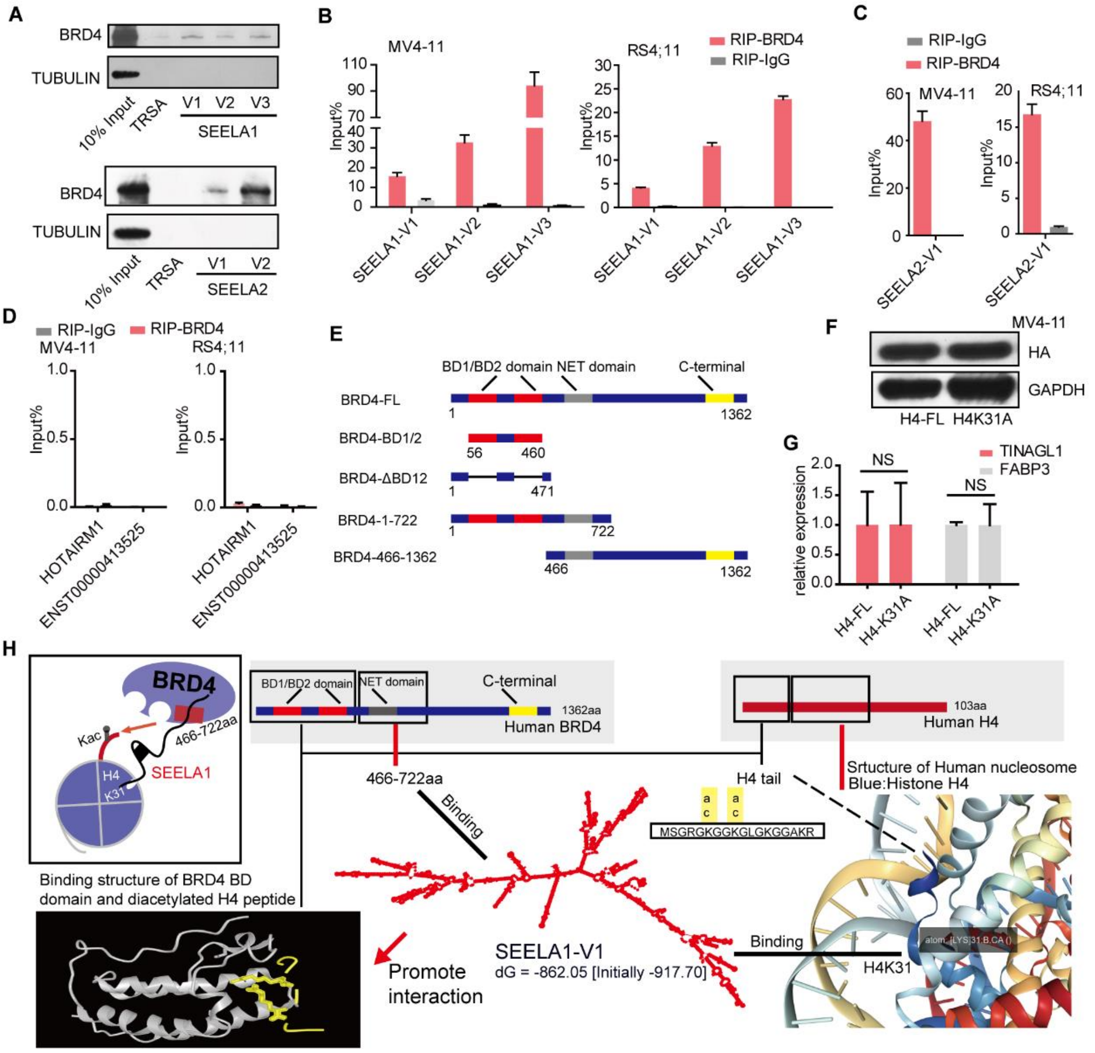

Figure S5. The interaction of SEELA with BRD4

a Immunoblot detection of BRD4 retrieved by *in vitro*-transcribed tRSA-tagged SEELA sections from MV4-11 cell lysates.

b,c RIP-qPCR detection for the enrichment of BRD4 of the variants of SEELA. An IgG antibody was used as negative control in the RIP assays.

d RIP-qPCR detection for the non-enrichment of BRD4 of the control lncRNAs HOTAIRM1 and ENST00000413525. An IgG antibody was used as a negative control in the RIP assays.

e A schematic diagram shows the functional regions and truncated fragments of BRD4.

f Immunoblot detection the expression of HA-tagged H4-FL or H4K31A in MV4-11 cells.

g The relative expression of TINAGL1 and FABP3 in H4-FL and H4-K31A cells. The error bars indicate the ±SD values (ns, no significant difference) in three independent experiments.

h A schematic diagram shows SEELA1-V1 promoted the BRD4-acetylated H4 tail interaction (left upper). The folded SEELA1-V1 (red; fold from mfold) interact with 466-722aa of BRD4 and K31aa of H4 that was presented in the structure of human nucleosome (H4 marked in blue; PDB code: 2VC5). On the left bottom is a cartoon representing the structure of BRD4 BD domain binding with diacetylated H4 peptide (BD domain marked in grey; diacetylated H4 peptide marked in yellow; PDB code: 3UVW).

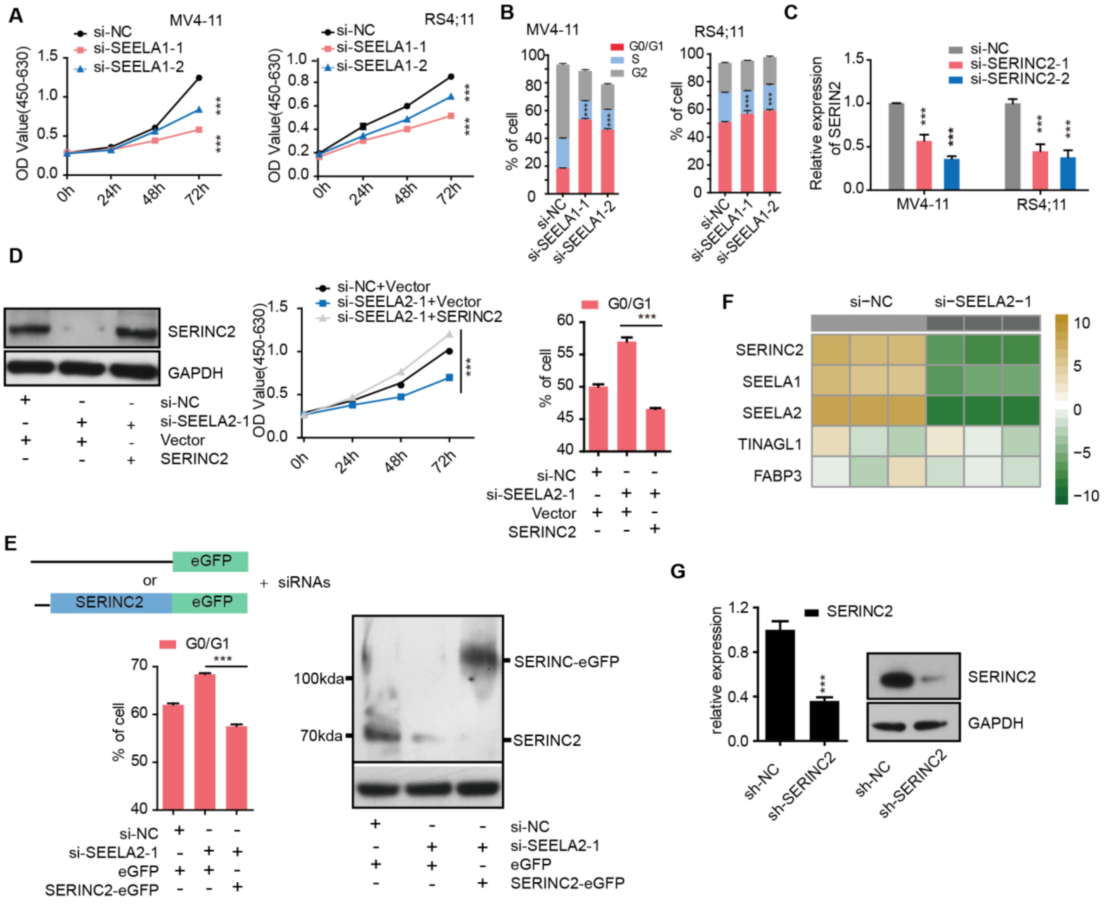

Figure S6. The regulation of SEELA-SERINC2 axis in *MLL* leukemia cells

a,b Inhibition of SEELA1 disrupted the cell proliferation and induced the accumulation of G0/G1 phase cells via CCK-8 assay at and cell cycle assay by flow cytometry in MV4-11 and RS4;11 cells. Error bars reflect ± SEM (***, p<0.001) in three independent experiments.

c qPCR analysis for SERINC2 knockdown in *MLL* leukemia cells. Error bars reflect ± SD (***, p<0.001) in three independent experiments.

d Western blot show the protein level of SERINC2 in siRNAs transfected in lentiviral vector or SERINC2 expressed MV4-11 cells. CCK8 and cell cycle assay showed the cell proliferation and cell cycle arrest effect. The error bars indicate the ±SEM values (***, p<0.001) in three independent experiments.

e Schematic outline of SERINC2 rescue strategy with eGFP tag (left upper). Western blot of SERINC2 (right) and cell cycle assay (left bottom) of transfected cells. The error bars indicate the ±SEM values (***, p<0.001) in three independent experiments.

f Heatmap of the relative expression of SERINC2, SEELA, TINAGL1 and FABP3 in RNA-seq of si-NC group or si-SEELA2 group

g qPCR and western blot assays detected the mRNA and protein level of sh-NC and sh-SERINC2 cells, respectively. The error bars indicate the ±SD values (***, p<0.001) in three independent experiments.

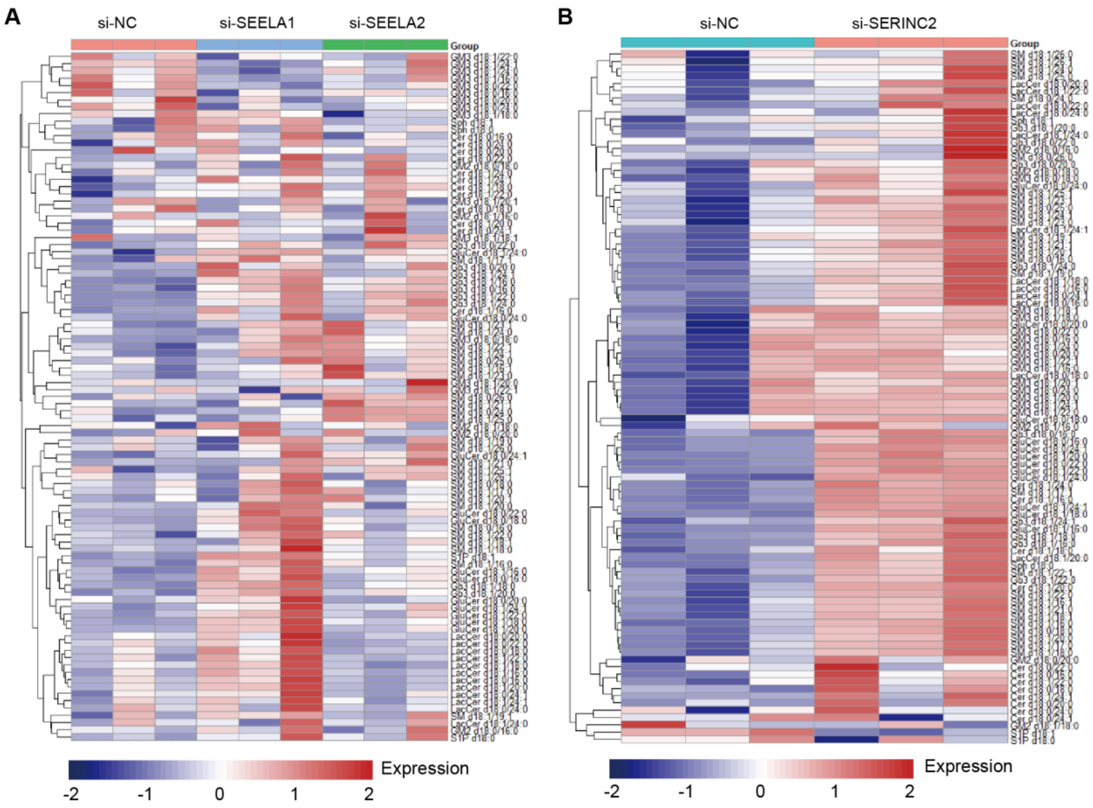

Figure S7. The concentration of different types of sphingolipids after knocking down SEELA and SERINC2.

a,b Heatmaps show the expression of sphingolipids identified by the mass spectrometry (MS) assay. Most of the sphingolipids were dysregulated after knocking down SEELA (a) and SERINC2(b).

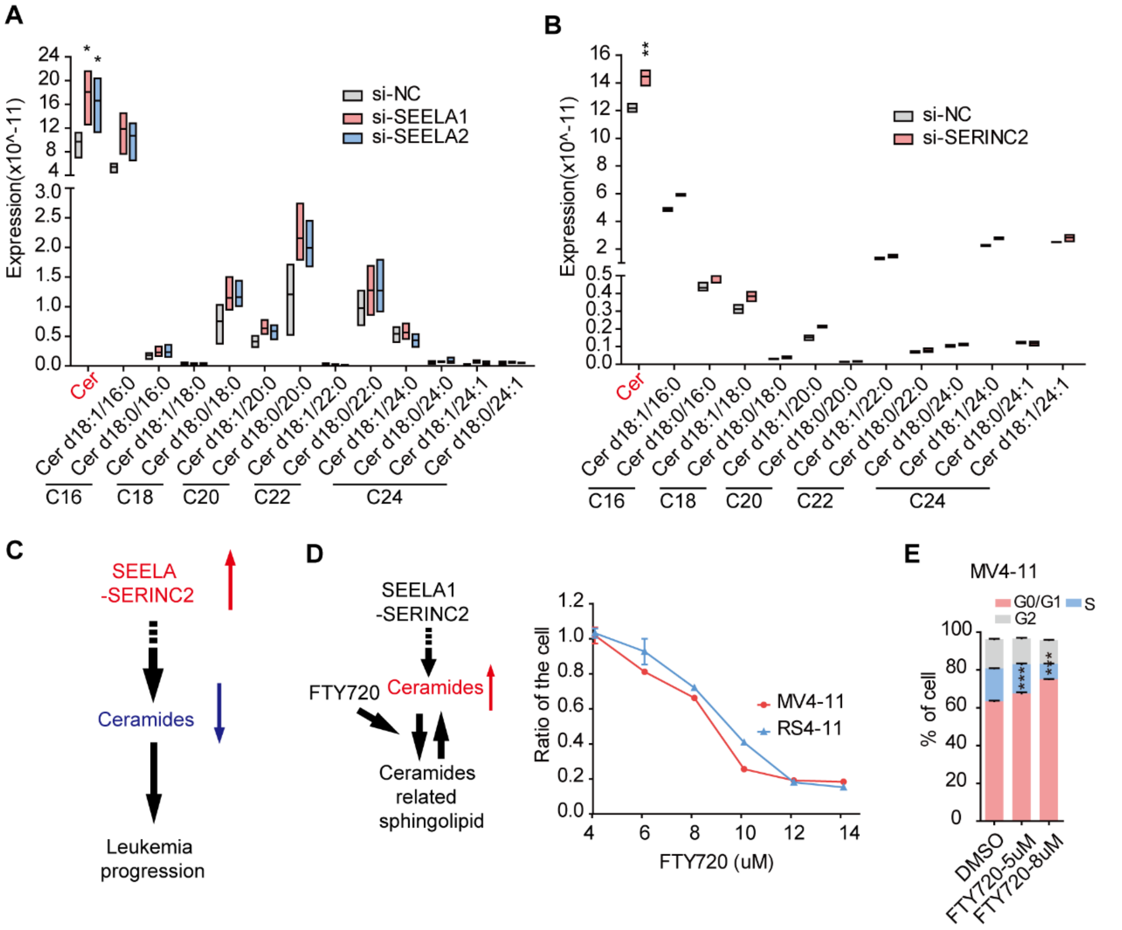

Figure S8. SEELA regulates ceramides production in sphingolipid metabolism to affect leukemia progression.

a,b MS data revealed that the total levels of ceramides were upregulated after knocking down SEELA (a) and SERINC2 (b). The levels of ceramides components C16, C18, C20, and C22 were upregulated after knocking down SEELA, and the levels of C16, C18, C20, C22, and C24 were upregulated after knocking down SERINC2, while C24 was only slightly changed. Error bars reflect ± SEM (*, P<0.05; **, p<0.01) in three independent experiments.

c A schematic diagram shows the possible role of highly expressed SEELA-SERINC2 decrease central ceramide synthesis to affect leukemia progress.

d,e The survival rate of MLL leukemia cell lines was measured using a CCK-8 kit at 24 h (d). The cells were treated with FTY720 at concentrations ranging from 4-12 μM. The treatment of FTY720 induced the accumulation of G0/G1 phase cells of MV4-11(e).

A

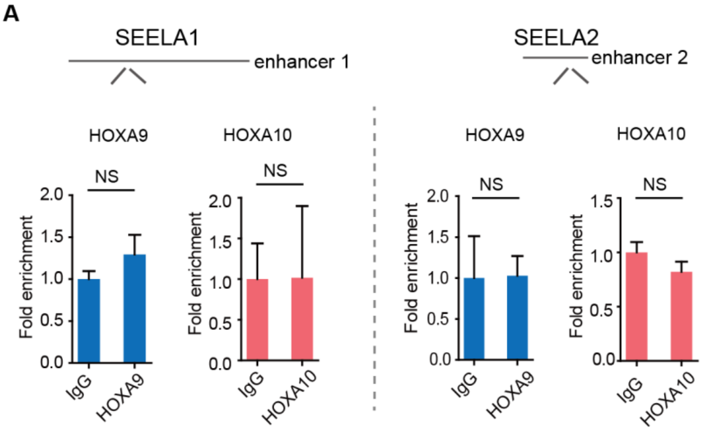

B

HOXA9 ChIP (hg 18)

chr1 Site	Fold enrichment
31728900	1.31
31728925	1.84
31728950	1.93
31728975	1.75
31729000	1.66
31729025	1.49
31729050	1.22

C

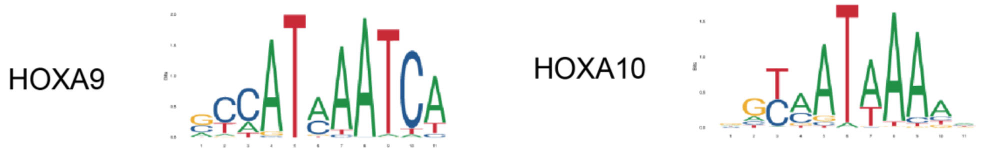

Matrix ID	Name	Score	Relative score	Sequence ID	Start	End	Strand	Predicted sequence
MA0899.1	HOXA10	10.7847	0.919792	FE1	60	70	+	GGTAATTAATT
MA0899.1	HOXA10	9.72543	0.897769	FE1	63	73	-	CGTAATTAATT
MA0594.1	HOXA9	7.44344	0.821875	FE1	61	71	+	GTAATTAATTA
MA0594.1	HOXA9	7.44344	0.821875	FE1	62	72	-	GTAATTAATTA

D

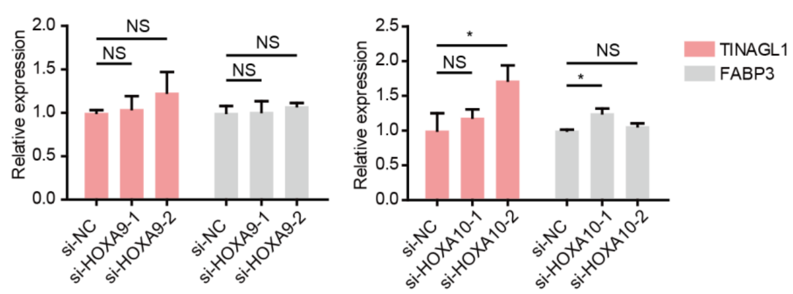

Figure S9. The binding of HOXA9/10 at the *SEELA* locus.

a Chip-qPCR of HOXA9 and HOXA10 showed that both HOXA9 and HOXA10 were not enriched at the enhancer region of *SEELA*.

b Chip-seq data of HOXA9 of *MLL* leukemia showed 150 bp HOXA9 enrichment upstream of *SEELA1*.

c The Predicted binding motif of HOXA9 and HOXA10 within ELA-enhancer 1.

d Expression level of TINAGL1 and FABP3 in MV4-11 cells transfected with si-NC or si-HOXA9/10. Error bars reflect ± SD (*, P<0.05) in three independent experiments.
