# Supplementary material for: Cis-acting lnc-eRNA SEELA directly binds histone H4 to promote histone recognition and leukemia progression
Source: Genome Biol. 2020 Nov 3;21:269. doi: 10.1186/s13059-020-02186-x (PMC7607629; doi:10.1186/s13059-020-02186-x)
Supplement: Supplementary file 4 — Additional file 4. The binding sites of HOXA9 in lnc-eRNAs loci. [file 13059_2020_2186_MOESM4_ESM.pdf]

Table S3. The binding sites of HOXA9 in lnc-eRNAs loci

PeakID	Chr	Start	End	Strand	Peak Score	Focus Ratio/Region Size	Lnc-eRNA
**chr1-142**	chr1	31956262	31956498	+	43.4	0.793	SERINC2-ELA1
**chr1-63**	chr1	31989919	31990155	+	80.2	0.912	SERINC2-ELA2
**chr8-744**	chr8	66928773	66929009	+	14.9	0.748	DNAJC5B-ELA1
**chr8-117**	chr8	66894028	66894264	+	81.3	0.754	DNAJC5B-ELA1
**chr1-405**	chr1	198906508	198906744	+	26.2	0.827	MIR181A1HG-ELA1
**chr16-119**	chr16	81464892	81465128	+	32.1	0.92	CMIP-ELA1
**chr3-790**	chr3	197228777	197229013	+	16.2	0.88	BDH1-ELA1
**chr4-122**	chr4	84161690	84161926	+	40.2	0.706	COQ2-ELA1
**chr4-346**	chr4	84152777	84153013	+	17.2	0.656	COQ2-ELA1
**chr13-208**	chr13	51252657	51252893	+	18.9	0.609	DLEU7-ELA1
**chr1-2282**	chr1	27944922	27945158	+	10.2	0.8	FGR-ELA1
**chr1-2116**	chr1	27951621	27951857	+	11.1	0.898	FGR-ELA1
**chr1-962**	chr1	27952655	27952891	+	16.6	0.875	FGR-ELA1
**chr13-482**	chr13	24034557	24034793	+	15.5	0.804	SACS-ELA1
**chr13-274**	chr13	24036585	24036821	+	20.2	0.75	SACS-ELA1
**chr5-6607**	chr5	88014589	88014825	+	7.66	0.733	MEF2C-ELA1
**chr5-2493**	chr5	88056420	88056656	+	10.2	0.764	MEF2C-ELA1
**chr5-3902**	chr5	88359959	88360195	+	8.93	0.7	MEF2C-ELA1
**chr5-5792**	chr5	88414090	88414326	+	8.3	0.83	MEF2C-ELA1
**chr5-**	chr	885896	885899	+	72.3	0.887	MEF2C-ELA1

**109**	5	70	06				
**chr5-382**	chr5	88598944	88599180	+	26.8	0.801	MEF2C-ELA1
**chr5-29**	chr5	88604391	88604627	+	150.4	0.985	MEF2C-ELA1
**chr5-187**	chr5	88692592	88692828	+	45.1	0.872	MEF2C-ELA1
**chr1-1**	chr1	121485084	121485320	+	5820	0.668	SRGAP2C-ELA1
**chr1-87**	chr1	121200604	121200840	+	62.3	0.934	SRGAP2C-ELA1
**chr1-447**	chr1	121478545	121478781	+	45.7	0.991	SRGAP2C-ELA1
**chr1-800**	chr1	121355623	121355859	+	29.6	0.875	SRGAP2C-ELA1
**chr1-694**	chr1	121357900	121358136	+	23.6	0.982	SRGAP2C-ELA1
**chr1-2871**	chr1	121386185	121386421	+	10.8	0.981	SRGAP2C-ELA1
**chr1-2345**	chr1	121258442	121258678	+	9.36	0.785	SRGAP2C-ELA1
**chr1-3086**	chr1	121225793	121226029	+	6.38	0.648	SRGAP2C-ELA1
**chr5-355**	chr5	964173	964409	+	25.3	0.672	NKD2-ELA1
**chr5-630**	chr5	976733	976969	+	25.3	0.992	NKD2-ELA1
**chr5-731**	chr5	997426	997662	+	18.9	0.823	NKD2-ELA1
**chr2-4224**	chr2	28589409	28589645	+	6.59	0.821	BRE-ELA1
**chr9-2514**	chr9	68455652	68455888	+	6.59	0.875	FRGIJP-ELA1
**chr3-1090**	chr3	11330835	11331071	+	10.8	0.617	ATG7-ELA1
**chr3-1531**	chr3	11345733	11345969	+	10	0.783	ATG7-ELA1
