# Supplementary material for: Cis-acting lnc-eRNA SEELA directly binds histone H4 to promote histone recognition and leukemia progression
Source: Genome Biol. 2020 Nov 3;21:269. doi: 10.1186/s13059-020-02186-x (PMC7607629; doi:10.1186/s13059-020-02186-x)
Supplement: Supplementary file 5 — Additional file 5. Clinical characteristics of all samples in the study. [file 13059_2020_2186_MOESM5_ESM.pdf]

Table S4. Clinical characteristics of all samples in the study

	MLL-r (N=26)		*MLL-wt* (N=75)	
	median(range)	No.(%)	median(range)	No.(%)
**Age at diagnosis, years**	3(0.33-13.0)		6(1.3-13.0)	
**Sex**				
**Male**		15(57.7)		38(50.7)
**Female**		10(38.5)		24(32.0)
**N/A**		1(3.8)		13(17.3)
**Immunophenotype**				
**B**		19(73.1)		31(41.3)
**T**		6(23.1)		17(22.7)
**N/A**		1(3.8)		27(36.0)
**WBC count, x 10^9/L**	74.00(2.75-162.00)		34.10(2.00-632.47)	
**Risk group**				
**H**		10(38.5)		22(29.3)
**M**		8(30.8)		20(26.7)
**S**		1(3.8)		5(6.7)
**N/A**		7(26.9)		28(37.3)
