# Supplementary material for: Cis-acting lnc-eRNA SEELA directly binds histone H4 to promote histone recognition and leukemia progression
Source: Genome Biol. 2020 Nov 3;21:269. doi: 10.1186/s13059-020-02186-x (PMC7607629; doi:10.1186/s13059-020-02186-x)
Supplement: Supplementary file 6 — Additional file 6. Oligonucleotides for PCR, RNA interference and sgRNAs. [file 13059_2020_2186_MOESM6_ESM.pdf]

Table S5. Oligonucleotides for PCR, RNA interference and sgRNAs

Primers for quantifying target genes		
**Name**	**Forward Primer**	**Reverse Primer**
qSEELA1	GAGCGTGGTGTCAAGGTCATC	AGGCGGAAGGAGCCATAGTG
qSEELA2	CCAGCCTACATCTTTCTCCCAT	CTTCCAATCTGAACGACCTCATC
qGAPDH	GAGAGACCCTCACTGCTG	GATGGTACATGACAAGGTGC
qSERINC2	GAGCCGCCTCATCTTCACG	TAGCCAAGCAGGGAGCCAC
qSEELA1-V1	TGGGGCTGGTCCTTTATTCA	GCCTCCCTTTGCTTCTCATTC
qSEELA1-V2	CACAGGGCTGGGCATGATAT	GAGGGACAGCAGGAAGAGGTTA
qSEELA1-V3	CACAGGGCTGGGCATGATAT	CTGATCACTCTCGCCAACTGC
qSEELA2-V1	CACAGGGCTGGGCATGATAT	ACACCGCATTCCTTGGACAC
qBRD4	ACCTCCAACCCTAACAAGCC	TTTCCATAGTGTCTTGAGCACC
qHOXA9	GAGGCAGGTCAAGATCTGGTTCC	GATGTGGCCTGAGGTTTAGAGC
qHOXA10	CTCACGGACAGACAAGTGAAAATC	CAGCCCTGCACAGATGTAACG
qAGTRAP-ELA1	GACAATGACCCATCACATACTCTG	GCAAAGCCACTTACTCCTCCA
qALDH13-ELA1	TCCCTAGCCGTCAACCCTA	CCGTGCCCTAATTCGTCAG
qATG7-ELA1	CCTTCAAGGTTGTGCGTCTG	TAGCTGTCACTGCCTGGTACTTC
qATP10B-ELA1	CTTCACGGGATCAGAGGGA	TACAAAGGGACAATGTAGCCC
qBDH1-ELA1	GCAGGCTGGTTGCTCTTCTCA	TCCACCTCTGGTCGGACATTA
qBRE-ELA1	TCTCAGCGGAAGGATTGGA	CCGGCTACGCTATTTACGAG
qC1orf64-ELA1	GAATGTTTGGACCTACTTACCCTC	GCTTTATCTCCTTTAGCATCCC
qCDYL-ELA1	CGGGTCCACAGAGTTCCAGAG	CCTTCAACAAGCGTTTAGCAG
qCMIP-ELA1	AGTGGCAAGGTTGGTGAGTATCT	CGATCTGCCTGCTTGGGAC
qCOQ2-ELA1	AAGGGACTGAGCTGAGCTTGTAA	ATAATGGAGTGGCAGGGTCTTG
qCXXC5-ELA1	CTGAAATCCCCTGGACTGC	CTGCTAATGAACCAAACTTGCT

qDLEU7-ELA1	CCAGGGCAATGTCACGACT	CAATGGCAAAAGGACGGA
qDNAJC5B-ELA1	CCTGAAACACCATCCAGACAA	GTAGAGTCCCAGCGATCCGTA
qFGR-ELA1	GCAGCAGCCTGTATCTCACC	TCACTCCTCTTCCAGCCATTT
qFRGIJ-ELA1	CCTGGAGTGCTCATAACGTGG	CATTTCTTGAATCTGTGCTGGTC
qJMJD1C-ELA1	CCTGAGCCAAGGAGTTCGAAGT	GATGCAAATGACCTACAAAGCCC
qKIF21B-ELA1	GTTTCCACAGCAGCACAATAGA	GTCTCAAACTCCTGGCCTCAA
qLERK1-ELA1	AACTTGAGGAGACAATAGTGACGAA	AGGTGCCGAAATGTTATAGTTAGG
qMEF2C-ELA1	GGTTCCTTCCTTGGAGTGTTTCT	GCACTTTGTTTATACCTTAGGATCCG
qMIR181A1HG-ELA1	TGTTGTAGGCTGCATGTTGG	AGCTTAGCTGAGTCCTCTGTTTG
qMIR181A1HG-ELA2	TTCAGTTTTGGGTCTCAGCAC	ATGGCTGTGGGATGTGAAGA
qNKD2-ELA1	AGCCGCCCACTGACTCCTA	CGCCAACTGCCTTCCTTCT
qNUDT7-ELA1	GTGAACAACATCCCTGTCGCTT	AAATCATCCACCAAAGCACG
qRNF32-ELA1	CGGAGTGAATTGAGGCAACG	GAGTCTTCCCGAGCAGGTCT
qSACS-ELA1	TCCCGATAGCCAAGCAGAA	TAAACCGCCGAAGGACGA
qSRGAP2C-ELA1	TTGGACCTGGTACAGTAGTAATGG	CGTCTAATTGGAATAGTGCATG
qTXND12-ELA1	GCTCCACGCAATGCCTTCT	CCAATGGTCGCTCCCTGA
qENST00000413525	GACCCGCAGGCTGTGGAA	CAAAAGCCCTGAAGCCGAG
qHOTAIRM1	AGGGGGTTGAAATGTGGGTG	CTTGAAAGTGGAGAAATAAAGTGCC
**Primers for Chip**		
SATB1-ELA1	CTGTTCAGAGGAGAAACCCTTCAC	GTCTTAAGTCACATTCAGTCCCATGA
SMIM2-ELA1	GTTTGGCATCGCAGTTCCG	GTGGCTATCTTTTCCCCATTTC
HOTAIRM1	GGAGACGAGGGCAAGAAAAGA	GGTGCAAAACTGAGTGGGAAA
HOXA6-ELA1	AGCCGTCCTTCTTTGCCATAG	GCCGTTATAGACTCGGGTCCT
CALCA-ELA1	CTCACTGTGCCTTCGGACCTAC	CAGCCATCTTCTCGCTAACTCG

PCED1B-ELA1	CAAGAACAATAAGAGCCAAGGGTC	CGGAAGACTAATGACCGAAGC
IL10RB-ELA1	TTTTGCGATAACCTCTATGGCTG	TGTCCAGTGTCTTGGGTAATGC
AW361687	GGATTCAGTTCCGCCGACA	CCCTAGTGGGAGACCGAGATT
ARID4B-ELA1	AGGCAGGGGAATCGCTTGA	CTGTCCTTAGGTGGGTGACG
ENST00000433764	ACCTCACCCTCCGTGCGTT	CCGTTCCGATTTTCCCCTGAG
ENST00000454595	CAACCAGTTTGCCTGAAAGGTG	CAGCATCTAAGTGAGGATCATAAACAC
PTGER4P2-CDK2AP2P2-ELA1	GAAAACCAATGGAGCCCTGAG	TGCCACCGACTTCGTGTAAA
CLEC6A-ELA1	GGTGTAAGTGTTGTATGCTCCCAG	GTCCACGAATGTTGAAGCCCTA
HOMER2-ELA1	GTTTCTCCCACTTCCCCTTTTG	GCAGGAAGGTGGCTTTGGT
HSPA7-ELA1	ATGAAAAGCCCGTGGAAGC	GAGTAGGTGGTGCCCAGGTC
PARP8-ELA1	GACCTGAACCACCCTCCCACT	CATCACCTGCTGTCTGTCTCCA
BRE-ELA1	TCTCAGCGGAAGGATTGGA	CCGGCTACGCTATTTACGAG
GAPDH:	CCCAACTTTCCCGCCTCT	CCTTACACGCTTGGATGAAACA
B2M	CGGGCTCTGCTTCCCTTAGACT	TGCTAGGACATGCGAACTTAGC
ENST00000413525	CTCCACTGACAGGAACCAGCAA	ATCAAATGCAGGGACCGAAG
COQ2-ELA1	CGTGAAGATGACAAAAGAAGAATGAC	GTGCCTTAGAAAGTAAGAAGTAGTGACTGA
LERK1-ELA1	AGCCCGCTGTCCCCTTATC	CCCTCCACCGACTTTAGTTCG
CDYL-ELA1	TTCCTGTTGGGAGGCAAGTGT	GAAGCAGGGCACAAAGATGAGT
AGTRAP-ELA1	GCAGATTCCAGGCACCGAG	CCACTCTTGGTGTCTCCAGACAA
NKD2-ELA1	CTATTGAAGGCATTTTGAGGAGG	GGCTACTCCCAAGACAGGAAAA
FRGIJP-ELA1	GTAGGCTTTTGGCTTCCCG	AGGAGGACCTGGGCTTCTG
SACS-ELA1	GCCCTCACGGAACCCTAAAA	CTCCTTGGATGCAAAACAATG
FGR-ELA1	AGCCTATGCTCCCTCATCTTCC	CCTTCAAGTCCTCAGTCCTACAA

KIF21B-ELA1	CCCACGCTACTGTAGTTAAAATAATCC	CCCAGTGCTAACCTGAAGAAGATAA
ALDH1A3-ELA1	CCACTCAGGAGGGAGCCAAT	GACAGAGCGAGACTCCACCTAAA
ATP10B-ELA1	TGCTATGGATGATTGCTGTGG	CAGGCGGAACACTGAGAAATCT
C1orf64-ELA1	CAATCTCCACTCACCACAACCTC	CTGTTTCTGGTGTTCCCAAGTC
TXNDC12-ELA1	AAACACCACGAGCGGCATC	CAAGAGGGTCTGTAAAAGTCAAGG
MIR181A1HG-ELA1	GAGGCGAAATGATTGACTTAGG	GATAGTATTGAGTGAGAATAACCGAGG
DLEU7-ELA1	ACAGACGGGCATTTGGAAATC	GTATGACAGCTCTGCATGAACCAC
DNAJC5B-ELA1	CTGGAAAAGCCGCAGACACT	GATGAAAAGGTAGGTTCCCATAGTC
ENST00000435695	CTTCAACATTTCCGCATTCC	CTTGTCGCTGTGCTGGTGG
CMIP-ELA1	AAGACAGGCTTTGTGCTGAGGA	CTTAACCACCATGCCACATTGTC
SRGAP2C-ELA1	GTCCCTAATCCCAAGCCAGAAG	CTGGATGGACGCTGAAATACAAC
MEF2C-ELA1	CACGAATGGTTGGGGTTCTAAG	ACTGGAGGGGTGAGGAGGGA
RNF32-ELA1	AACCCGCAACGAGAAGCAA	TTTCCCTGCGGTGGCTG
NUDT7-ELA1	CAGAGTTGGACCCCAGGAC	CAGTAGCAGCCACCCCGTT
MIR181A1HG-ELA2	GTATGATGGCACCCTCCTGTAGTC	CCTCGTCATCTGTAAGTTGGGAA
CXXC5-ELA1	CCCGACAGCAGCCAGTCTT	CCAGTACAGCCACAGGACCTATC

JMJD1C-ELA1	AGCCTGGATGACAGAGCAAAAC	GCATATTTTCCCCTCTTCCTTGTC
SEELA2	AACCTGGGTAGTGGGAGCCCTATC	AGCAAGGGTTGTGAAATCGCATC
SEELA1	GGTTCTGGTCCTTCCCTTGCT	CCCAGTGGTTTCGTGCCTTA
SERINC2-promoter	CTTCTGTTCTGCTCCGAGTAGTTTC	GGTGCCGAGTGAAGGTCCA
ELA-enhancer1-1	CTGTTAATGTCCATGCTGCTTTCTAG	GCTACCCACATTTTCTTGCTTTTG
ELA-enhancer1-2	TGCTGAAATGTCTTTCTGCTGG	TAAACCAAAGGGAGTTGACGC
ELA-enhancer2	CTGGTTTTCCTTGCGGCAC	ATAGCTGTTGTCCATGTCCCTG
ELA-enhancer3	GGGTTCTGAGTTAGAGCCAGGGT	ATGGGGAGTGATTGCTTCATTG
MYC	GAGCAGCAGAGAAAGGGAGA	CAGCCGAGCACTCTAGCTCT
ATG7-ELA1	CCTTCAAGGTTGTGCGTCTG	TAGCTGTCACTGCCTGGTACTTC
BDH1-ELA1	GCAGGCTGGTTGCTCTTCTCA	TCCACCTCTGGTCGGACATTA

The primer used in full length amplification

SEELA1	ACACAAGCTATCGCTGACTGCCGGTGCATGTTGG	TGAAATATGTCTAGATTTTTTAATATGGGC
SEELA2-V1	CTAGCTAGCAAGCTATCGCTGACTGCCGGTGCATG	ATAAGAATGCGGCCGCaactgtgtcatgcatcctttaaatttg
SEELA2-V2	CTAGCTAGCAATTTTGCATTCTGTCCCTTAAATCATAAAG	ATAAGAATGCGGCCGCaactgtgtcatgcatcctttaaatttg
SERINC2	CGGAATTCATGGGGGCCTGCCTGGGAG	ATTTGCGGCCGCATGGGAGCGGAGGGAGC
H3	CGGAATTCATGTACCCTTATGACGTACCTGACTATGCTGGA GCCCGAACCAAGCAGACT	CGGGATCCTTAAGCTCTCTCTCCCCGTA
H4	CGGAATTCATGTACCCTTATGACGTACCTGACTATGCTGGATCTGGTCGCGGCAAAGGC	CGGGATCCTCAGCCGCCGAAGCCATAC
HOXA9	ATGCACCATCATCACCACCATGCCACCACTGGGGCCCTGGG	TCACTCGTCTTTTGCTCGGTC

The sequences of siRNAs and sh-RNAs

Name	Sense sequence(5'-3')	Antisense sequence(5'-3')
si-SERINC2-ELA1-1	CCACACUGCUUUGUAAAUATT	UAUUUACAAAGCAGUGUGGTT
si-SERINC2-ELA1-2	GCGAUCACCAGAACACAAATT	UUUGUGUUCUGGUGAUCGCTT
si-SERINC2-ELA2-1	GGGCAAUAAUCUCCUUUCUTT	AGAAAGGAGAUUAUUGCCCTT

si-SERINC2-ELA2-2	GCCUGAGCUAACCUGGGUATT	UACCCAGGUUAGCUCAGGCTT
si-SERINC2-1	GGTCAGCCCTATCCAGTATTT	ATACTGGATAGGGCTGACCTT
si-SERINC2-2	TGACGCTCACCAACTGGTA	TACCAGTTGGTGAGCGTCA
si-HOXA10-1	GCCAAAUUAUCCCACAACATT	UGUUGUGGGAUAAUUUGGCTT
si-HOXA10-2	GCAAAGAGUGGUCGGAAGATT	UCUUCCGACCACUCUUUGCTT
sh-SERINC2-ELA1	GATCCCCACACTGCTTTGTAAATATTCTTCCTGTCAGAAATATTTACAAAGCAGTGTGGTTTTTG	AATTCAAAAACCACACTGCTTTGTAAATATTTCTGACAGGAAGAATATTTACAAAGCAGTGTGG G
sh-SERINC2-ELA2	GATCCGGGCAATAATCTCCTTTCTTTCTTCCTGTCAGAAAAGAAAGGAGATTATTGCCC TTTTTG	AATTCAAAAAGGGCAATAATCTCCTTTCTTTTCTGACAGGAAGAAAGAAAGGAGATTATTGCCC G
Sh-SERINC2	GATCCGGTCAGCCCTATCCAGTATCTTCCTGTCAGAATACTGGATAGGGCTGACCTTTTTG	AATTCAAAAAGGTCAGCCCTATCCAGTATTCTGACAGGAAGATACTGGATAGGGCTGACC G
si-HOXA9-1	CCGGCCUUAUGGCAUUAAATT	UUUAAUGCCAUAAGGCCGGTT
si-HOXA9-2	CCAAUAACCCAGCAGCCAATT	UUGGCUGCUGGGUUAUUGGTT
**The sgRNA primers**		
SEELA1-VPR-sg-1	CACCGCAGGGTGAGTGCCCCTTCGT	AAACACGAAGGGGCACTCACCCTGC
SEELA1-VPR-sg-2	CACCGGCGGTTATCCACAGTCTGTG	AAACCACAGACTGTGGATAACCGCC
SEELA1-VPR-sg-3	CACCGGGCCAACGAGGCACCTACGA	AAACTCGTAGGTGCCTCGTTGGCCC
SEELA2-VPR-sg-1	CACCGGCAGCTGGTCACATGGTCAT	AAACATGACCATGTGACCAGCTGCC
SEELA2-VPR-sg-2	CACCGACTCAATAAAAACTTGCACT	AAACAGTGCAAGTTTTTATTGAGTC
SEELA2-VPR-sg-3	CACCGGCTGGTCACATGGTCATAGG	AAACCCTATGACCATGTGACCAGCC
