# Supplementary material for: Cis-acting lnc-eRNA SEELA directly binds histone H4 to promote histone recognition and leukemia progression
Source: Genome Biol. 2020 Nov 3;21:269. doi: 10.1186/s13059-020-02186-x (PMC7607629; doi:10.1186/s13059-020-02186-x)
Supplement: Supplementary file 7 — Additional file 7. Antibodies used in the research. [file 13059_2020_2186_MOESM7_ESM.pdf]

Table S6. Antibodies used in the research

Antibodies	Source	Identifier
**Anti-SERINC2**	Novus Biologicals	NBP1-60102, RRID:AB_11009118
**Anti-FLAG**	Sigma	F7425, RRID:AB_439687
**Anti-HA**	Cell Signaling Technology	#3724, RRID:AB_1549585
**Anti-GST**	Cell Signaling Technology	#2624, RRID:AB_2189875
**Anti-IgG**	Cell Signaling Technology	#2729, RRID:AB_1031062
**Anti-IgG**	Sigma	M8642, RRID:AB_260698
**Anti-GAPDH**	Proteintech	10494-1-AP, RRID:AB_2263076
**Anti-β-tubulin**	Invitrogen	32-2600, RRID:AB_86547
**Anti-H3**	Abcam	ab1791 , RRID:AB_302613
**Anti-H4**	Abcam	ab7311 , RRID:AB_305837
**Anti-H3K27ac**	Abcam	ab4729 , RRID:AB_2118291)
**Anti-H3K4me1**	Cell Signaling Technology	#5326, RRID:AB_10695148
**Anti-H3K4me3**	Cell Signaling Technology	#9751, RRID:AB_2616028
**Anti-HOXA9**	Proteintech	18501-1-AP
**Anti-HOXA10**	Santa Cruz Biotechnolog	sc-271428 , RRID:AB_10649855
**Anti-BRD4**	Cell Signaling Technology	13440, RRID:AB_2687578
**Anti-POL II**	Millipore	05-623 , RRID:AB_309852
**Anti-MLL1**	Bethyl	A300-086A , RRID:AB_242510
**Anti-m7G**	MBL	RN017M, RRID:AB_2725740
