# Supplementary figures and images for: Cis-acting lnc-eRNA SEELA directly binds histone H4 to promote histone recognition and leukemia progression
Source: Genome Biol. 2020 Nov 3;21:269. doi: 10.1186/s13059-020-02186-x (PMC7607629; doi:10.1186/s13059-020-02186-x)

Complete western blot images of all figures

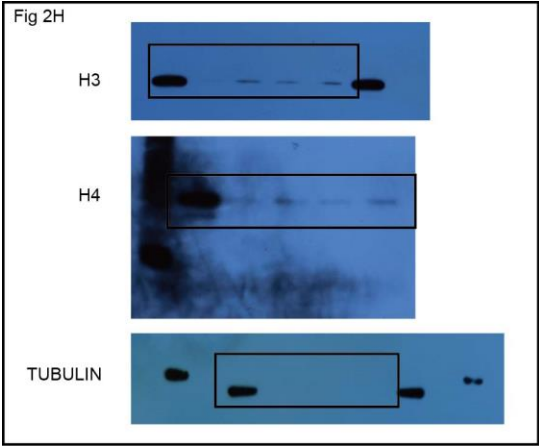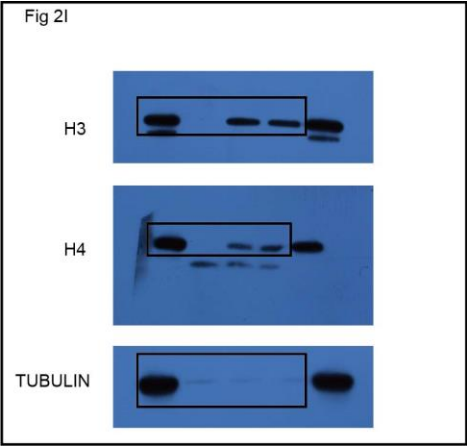

Fig 3C

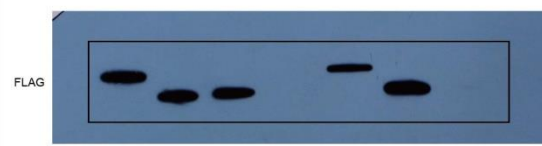

Fig 3D

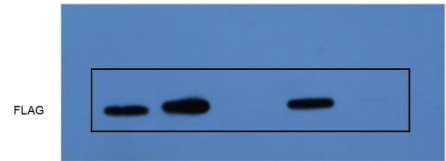

Fig 3E

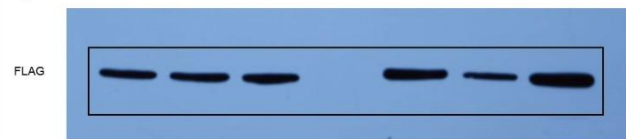

Fig 3G

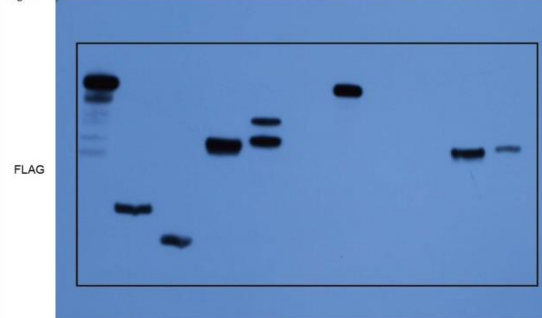

Fig 4E

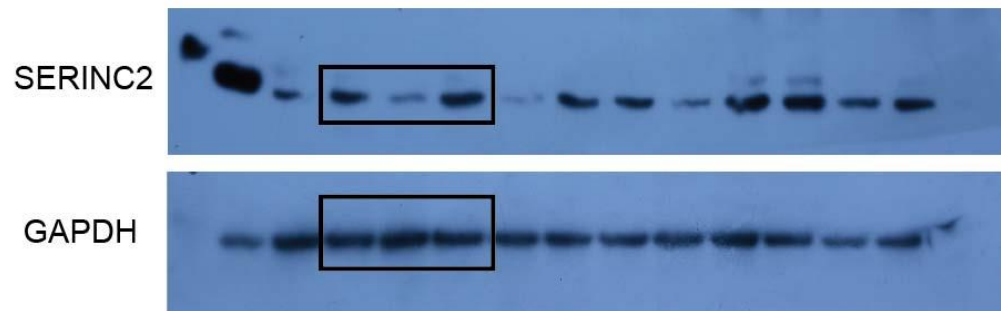

Fig 4F

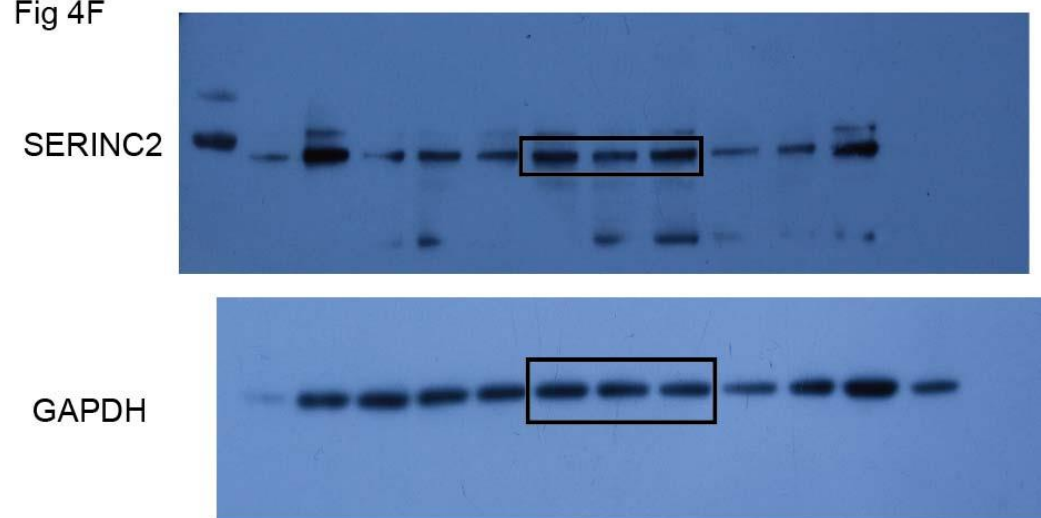

Fig 5E

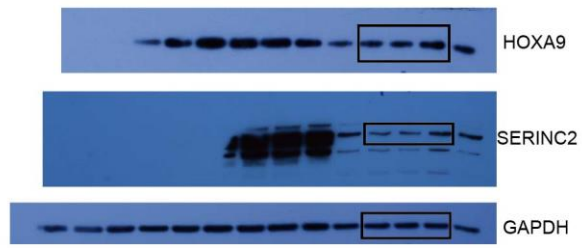

Fig 5F

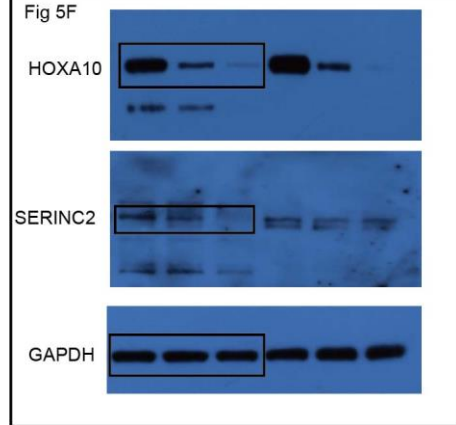

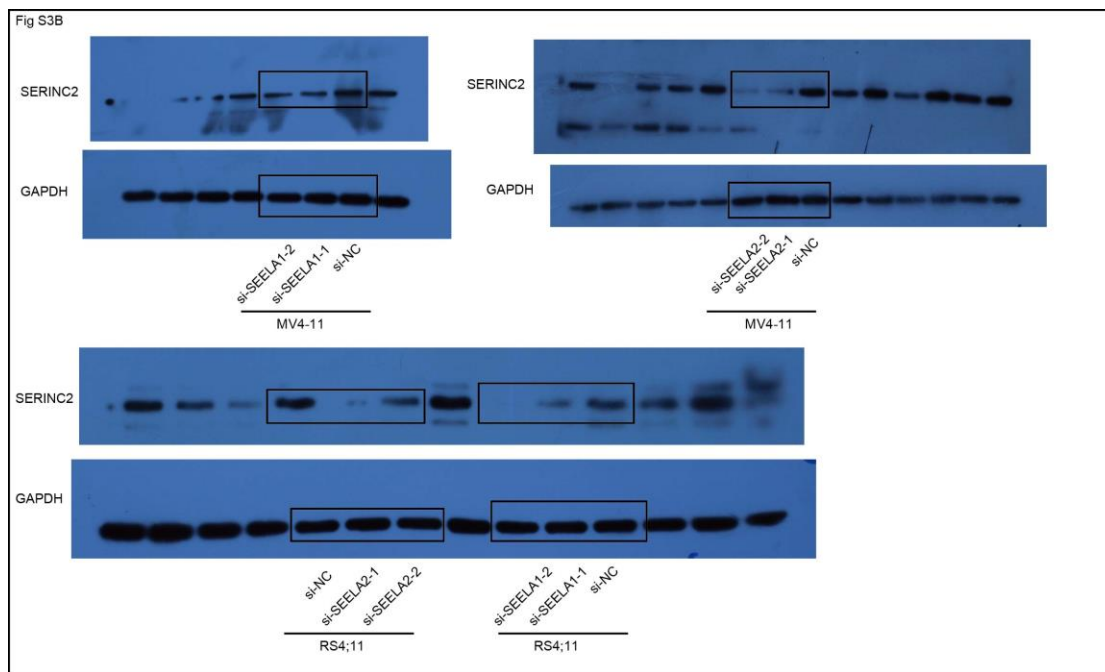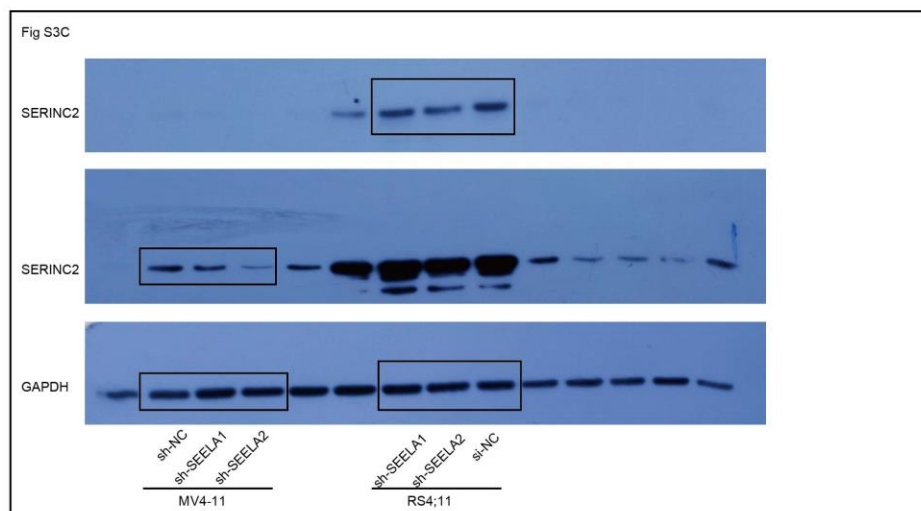

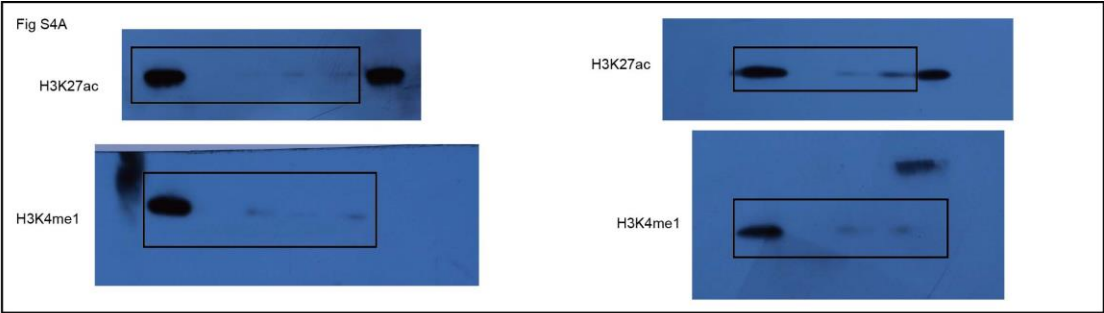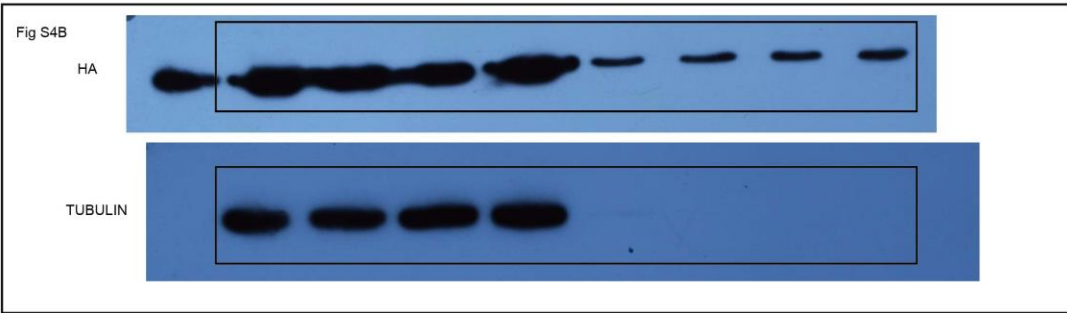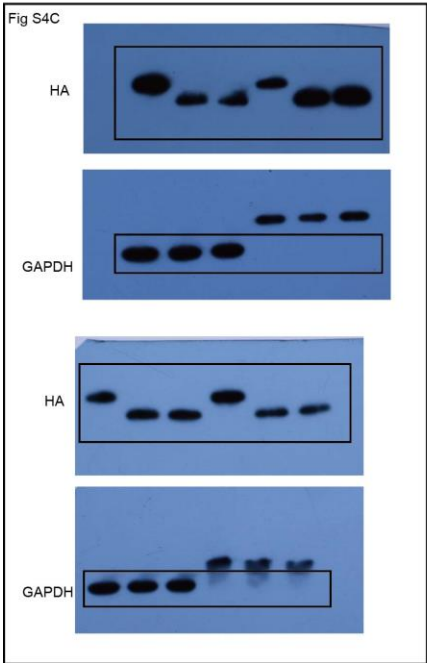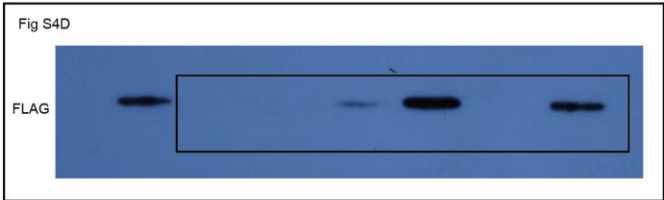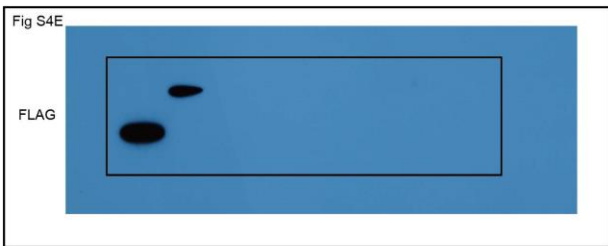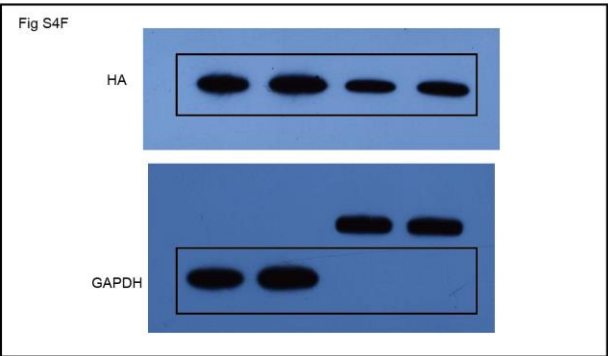

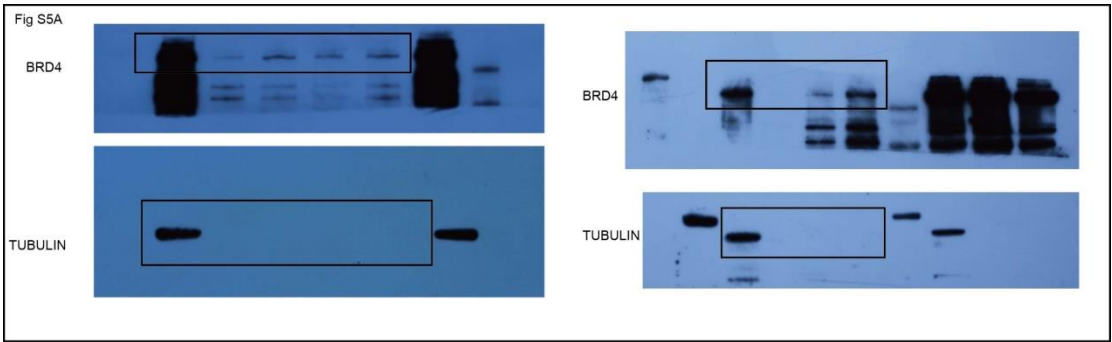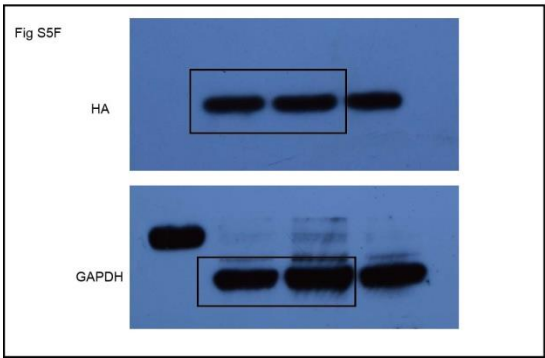

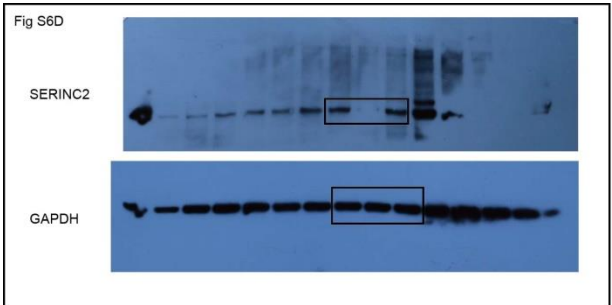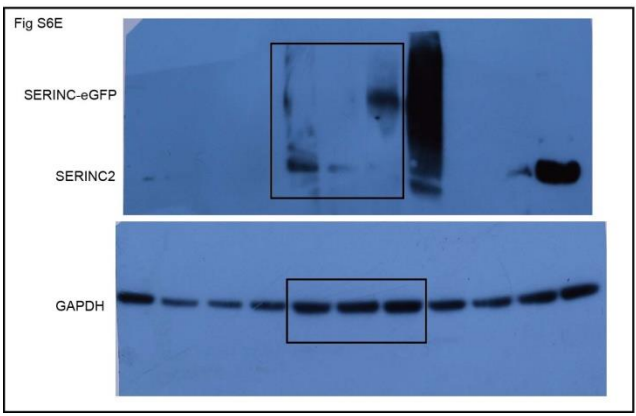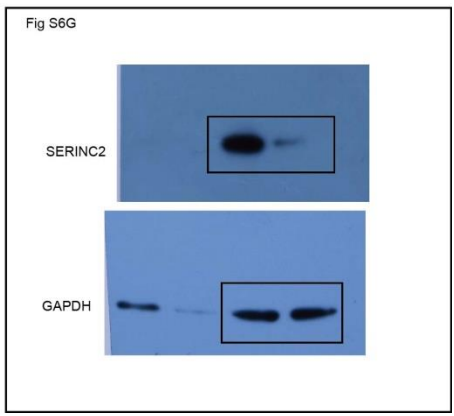

Supplement: Supplementary file 8 — Additional file 8. Complete western blot images of all figures in the manuscript. [file 13059_2020_2186_MOESM8_ESM.pdf]
