# Supplementary material for: Cis-acting lnc-eRNA SEELA directly binds histone H4 to promote histone recognition and leukemia progression
Source: Genome Biol. 2020 Nov 3;21:269. doi: 10.1186/s13059-020-02186-x (PMC7607629; doi:10.1186/s13059-020-02186-x)
Supplement: Supplementary file 9 — Additional file 9. Review history. [file 13059_2020_2186_MOESM9_ESM.docx]

Review History

**First round of review**

**Reviewer 1**

**Are you able to assess all statistics in the manuscript, including the appropriateness of statistical tests used?** Yes, and I have assessed the statistics in my report.

**Comments to author:**

In the manuscript by Fang et al., entitled:" Cis-acting lnc-eRNA SERINC2-1 ELA1/2 directly binds H4 to promote histone recognition and leukemia progression", the authors aim to identify and functionally characterize lncRNAs generated from enhancers (i.e. lnc-eRNAs) that are deregulated in hematologic malignancies, and to provide potential new therapeutic targets. The authors used previously published microarray data to identify deregulated lncRNAs in cancer that overlap enhancer H3K27ac mark, which resulted in tens of putative lnc-eRNAs. They used cell lines to validated some of these lnc-eRNAs and identified two candidates "SERINC2-1 ELA1/2" due to their sensitivity to BETi / BRD4 inhibition, and their overexpression in MLL mutant cancers, and found that they potentially act via binding histone H4 residue K31 and BRD4 to confer cell growth advantage. The main target of this eRNA was considered to be SERINC2 gene and therefore, manipulating this eRNA altered cancer cell metabolite and cancer growth.

Overall, the topic of this study if of broad importance for basic biological understanding of lncRNAs or eRNAs, and has therapeutic relevance to human hematologic malignancies. The inhibition of two MLL over-expressing ELA1/2 eRNAs showed strong phenotypes of cancer inhibition both in vitro culture and in animal models, which is significant for potential cancer therapy. The methods used are overall appropriate for this study (but some controls are missing). Main problems are that: it is not clear if the two eRNAs only or mainly act via the SERINC2 gene via the cis regulation claimed by the authors. The mechanism that eRNAs act via binding histone H4, if proven correct, is potentially novel. However, many evidences are not strong enough to support the mechanistic claims. The paper also suffers from poor presentation, or details are lacking in some areas that prevented clear interpretation. The authors also need to more precisely use their terminologies, and need to check the gramma throughout the text.

Major concerns:

1. The authors need to first describe better where the enhancers and eRNAs are located relative to the target gene. It is a routine to show genome browser track of RNA-Seq together with some histone or transcription factor ChIP-Seq (H3K27ac, H3K4me1, BRD4, pol2, or HOXA proteins etc.) to visually show their genomic relationship and epigenomic environment (these could be either data generated by the authors or from public domain). This is an important basis to judge the potential relationship between the eRNAs and the claimed cis target gene or other genes in the neighborhood. Making it more confusing, Fig.5A (or fig.6? the label in the lower corner of Fig.5 writes fig.6) showed some very vague and confusing genomic locations of some enhancers and SEELA1, why are the enhancers closer to the gene named far enhancer? And how far is far? Why only SEELA1 is shown in Fig.5A? Something like Fig.1D right side, and Fig.5A, but in a large scale and containing more clearer labeling of distance and epigenetic features have to be presented.

2. The naming and description of the top lnc-eRNAs are confusing. In Fig.1, it considered SERINC2-1 ELA1/2 as two RNA transcripts. But in Fig.1D, there are multiple isoforms. The 6,806bp isoform of ELA2 (v1) started from the same 5' region as the ELA1 (v1,2,3), then they essentially share the same start site? Then why they were named ELA2 or ELA1? This relates to some of the other data for example in Fig. 1C,F and Fig.S1H, where did the ChIP-qPCR primers target to (where is the so-called "the promoter of SERINC2-ELA1/2" in Line 132?). The lack of clear description prevents this Reviewer from fully understanding and judging many of the data. Also, Fig.S1G showed that the qPCR primers used in most of the paper examined the sum of all the isoforms of ELA1 or ELA2.

Where are the shRNA and siRNA targeting these two RNAs? what isoform is being knocked down in Fig.4,5,6 for phenotype studies? In Fig.S2C, the knockdown by siRNA to each ELA1 or ELA2 reduced the other RNA as well. How do the authors think of these? siELA1 reduced ELA2 even better than siELA2 in MV4-11; siELA2 reduced ELA1 better than siELA1 in RS4:11 cells?

3. The finding about ELA1/2 RNAs bind histone directly is potentially interesting and novel. But many controls are missing. For example, in RIP or in vitro RNA-protein binding assays (Fig.2, Fig.S2 and S3), the authors should include some other eRNAs or lncRNAs, and the antisense sequences of the ELA1/2 RNAs to examine if the binding found by the experiments is specific to these two eRNAs (ELA1/2) or just simply a low-affinity general binding between histones and RNAs. The authors may not need to do all experiments again, but at least some key RIP and in vitro IP need to include these controls.

4. The authors interpret their results as that H4K31 is the key binding residue of histone H4 with the eRNAs. However, there are several results not fully supporting this. In Fig. 3B, the truncated 1-50aa of H4 binds ELA1-V1 with only 10% of input, ~1/4 of that of full length H4, suggesting that the C terminus of the H4 is very important for binding in vivo (in cells). The in vitro data in Fig.3C,D,E supported that K31 is likely important, but is not the soly important region. The authors also need to confirm the lack of binding between K31A and ELA-V1 using in vivo RIP (like in Fig.3B). Can the over-expression of K31A mutant compete with wildtype H4 and therefore reduce ELA1/2 or SERINC2 gene expression?

5. There is a conceptual paradox of eRNA as cause or consequence of BRD4 binding to enhancers. This paper first showed that BET inhibitors reduced the two eRNAs. But the eRNA knockdown also reduced BRD4 binding and Pol2 loading. How to conceptually think of the temporal order of transcription machinery recruitment and eRNA transcription and functions?

6. The paper concluded that ELA1/2 act in cis. This may be true for the SERINC2 gene regulation. But the cancer inhibitory role of ELA1/2 cannot be fully attributed to SERINC2. This is because: 1) the authors did not check genes other than SERINC2; 2) it is not clear if SERINC2 reduction is the main cause of cancer phenotypes in siELA1/2 cells; can cell growth inhibition be rescued in siELA1/2 cells if the authors overexpress SERINC2 protein?

7. The part about HOXA9/10 is not connected well to the other parts, and is hard to interpret. If the ELA1/2 enhancers were not bound by HOXA9/10, but it only binds far-enhancer-1 in between the eRNAs and SERINC2 genes, then how can HOXA9/10 regulate the ELA1/2 eRNAs? How far is far-enhancer1 from ELA1/2?

Others:

a. Line 25, 58 and 59: "Long non-coding enhancer RNAs (lnc-eRNAs) are a subset of highly conserved..". lncRNAs or lnc-eRNAs are not conserved evolutionarily as an entire group. The authors need to cite literature for this point.

b. Line 33, the authors should write more clearly what subtype of leukemia this paper is focusing on.

c. Line55: none of ref3,4 talked about eRNAs. There are better literature to cite. Line 71, ref16 and 19 are identical.

d. The name SERINC2-ELA1/2 is too long, especially in those with siRNA knockdown. I suggest the authors use this only once in the beginning, then use ELA1 or 2 in later part of the paper.

e. Line130 - why the authors did not knockout the enhancer in MLL cell lines and conduct reporter assays?

f. Line 196, this should not be H4 mutant, it is more appropriate to call it H4 fragment.

g. Fig.1e and Line 768, what are MLL versus MLL-wt? are these MLL patient tumor samples versus adjacent non-tumor tissues? Or versus control groups of health donors? Are these RT-qPCR? What is the Y axis? Are their levels higher than the cis-target gene SERINC2 mRNA (Fig.2A)? How comparable are the levels of ELA1/2 in cancer samples versus the cell lines used for most functional studies?

h. Fig.S1F, are the DeltaCt on the Y axis indicating that of the eRNA minus that of GAPDH?

i. Fig.S2D, the ELA2 level is higher than ELA1? This contradicts those in Fig.S1F.

j. Fig.S3E, the binding between ELA2 with BRD4 is quite weak. If the authors calculate the ratio of IPed band versus the input band, it is perhaps not much higher for BRD4 than for Tubulin.

k. Fig.3B, are the truncated H3 or H4 fragments expressed at comparable levels by Western?

l. Line 546, how long are the two eRNAs used for TRSA pull down assay? Full length?

m. Fig.6E, the knockdown efficiency for HOXA9 in Western is very weak, making the qPCR results not convincing (are there any genes located near ELA1/2 not changed in these samples?).

n. Line 299, HOXA9/10 are transcription factors that may initiate enhancer activation, but it is not correct to say they are "writer".

o. In many places, the gramma is not correct: e.g. line67, "The cis-acting is a general regulation mechanism" should be "Cis regulation is a general mechanism". In some others, the writing uses wrong wording, e.g. line 120, "were transcribed through BRD4 reading" - RNAs did not transcribe through brd4 reading; BRD4 binding helps to activate eRNA transcription. There are a lot of cases of such and the senior authors need to proof-read those.

**Reviewer 2**

**Are you able to assess all statistics in the manuscript, including the appropriateness of statistical tests used?** Yes, and I have assessed the statistics in my report.

**Comments to author:**

In this manuscript the authors studied a particular group of lncRNAs. They took previously identified lncRNAs and overlapped these with H3K27ac ChIP-seq tracks, to identify so-called lnc-eRNAs (enhancers lncRNAs). However, H3K27ac is also present at promoters, as is BRD4 , so the authors should use additional confirmation that these are enhancer only lncRNAs, for example enrichment of H3K4me1, absence of H3K4me3, p300 binding, etc. Based on the presented results iBET responding lncRNAs would be a better name to classify these lncRNA's. Actually, the authors state that the promoters of these lncRNAs do have some H3K4me3, suggesting that lnc-eRNA might be normal lncRNAs. A schematic diagram indicating the positions of the enhancer and promoter (and the far-enhancers analyzed later) on the lncRNA would help, also to estimate whether the resolution of the ChIPs is sufficient to classify these regions as independent. A positive control for the H3K4me3 ChIP would also help here to interpret the results, as well as a factor that goes up after iBet treatment in 1f; all enrichments go down and you want to exclude this is not due to a ChIP experiment going less efficient in iBet treated cells. Also, what is meant with the sentence: "We detected SERINC2 expression via knocking out predict enhancer DNA locus in the SERINC2-ELA1/2 non-expressed 293T cell line"? If SERINC2-ELA1/2 is not expressed in 293T cells, why would you knock it out?

Next the authors showed that eRNAs bind histones. They showed when isolating chromatin fractions enrichment for eRNAs, and via pull downs that eRNAs interacted with histones. By performing deletion/point mutation analysis of Histone H4 they identified K31 as a crucial positively charged aa that interacted with the negative charged eRNA. This is a very interesting finding and reports a direct RNA-histone interaction. The importance of just one lysine aminoacid in this interaction is striking, especially because H4, but also the other histones contain many lysines. Can the authors explain how the specificity for this particular lysine could occur?

They next showed that BRD4 can interact with eRNAs and propose eRNAs stabilize BRD4 via eRNA and binding to K-ac simultaneously. They show this at an enhancer site of the lncRNA, but it would be informative to also include the promoter of SERINC2, as it would suggest a mechanism in which the eRNA enhances transcriptional initiation from the nearby promoter.

Also, functional assessment was performed, which confirmed that perturbation of SERINC2 and the eRNAs resulted in comparable phenotypes, an important finding. Finally, the importance for HOXA9 in this regulatory mechanism is explained. This latter part has similar issues as described above: the trend/response is similar in all panels. It would be beneficial for the interpretation if additional controls are included that show the opposite response.

Please correct typos throughout the manuscript, such as in Fig 1b: non changded lnc-eRNAs

**Authors Response**

**Point-by-point responses to the reviewers’ comments:**

**Point-by-point response to reviewers’ comments and questions**

We appreciate all the comments and criticisms and have revised the manuscript following the suggestions. According to the comment from the reviewer 1 ( comment d) that “the name SERINC2-ELA1/2 is too long”, we have shortened the name of these two lnc-eRNAs as SEELA1/2 (SERINC2-ELA1/2) and used the name throughout the revised manuscript and the response letter. Thank you.

Reviewer #1: In the manuscript by Fang et al., entitled:" Cis-acting lnc-eRNA SERINC2-1 ELA1/2 directly binds H4 to promote histone recognition and leukemia progression", the authors aim to identify and functionally characterize lncRNAs generated from enhancers (i.e. lnc-eRNAs) that are deregulated in hematologic malignancies, and to provide potential new therapeutic targets. The authors used previously published microarray data to identify deregulated lncRNAs in cancer that overlap enhancer H3K27ac mark, which resulted in tens of putative lnc-eRNAs. They used cell lines to validated some of these lnc-eRNAs and identified two candidates "SERINC2-1 ELA1/2" due to their sensitivity to BETi / BRD4 inhibition, and their overexpression in MLL mutant cancers, and found that they potentially act via binding histone H4 residue K31 and BRD4 to confer cell growth advantage. The main target of this eRNA was considered to be SERINC2 gene and therefore, manipulating this eRNA altered cancer cell metabolite and cancer growth.

Overall, the topic of this study if of broad importance for basic biological understanding of lncRNAs or eRNAs, and has therapeutic relevance to human hematologic malignancies. The inhibition of two MLL over-expressing ELA1/2 eRNAs shown strong phenotypes of cancer inhibition both in vitro culture and in animal models, which is significant for potential cancer therapy. The methods used are overall appropriate for this study (but some controls are missing). Main problems are that: it is not clear if the two eRNAs only or mainly act via the SERINC2 gene via the cis regulation claimed by the authors. The mechanism that eRNAs act via binding histone H4, if proven correct, is potentially novel. However, many evidences are not strong enough to support the mechanistic claims. The paper also suffers from poor presentation, or details are lacking in some areas that prevented clear interpretation. The authors also need to more precisely use their terminologies, and need to check the gramma throughout the text.

**Reply:** We thank the reviewer’s comment on the broad importance and interest of our study. We also agree with the concerns raised and have performed additional experiments to address the criticisms and questions. Please see our point-by-point answer and response below.

Major concerns:

**1**. The authors need to first describe better where the enhancers and eRNAs are located relative to the target gene. It is a routine to show genome browser track of RNA-Seq together with some histone or transcription factor ChIP-Seq (H3K27ac, H3K4me1, BRD4, pol2, or HOXA proteins etc.) to visually show their genomic relationship and epigenomic environment (these could be either data generated by the authors or from public domain). This is an important basis to judge the potential relationship between the eRNAs and the claimed cis target gene or other genes in the neighborhood. Making it more confusing, Fig.5A (or fig.6? the label in the lower corner of Fig.5 writes fig.6) showed some very vague and confusing genomic locations of some enhancers and SEELA1, why are the enhancers closer to the gene named far enhancer? And how far is far? Why only SEELA1 is shown in Fig.5A? Something like Fig.1D right side, and Fig.5A, but in a large scale and containing more clearer labeling of distance and epigenetic features have to be presented.

**Reply:** We appreciate the comment and have reorganized the data to show the epigenomic environment of SERINC2-ELA1/2 and the genomic relationship of SERINC2-ELA1/2 and its neighboring genes. According to your **comment d** that “the name SERINC2-ELA1/2 is too long”, we have shortened the name of these two lnc-eRNAs as SEELA1/2 (SERINC2-ELA1/2) and used the name in this letter and the revised manuscript. SEELA1/2 is located at chromosome 1p35.2, within the predicted enhancer of *SERINC2* gene, which located at 71.6kb upstream of the transcription start site of SEELA1. ChIP -seq data showed that the gene locus of SEELA1/2 is enriched in H3K27ac, BRD4 and Pol II, consistent with the notion that SEELA1/2 is transcribed from active enhancer. Due to the lack of RNA-seq data of SEELA1/2, the transcript isoforms of SEELA1/2 were presented according to 5’ and 3’ RACE. **The result is shown below and has also been added into the revised manuscript (revised Figure 1D, page 6, line 133-134, 143-146, marked in blue).**

We found that two of the other genes (*FABP3* and *TINAGL1*) are also located near the SEELA1/2 locus. However, silencing SEELA1/2 has little effect on *FABP3* and *TINAGL1* expression. Thus, we mainly focused on the mechanism underlying the regulation of *SERINC2* by SEELA1/2 in this study. **These new data have been provided below and in revised figure S3D and main text (page 7, line 166-169, marked in blue).** Thank you.

**The revised Figure 1D.** The epigenetic environment of SEELA1/2 and its genomic relationship with SERINC2. SEELA1/2 is located at 71.6kb downstream of SREINC2 and its gene locus is marked by H3K27ac, BRD4 and POL II within two enhancer regions (showed in schematic diagrams (grey)). The ChIP-qPCR primers were designed at the gene body of SEELA1/2 (black).

**The revised Figure S3D.** The genomic loci of *FABP3* and *TINAGL1*. Expression levels of TINAGL1 and FABP3 in MV4-11 and RS4;11 cells transfected with si-NC or si-SEELA1/2, respectively. Error bars reflect ±SEM (ns: no significant) in three independent experiments.

Thanks for the reviewer to point it out that the original fig.5 was mislabeled as fig 6. We have corrected this mistake and apologize for the confusion it caused. In the original version, far enhancer1/2/3 is located at the region 20.5kb, 38.2kb and 59.3kb upstream of SEELA1, respectively. We named them as far enhancers in order to distinguish them from the enhancer of *SERINC2*, where SEELA1/2 is located. We agree with the reviewer’s suggestion and have renamed far enhancer as ELA-enhancer1/2/3 for a clearer interpretation. Also, the epigenetic feature, H3K27ac, is provided in the schematic illustration. **The result has also been added into the revised manuscript (page 13, line 313-316, and page 34-35, 930-936, marked in blue, and revised Figure 5A shown below)**. Thank you for the comment, we appreciate it.

**The revised Figure 5A.** A schematic diagram shows the genomic location and the H3K27ac enrichments of ELA-enahcner 1/2/3 (located at the region 20.5kb, 38.2kb and 59.3kb upstream of SEELA1, respectively). ChIP-qPCR assays of HOXA9 (middle) and HOXA10 (bottom) showed that HOXA9/HOXA10 bound to ELA-enhancer 1 instead of ELA-enhancer 2/3; Error bars reflect ±SEM (*, P<0.05; ***, p<0.001, ns: no significant) in three independent experiments. An IgG antibody was used as negative controls in the ChIP assay.

**2.** The naming and description of the top lnc-eRNAs are confusing. In Fig.1, it considered SERINC2-1 ELA1/2 as two RNA transcripts. But in Fig.1D, there are multiple isoforms. The 6,806bp isoform of ELA2 (v1) started from the same 5' region as the ELA1 (v1,2,3), then they essentially share the same start site? Then why they were named ELA2 or ELA1? This relates to some of the other data for example in Fig. 1C, F and Fig.S1H, where did the ChIP-qPCR primers target to (where is the so-called "the promoter of SERINC2-ELA1/2" in Line 132?). The lack of clear description prevents this Reviewer from fully understanding and judging many of the data. Also, Fig.S1G showed that the qPCR primers used in most of the paper examined the sum of all the isoforms of ELA1 or ELA2. Where are the shRNA and

siRNA targeting these two RNAs? what isoform is being knocked down in Fig.4,5,6 for phenotype studies? In Fig.S2C, the knockdown by siRNA to each ELA1 or ELA2 reduced the other RNA as well. How do the authors think of these?

siELA1 reduced ELA2 even better than siELA2 in MV4-11; siELA2 reduced ELA1 better than siELA1 in RS4:11 cells?

**Reply:** We understand the concern. In the original screening of putative functional lnc-eRNAs in Figure 1B, SEELA1 and SEELA2 were considered as two RNA transcripts. In further study, we identified that ELA1 and 2 have three and two isoforms, respectively, as shown by 5’ and 3’ RACE **in Figure 1C-D**. In particular, we identified that one transcript (the 6,806nt isoform of SEELA2) has the same 5’ site of SEELA1. However, the remaining sequence (6,318nt) of this isoform is almost the same with the other isoforms of SEELA2 **(as shown below)**. Thus, the 6,806nt isoform was still considered as one variant of SEELA2 (SEELA2-V1). Hope that this explanation is clear enough.

The repeat region of SEELA2-V1&2 (marked in grey).

Regarding to the primers for ChIP-qPCR in Fig. 1C, F and Fig. S1H, the primers were designed to be complementary with SEELA1/2’s gene body, which overlaps with putative enhancer region (the so-called “the promoter of SERINC2-ELA1/2” has been revised as “the gene body of SEELA1/2”). According to the comment, we have added the schematic illustration of ChIP-qPCR primers **to the revised Figure 1D** to make it clear. Thanks. The shRNAs and siRNAs, that target SEELA1/2 variants, were

designed according to their shared regions of SEELA1/2 variants. The qPCR primers could examine all the isoforms. **As shown in the original figure S2E**, all variants of SEELA1/2 were downregulated by siRNA/shRNA for phenotype studies. The schematic illustration was also provided to show the targeting sites of siRNA and shRNA on SEELA1/2 in the **revised Fig. S2F. We have added related description in the revised text (page 6, line 146-149, marked in blue).** Thank you.

For the question why knocking down either SEELA1 or SEELA2 resulted in the decreases expression of the other eRNA as well? This is a good comment indeed. Since SEELA1/2 regulates the occupancy of BRD4, we speculate that they may regulate each other via recruiting BRD4. **As shown in Fig. 3I**, SEELA1 knockdown by shRNA dramatically decreased the BRD4 binding at SEELA1 and SEELA2 gene loci, and vice versa. This is the possible reason that silencing SEELA1 or SEELA2 could reduce the other RNA as well.

**3**. The finding about ELA1/2 RNAs bind histone directly is potentially interesting and novel. But many controls are missing. For example, in RIP or in vitro RNA-protein binding assays (Fig.2, Fig.S2 and S3), the authors should include some other eRNAs or lncRNAs, and the antisense sequences of the ELA1/2 RNAs to examine if the binding found by the experiments is specific to these two eRNAs (ELA1/2) or just simply a low-affinity general binding between histones and RNAs. The authors may not need to do all experiments again, but at least some key RIP and in vitro IP need to include these controls.

**Reply:** We appreciate the comments. Following the suggestion, we have performed additional RIP and *in vitro* RNA-protein binding assays with two highly expressed lncRNA, HOTAIRM1 and ENST00000413525, as negative RNA control. **In the revised Figure S3H and Figure S4K,** the RIP assays of histone component and BRD4 could not enrich HOTAIRM1 or ENST00000413525. Also, **as shown below and in revised Figure S4D**, there was no obvious enrichment of H4-FL when using

the antisense sequence of SEELA-V1 in *in vitro* RNA pull-down assays. **These results showed the specific association between SEELA1/2 and H4/BRD4and have been provided in the revised manuscript (page 8,lines 181-184, and page 9, lines 211-213, marked in blue).**

**The revised Figure S4D.** The *in vitro* RNA pull-down assays of SEELA1-V1., TRSA and SEELA1-V1-antisense were used as negative control.

**4.** The authors interpret their results as that H4K31 is the key binding residue of histone H4 with the eRNAs. However, there are several results not fully supporting this. In Fig. 3B, the truncated 1-50aa of H4 binds ELA1-V1 with only 10% of input, ~1/4 of that of full length H4, suggesting that the C terminus of the H4 is very important for binding in vivo (in cells). The in vitro data in Fig.3C, D, E supported that K31 is likely important, but is not the soly important region. The authors also need to confirm the lack of binding between K31A and ELA-V1 using in vivo RIP (like in Fig.3B). Can the over-expression of K31A mutant compete with wildtype H4 and therefore reduce ELA1/2 or SERINC2 gene expression?

**Reply**: Thanks for the comments. In the RIP assays, cells were transiently transfected with plasmids containing HA-tagged full-length or truncated H4 and the binding discrepancy was found between truncated 1-50aa and full-length H4 in SEELA1/2. This could be the reason that SEELA1/2 is associated with chromatin, and that the truncated H4 might not be as efficient as full-length H4 to incorporate into nucleosome and, hence, bound less SEELA1/2 than full-length H4 did. **According to**

**the comment, we have provided a brief discussion on the discrepancy in the revised manuscript (page 9, line 206-210, marked in blue)**.

To support that K31 is important, according to reviewer’s suggestion, we have performed additional RIP assays using H4 K31A. **As shown below**, mutation of K31 significantly reduced the association between H4 and SEELA1-V1, indicating that K31 is an important region for ELA binding. As suggested, we have also constructed the stable HA-tagged H4-FL or H4K31A expressed MV4-11 cells (Fig. S5F shown the immunoblot detection of the expression of HA-tagged H4-FL or H4K31A). Interestingly, the expression of SEELA1/2 and SERINC2 were reduced in the H4K31A-expressed cells (t**he revised Fig.3i**), indicating that H4K31 is important for SEELA1/2-SERINC2 axis. These new results have been provided in the **revised Fig. S4F, Fig. S5F, Fig. 3i** and in the revised manuscript **(page 9-10,lines 222-224, marked in blue)**.

**The revised Figure S4F.** RIP-qPCR detection of the enrichment of H4-FL and H4-K31 of the SEELA1-V1. Immunoblot detection showed the enrichment of full-length and mutant H4 protein.

The revised Figure S5F and Fig. 3i. Immunoblot detection the expression of HA-tagged H4-FL or H4K31A in MV4-11 cells (left, Figure S5F), A schematic diagram shows stable overexpressed HA-tagged H4-full length or H4K31A into MV4-11 cells. The binding of SEELA1/2 is disrupted in H4K31A mutants; as a result, the missing of the SEELA1/2-induced SERINC2 expression. The expression of SEELA1/2 and SERINC2 (right) were down regulated in H4K31A mutant cells versus that in the H4-full length cells. Error bars reflect ±SEM (***, p<0.001) in three independent experiments.

**5.** There is a conceptual paradox of eRNA as cause or consequence of BRD4 binding to enhancers. This paper first showed that BET inhibitors reduced the two eRNAs. But the eRNA knockdown also reduced BRD4 binding and Pol2 loading. How to conceptually think of the temporal order of transcription machinery recruitment and eRNA transcription and functions?

**Reply**: This is a very interesting point and question, thank you. The biogenesis of eRNA stems from a number of transcriptional co-regulators, which bind to H3K27ac through BD modules, to affect chromatin accessibility and transcriptional activation at active enhancers. However, the affinity and selectivity of BD interactions with acetylated lysines are typically weak [1-3], suggesting that additional mechanisms regulating BD binding at enhancer are required and likely to have important implication for gene regulation. In this study, we showed that two lnc-eRNA SEELA1 and SEELA2 bind to histone H4 and subsequently induce the occupancy of BRD4 at chromatin, which is important for the productions of SEELA1/2 itself and the downstream target gene *SERINC2*. Collectively, the biogenesis of eRNAs could be the

consequence of BRD4 binding to enhancer and can in turn promote BRD4 occupation. We speculate that this BRD4-eRNA loop enables enhanced chromatin engagement and transcription activation. **This information has been added in the revised Discussion (page 16,lines 381-391, marked in blue)**.

**6.** The paper concluded that ELA1/2 act in cis. This may be true for the SERINC2 gene regulation. But the cancer inhibitory role of ELA1/2 cannot be fully attributed to SERINC2. This is because: 1) the authors did not check genes other than SERINC2; 2) it is not clear if SERINC2 reduction is the main cause of cancer phenotypes in siELA1/2 cells; can cell growth inhibition be rescued in siELA1/2 cells if the authors overexpress SERINC2 protein?

**Reply**: Following the suggestion, we have evaluated the effects of SEELA1/2 on the expression of two other nearby genes *(PABP3* and *TINAGL1)*. Knockdown of SEELA1/2 via siRNAs did not affect the expression of *PABP3* and T*INAGL1*, showing that ELA1/2 mainly regulates SERINC2 *in cis*, not other neighboring genes. **The result has been provided in revised figure S3D and main text (page 7, line 166-169, marked in blue).**

In order to validate whether SERINC2 reduction is the main cause of cancer phenotypes in SEELA1/2 deficient cells, as reviewer’s comment, we introduced overexpressed-SERINC2 **(the overexpression efficiency was shown in revised Figure S5B and below)** into si-SEELA1/2 cells and found that the cell cycle arrest effects caused by SEELA1/2 siRNAs were rescued **(as shown in revised Figure 4G and below)**. These results showed that the contribution of SERINC2 in the SEELA1/2-induced cancer phenotypes and SEELA1/2 regulates leukemia progression through modulating SERINC2. We appreciate the comment and the new data has been added into the revised version **(page 12,lines 279-283, marked in blue)**.

**The revised Figure S5B (left) and 4G (right).** qRT-PCR confirmation of overexpression of SERINC2 (left) and flow cytometric analysis of cell cycle in MV4-11 cells transfected with indicated siRNA (right).

**7.** The part about HOXA9/10 is not connected well to the other parts, and is hard to

interpret. If the ELA1/2 enhancers were not bound by HOXA9/10, but it only binds far-enhancer-1 in between the eRNAs and SERINC2 genes, then how can HOXA9/10 regulate the ELA1/2 eRNAs? How far is far-enhancer1 from ELA1/2?

**Reply**: We understand the concern. In the part about HOXA9/10, we aimed to address the question whether and how transcription factors are involved in eRNA biogenesis. HOXA9/10 is considered as one of the most important regulatory transcription factors in MLL leukemia. Previous reports show that HOXA9 has enhancer binding preference and rewires leukemia-specific enhancers. However, few reports have focused on its role in lnc-eRNAs activation. Our results showed that HOXA9/10 mediate the enhancer-related histone modification enrichments at lnc-eRNAs loci, which initiate the enhancer activity before the BRD4 reading. We believe that this finding would help to understand both lnc-eRNA biogenesis and functions of HOXA9/10 in leukemia. We have added a short discussion in the revised main text **(page 15, line 356-361, marked in blue)**

HOXA9 is reported to preferentially bind to the enhancers of target genes and recruit the histone modifiers to reprogram the enhancer activity. Here, we found that at

SEELA1/2 locus, a transcribed enhancer region, HOXA9/10 preferentially binds to the upstream enhancer mark H3K27ac enrichment region of SEELA1/2 (ELA-enhancer 1), rather than SEELA1/2 gene body (the transcribed enhancer itself). And reducing the binding of HOXA9/10 at ELA-enhancer 1 affects the enhancer activity of SEELA1/2 locus. We speculated that HOXA9/10 may affect the enhancer activity of SEELA1/2 through recruiting the enhancer histone modifiers as previously reported [4] at the forming chromatin loop between the two enhancers. Far-enhancer 1 **(renamed as ELA-enhancer 1 in the revised version)** is located 20.5kb upstream of SEELA1. **To make this clear, we have provided a schematic illustration in the revised Fig 5A and page 13, line 313-316, and page 34-35, 930-936, marked in blue).**

Others:

a. Line 25, 58 and 59: "Long non-coding enhancer RNAs (lnc-eRNAs) are a subset of highly conserved". lncRNAs or lnc-eRNAs are not conserved evolutionarily as an entire group. The authors need to cite literature for this point.

**Reply:** We appreciate the comment. Indeed, eRNAs, as an overall group, are of low conservation. According to the previous report [5], lncRNAs are of medium to high conservation, and lnc-eRNAs are expected to share most features with lncRNAs rather than with eRNAs, thus we previously express that “lnc-eRNAs are more conserved than eRNAs”. We agree with reviewer’s comment, and have revised the description as “Long non-coding enhaner RNAs (lnc-eRNAs) are a subset of more stable eRNAs.” **(page 2, line 25-26 and page 3, line 59, marked in blue)**.

b. Line 33, the authors should write more clearly what subtype of leukemia this paper is focusing on.

**Reply**: As suggested, we have clearly described the subtype of leukemia, MLL

leukemia, **in the revised manuscript (page 2, line 32, marked in blue)**.

c. Line55: none of ref3,4 talked about eRNAs. There are better literature to cite. Line 71, ref16 and 19 are identical.

**Reply**: We have provided two new relevant references as suggested **(as shown in the revised reference 3 and 4)** and replaced the ref 19. Thank you.

d. The name SERINC2-ELA1/2 is too long, especially in those with siRNA knockdown. I suggest the authors use this only once in the beginning, then use ELA1 or 2 in later part of the paper.

**Reply**: We agree with the suggestion that “the name SERINC2-ELA1/2 is too long”. For the reason that the name of ELA1/2 may not be able to distinguish the SERINC2-ELA1/2 from other lnc-eRNA (names as GENE-ELA1/2), we used SEELA1/2 (SERINC2-ELA1/2) to replace the original SERINC2-ELA1/2 throughout the manuscript. Thank you very much.

e. Line130 - why the authors did not knockout the enhancer in MLL cell lines and conduct reporter assays?

**Reply**: Thanks for the comments. When deleting the enhancer in MLL cell line, it is hard to distinguish the effect of DNA sequence or RNA product (SEELA1/2) on *SERINC2* expression. Thus, to investigate the enhancer activity of SEELA1/2 locus, we conducted reporter assays to verify enhancer activity in the SEELA1/2 non-expressed 293T cells to exclude the influence of lnc-eRNAs. As shown in Fig. S1A, knockout the enhancer in 293T cells resulting in significantly reducing the expression of *SERINC2,* indicating that this DNA sequence is the enhancer of SERINC2. **A clearer description was added in the revised manuscript (page 6, line 1334-138, marked in blue).**

f. Line 196, this should not be H4 mutant, it is more appropriate to call it H4 fragment.

**Reply**: We agree and have renamed it as H4 fragment in the revised manuscript **(page 9,lines 204, marked in blue)**

g. Fig.1e and Line 768, what are MLL versus MLL-wt? are these MLL patient tumor samples versus adjacent non-tumor tissues? Or versus control groups of health donors? Are these RT-qPCR? What is the Y axis? Are their levels higher than the cis-target gene SERINC2 mRNA (Fig.2A)? How comparable are the levels of ELA1/2 in cancer samples versus the cell lines used for most functional studies?

**Reply**: We apologize for vague description in the previous version. MLL-wt patients are those leukemia patients without carrying translocated *MLL* genes. These are RT-qPCR and the Y axis is 2^-ΔCT normalized to GAPDH. We have added the related information in the revised main text and figure legend **(page 30-31, line 828-832, marked in blue)**.

The level of SEELA1/2 is higher than *SERINC2* mRNA in clinical samples **as shown below**. Additionally, SEELA1/2 is expressed at similar level in MLL cell lines (RS4;11 and MV4-11) and patient samples (**as shown below**), suggesting that RS4;11 and MV4-11 are suitable cell line models to study the functions of SEELA1/2.

qRT-PCR of the expression level of SEELA1/2 and SERINC2. Data was analyzed using the
ΔCT method and normalized to GAPDH. qRT-PCR of the expression level of SEELA1/2 in
MLL clinical samples and MV4-11&RS4;11 cell lines. Data was analyzed using the ΔCT
method and normalized to GAPDH.

h. Fig.S1F, are the DeltaCt on the Y axis indicating that of the eRNA minus that of GAPDH?

**Reply**: Thanks for the comments. In Fig. S1F, the DeltaCt on the Y axis indicates that of the eRNA minus that of GAPDH. We have added this description in the revised figure legend **of Fig. S2E in additional file 2.**

i. Fig.S2D, the ELA2 level is higher than ELA1? This contradicts those in Fig.S1F.

**Reply**: We apologize for the confusion. In the original Fig.S2D **(the revised Fig. S2E)**, we measured the sum of SEELA1/2 and the expression level of SEELA2 (V1 and V2) is higher than that of SEELA1 (V1, V2 and V3). But in the original Fig.S1F **(the revised Fig. S3E)**, only SEELA2-V1 was measured and therefore its level is lower than that of SEELA1.

j. Fig.S3E, the binding between ELA2 with BRD4 is quite weak. If the authors calculate the ratio of IPed band versus the input band, it is perhaps not much higher for BRD4 than for Tubulin.

**Reply**: We appreciate the reviewer’s comments. In the original Fig. S3E, although the binding between SEELA2 with BRD4 is not strong, TRSA, the RNA negative control, was not associated with BRD4, showing the specificity of the interaction of SEELA2 (V1+V2) with BRD4. Consistently, Tubulin, the negative protein control, also showed no preference in binding SEELA2 or TRSA. These data clearly showed the binding between SEELA2 with BRD4. Considering the concern raised by the reviewer, we have performed additional experiments to repeat this assay to improve the quality of the data. The new result is shown below and has also been provided in the **revised Fig. S4H**.

**The revised Fig. S4H.** The binding of BRD4 in SEELA2-V1/2 pull-down

k. Fig.3B, are the truncated H3 or H4 fragments expressed at comparable levels by Western?

**Reply**: According to the comments, we have reanalyzed the protein levels as shown below, the truncated H3 and H4 fragment are expressed at almost comparable levels. **This new data has been added into the revised Fig. S4C**.

**The revised Figure S4C.** The expression of truncated H3 and H4 fragment in RIP assays.

l. Line 546, how long are the two eRNAs used for TRSA pull down assay? Full length?

**Reply**: Yes, the full-length of the two eRNAs were used for TRSA pulldown assay. We have indicated it in the revised manuscript **(page 8, line 184-185, marked in blue)**.

h. Fig.6E, the knockdown efficiency for HOXA9 in Western is very weak, making the qPCR results not convincing. are there any genes located near ELA1/2 not changed in these samples?

**Reply**: To address this concern, we quantified the band intensities from the digital image in ImageJ, the intensities of HOXA9 bands in si-NC, si-HOXA9-1 and si-HOXA9-2 samples were 1, 0.612 and 0.647, respectively **(as shown below)**. We further examined the expression of two genes *FABP3* and *TINAGL1*, that are both located near SEELA1/2, as suggested and the results showed that they were not changed after knocking down HOXA9/10 (**the revised Figure S9D**), showing that HOXA9/10 mainly influence the SEELA1/2-SERINC2 axis. **We have rephased the description accordingly in the revised manuscript (page 14, line 326-327, marked in blue).**

The bands intensities of HOXA9 and SERINC2 in si-NC, si-HOXA9-1 and si-HOXA9-2

samples.

**The revised Figure S9D.** Expression level of *TINAGL1* and *FABP3* in MV4-11 cells transfected with si-NC or si-HOXA9/10.

n. Line 299, HOXA9/10 are transcription factors that may initiate enhancer activation, but it is not correct to say they are "writer".

**Reply**: We agree and have rephrased the description as suggested. Thank you!

o. In many places, the gramma is not correct: e.g. line67, "The cis-acting is a general regulation mechanism" should be "Cis regulation is a general mechanism". In some others, the writing uses wrong wording, e.g. line 120, "were transcribed through BRD4 reading" - RNAs did not transcribe through brd4 reading; BRD4 binding helps to activate eRNA transcription. There are a lot of cases of such and the senior authors need to proof-read those.

**Reply**: We apologize for the incorrect gramma and wording. The example sentences have been revised and the manuscript is thoroughly revised to improve the language. Thanks a lot.

Reviewer #2: In this manuscript the authors studied a particular group of lncRNAs. They took previously identified lncRNAs and overlapped these with H3K27ac ChIP-seq tracks, to identify so-called lnc-eRNAs (enhancers lncRNAs).

**1.** However, H3K27ac is also present at promoters, as is BRD4 , so the authors should use additional confirmation that these are enhancer only lncRNAs, for example enrichment of H3K4me1, absence of H3K4me3, p300 binding, etc.

**Reply**: We agree. Following the suggestion, we chose two other key histone marks of enhancer RNAs (high H3K4me1 and low H3K4me3) to further validate the 47 H3K27ac-enriched lnc-eRNAs. **As shown below**, the gene loci of 41 lncRNAs exhibit a high H3K4me1 enrichment and low H3K4me3 enrichment in ChIP-qPCR, consistent with the features of enhancer RNAs, indicating that these 41 lncRNAs are lnc-eRNAs. We have provided the new data in the **revised Table S1 and Fig. S1** and text **(page 5, line 113-118, marked in blue)**.

**The revised Figure S1.** ChIP-qPCR showed that the gene loci of 41 lnc-eRNAs exhibit a high enrichment of H3K4me1 and low enrichment of H3K4me3. GAPDH is an mRNA control. Error bars reflect ±SEM in three independent experiments. An IgG antibody was used as a negative control in the ChIP assays.

2. Based on the presented results iBET responding lncRNAs would be a better name to classify these lncRNA's.

**Reply**: We thank for the reviewer’s suggestion and have used “iBET responding lnc-eRNAs” to classify these lnc-eRNAs in the revised text **(page 6, line 126-128, marked in blue).** In addition, according to the comment from reviewer 1 (comment d) that “the name SERINC2-ELA1/2 is too long”, we have shortened the name of these two lnc-eRNAs as SEELA1/2 (SERINC2-ELA1/2) and used the name in this letter and the revised manuscript hereafter.

3. Actually, the authors state that the promoters of these lncRNAs do have some H3K4me3, suggesting that lnc-eRNA might be normal lncRNAs. A schematic diagram indicating the positions of the enhancer and promoter (and the far-enhancers analyzed later) on the lncRNA would help, also to estimate whether the resolution of the ChIPs is sufficient to classify these regions as independent.

**Reply**: This is a good suggestion. We have added the schematic diagram showing the position of the enhancer and promoter, as well as the far-enhancer (renamed as ELA-enhancer in the revised version) to make it clear. **The result is shown below and has also been added into the revised manuscript (revised Figure 1D, page 6, line 143-146, and revised Figure 5A, page 13, line 313-316, marked in blue).**

**The revised Figure 1D.** The epigenetic environment of SEELA1/2 and its genomic relationship with SERINC2. SEELA1/2 is located at 71.6kb downstream of SREINC2 and its gene locus is marked by H3K27ac, BRD4 and POL II within two enhancer regions (showed in schematic diagrams (grey)). The ChIP-qPCR primers were designed at the gene body of

SEELA1/2	(black)..

**The revised Figure 5A.** A schematic diagram shows the genomic location and the H3K27ac enrichments of ELA-enahcner 1/2/3 (located at the region 20.5kb, 38.2kb and 59.3kb upstream of SEELA1, respectively). ChIP-qPCR assays of HOXA9 (middle) and HOXA10 (bottom) showed that HOXA9/HOXA10 bound to ELA-enhancer 1 instead of ELA-enhancer 2/3; Error bars reflect ±SEM (*, P<0.05; ***, p<0.001, ns: no significant) in three independent experiments. An IgG antibody was used as negative controls in the ChIP assay.

**4.** A positive control for the H3K4me3 ChIP would also help here to interpret the results, as well as a factor that goes up after iBet treatment in 1f; all enrichments go down and you want to exclude this is not due to a ChIP experiment going less efficient in iBet treated cells.

**Reply**: In the revised version, we have performed additional experiments using GAPDH as a positive control for H3K4me3 ChIP-qPCR. As shown in the **revised Fig. S1**, GAPDH showed higher ratio of H3K4me3 compared with H3K4me1 in ChIP-qPCR. We have also performed additional experiments using *B2M* gene as the negative control according the previous report [6], which is insensitive to iBET treatment **(as shown below)**. The new data has been provided **in the revised Fig.S2G and the text (page 7, line 155-156, marked in blue).**

**The revised Figure S2G (right).** ChIP-qPCR detection of the enrichment of BRD4, POL II and MLL-fusion protein at the gene locus of *B2M* in MV4-11 and RS4;11 cells after treatment with i-BET151. Error bars reflect ±SEM in three independent experiments.

5. Also, what is meant with the sentence: "We detected SERINC2 expression via knocking out predict enhancer DNA locus in the SERINC2-ELA1/2 non-expressed 293T cell line"? If SERINC2-ELA1/2 is not expressed in 293T cells, why would you knock it out?

**Reply**: We apologize for the vague description. The RNA products of SEELA1/2 were rarely detected in 293T cells. Thus, we used this cell line to detect the enhancer (DNA) activity of SEELA1/2 locus to exclude the influence of RNA product. As shown in Fig. S1A, the knockout of the DNA locus of SEELA1/2 resulted in significantly reducing the expression of *SERINC2,*indicating that this DNA sequence is the enhancer of *SERINC2*. **We have replaced the description accordingly in the revised manuscript (page 6, line 134-138, marked in blue).**

6. Next the authors showed that eRNAs bind histones. They showed when isolating chromatin fractions enrichment for eRNAs, and via pull downs that eRNAs interacted with histones. By performing deletion/point mutation analysis of Histone H4 they identified K31 as a crucial positively charged aa that interacted with the negative charged eRNA. This is a very interesting finding and reports a direct RNA-histone interaction. The importance of just one lysine aminoacid in this interaction is striking, especially because H4, but also the other histones contain many lysines. Can the authors explain how the specificity for this particular lysine could occur?

**Reply**: This is a good comment indeed. We agree with the reviewer’s comment that the interaction of H4K31 and eRNA is a very striking finding. Before ensuring the K31 lysine of H4 is the crucial amino acid for interacting with eRNAs, we firstly found that the mutant of H4-25-36aa, other than the other regions of H4 or H3 wild type, was important for eRNA binding. In this mutation region (24-36aa), only one lysine (K31) exists, and we have verified that this lysine is crucial for eRNA binding through mutation assay **(as shown in Figure 3E)**. Thus, we exclude other histone lysines and showed that H4K31 is specific for eRNA binding. The specificity for this

particular lysine may be determined by its location. H4K31 lies at the N-terminus of the histone H4α1 helix, and exposes on the outer surface of the nucleosome **(as shown in Figure S4G)** and previous reports [7-8]. The location of H4K31 may provide the space for eRNA-H4 interaction, which could be a possible mechanism for the specific of the H4K31 in eRNA binding. **According to the comment, we have provided a brief discussion in the revised main text (page 10, line 230-234, marked in blue).**

7. They next showed that BRD4 can interact with eRNAs and propose eRNAs stabilize BRD4 via eRNA and binding to K-ac simultaneously. They show this at an enhancer site of the lncRNA, but it would be informative to also include the promoter of SERINC2, as it would suggest a mechanism in which the eRNA enhances transcriptional initiation from the nearby promoter.

**Reply**: We agree and have re-analyzed the BRD4 binding locus in ChIP-seq. However, BRD4 showed less enrichment at the promoter of *SERINC2* **( please see below)**. We speculate that SEELA1/2 affected the expression of *SERINC2* mainly though modulating BRD4 binding at the enhancer locus, which influence the POL II binding at *SERINC2* promoter to regulate the transcriptional initiation **(as shown below).**

The BRD4 enrichments in SERINC2-SEELA1/2 locus.

8. Also, functional assessment was performed, which confirmed that perturbation of SERINC2 and the eRNAs resulted in comparable phenotypes, an important finding. Finally, the importance for HOXA9 in this regulatory mechanism is explained. This latter part has similar issues as described above: the trend/response is similar in all

panels. It would be beneficial for the interpretation if additional controls are included that show the opposite response.

**Reply**: Thanks for the suggestion. We have performed additional experiments using *TINAGL1* and *FABP3* as negative controls in qPCR assays measuring the effect of silencing HOXA9 on gene expression. **As shown in revised Fig. S9D**, knocking down HOXA9 via siRNAs significantly reduced the expression of SEELA1/2, but not that of the control nearby genes *TINAGL1* and *FABP3*. Thank you for the comment. **We have rephased the description accordingly in the revised manuscript (page 14, line 326-327, marked in blue).**

**The revised Figure S9D**. Expression level of *TINAGL1* and *FABP3* in MV4-11 cells
transfected with si-NC or si-HOXA9/10.

9. Please correct typos throughout the manuscript, such as in Fig 1b: non changded lnc-eRNAs

**Reply**: Thanks for the comments. We have carefully gone over the manuscript and figures to correct any typo.

Reference

1. Filippakopoulos P, Picaud S, Mangos M, Keates T, Lambert JP, Barsyte-Lovejoy D, et al. Histone recognition and large-scale structural analysis of the human bromodomain family. CELL. 2012;149:214-31.
2. Umehara T, Nakamura Y, Jang MK, Nakano K, Tanaka A, Ozato K, et al. Structural basis for

acetylated histone H4 recognition by the human BRD2 bromodomain. J BIOL CHEM. 2010;285:7610-8.

3. Moriniere J, Rousseaux S, Steuerwald U, Soler-Lopez M, Curtet S, Vitte AL, et al. Cooperative binding of two acetylation marks on a histone tail by a single bromodomain. NATURE. 2009;461:664-8.
4. Sun Y, Zhou B, Mao F, Xu J, Miao H, Zou Z, et al. HOXA9 Reprograms the Enhancer Landscape to Promote Leukemogenesis. CANCER CELL. 2018;34:643-58.
5. Li W, Notani D, Rosenfeld MG. Enhancers as non-coding RNA transcription units: recent insights and future perspectives. NAT REV GENET. 2016;17:207-23.
6. Dawson MA, Prinjha RK, Dittmann A, Giotopoulos G, Bantscheff M, Chan WI, et al. Inhibition of BET recruitment to chromatin as an effective treatment for MLL-fusion leukaemia. NATURE. 2011;478:529-33.
7. Sindikubwabo F, Ding S, Hussain T, Ortet P, Barakat M, Baumgarten S, et al. Modifications at K31 on the lateral surface of histone H4 contribute to genome structure and expression in apicomplexan parasites. ELIFE. 2017;6.
8. Kim K, Lee B, Kim J, Choi J, Kim JM, Xiong Y, et al. Linker Histone H1.2 cooperates with Cul4A and PAF1 to drive H4K31 ubiquitylation-mediated transactivation. CELL REP 2013; 5:1690-703.

**Second round of review**

**Reviewer 1**

The authors have addressed some of my questions. But they failed to address some other key questions raised in the first round of review. Two main contribution of this paper: 1). The eRNAs here are good new targets for cancer treatment. 2). It has a new mechanism that eRNA binds histone tails. There are still major problems mostly for the contribution 1:

1. The title claimed cis function of these RNAs. The data now is supportive of that these enhancer-produced RNAs can regulate SERINC2 gene expression in cis, but does not regulate two other genes nearby. But the data cannot fully prove that the moderate cis effect on SERINC2 gene expression is the main reason (if even the reason) of tumor regression when the eRNAs were knocked down.

a. To fully prove this, the authors need to add SERINC2 rescue in Fig.4A,B, and Figure 4H,I,J. Technically, western blots are needed to show successful rescue of the SERINC2 expression (not just qPCR in Figure S6). This needs to show the level of expression of SERINC2 protein after KD and how much the rescue can bring the SERINC2 protein level back to normal (or even higher level than normal). The rescue often needs the plasmid-driven SERINC2 to be tagged by a big tag such as GFP to distinguish its expression from the endogenous protein.

2. Similar as above, while this Reviewer appreciates the strong cancer growth inhibition effects after SEELA1 and 2 KD (Figure 4H,I,J and others), a key concern is that SERINC2 is not the main target that underlies the strong cancer regression. Figure 2C and Figure S3C showed very weak (~30-40% of SERINC2 mRNA, and 40-50% protein by SEELA1 shRNA) effect on SERINC2 when SEELA1 or 2 were knocked down. But the cancer-inhibitory effect by SEELA1 or 2 KD was striking, and was as strong as that after SERINC2 mRNA KD itself (Figure 4I). This is a concerning discrepancy. A second discrepancy is that while tumor growth is inhibited similarly by SEELA1/2 shRNA KD or SERINC2 gene KD in Figure 4I, the survival of mouse with SEELA1 or 2 KD in Figure 4J is clearly worse than those with SERINC2 KD. These results strongly suggest SEELA1/2 acts via other genes than SERINC2.

a. The authors should conduct an RNA-seq and see how many genes are altered genome wild when SEELA1/2 are KD, and if there are obvious other key oncogenes affected by the KD that can explain the cancer growth inhibition better. (per comment 3b, one or two siRNA KD is enough, as all these four siRNAs perhaps changed the eRNA clusters similarly). The conclusion can be that SERINC2 is one of the main targets if there are many genes affected. And the rescue as requested in 1a is important to conclude if the cis role on SERINC2 is the major reason for cancer regression.

b. Technically, how much did sh-SERINC2 KD reduce its protein (needs to be added to Figure S3C).

3. The names of these lnc-eRNAs were made shorter, which is a better way or presentation. However, their terminology still needs to be clarified further.

a. The authors now added the genome coordinates of these RNAs, and just a quick check via UCSC or Ensembl database, one can find that the so-called SEELA1 are just LDC1P-201, LDC1P-204 and LDC1P-205 (which are considered a pseudogene); and SEELA2 are likely LINC01226 and related isoforms. The authors need to describe the relationship of these RNAs versus these annotated lncRNAs. If they are just these annotated lncRNAs, the author should discuss these clearly in the first part of the study that they used SEELA to rename these existing annotated lncRNAs.

b. The fact that SEELA2 shared the same transcription start site with SEELA1 (new Figure 1D) made it more likely that all these RNAs are generated by the same enhancer (the enhancer 1). SEELA2_v2 started from nowhere (with no histone mark or anything), it may also start from the same start site. While this is still the authors' choice, I suggest the authors call these RNAs together as SEELA eRNA clusters. Importantly, for each Knockdown in Figure 2C and Figure.S3 (by either siRNAs or shRNAs to SEELA1 or SEELA2), the authors can check each of the five transcripts shown in Figure 1D. If all these eRNAs were changed by any of these siRNAs or shRNAs, then this entire eRNA cluster is transcribed coordinately. The authors should then simplify many of the data - it is not necessary to shown multiple siRNAs to SEELA1 or 2 as the KD is basicly reducing all these RNAs.

c. Third, while the authors included the track of ChIP-Seq in Figure 1D. They should include a large scale (for the entire locus as in Figure S3D, from gene FABP3 to gene TINAGL1). They can show the zoom in view as in Figure 1D, but only do so after showing the overview of large scale. It is important to add the ChIP-Seq of BRD4, K27ac and Pol2 in this entire large scale locus and let the readers know how strong is the binding of these factors at this enhancer region as compared to the genes.

d. In the revision, the authors did not try to show an RNA-seq track for SEELA1/2, which is not acceptable (a quick check in SRA or GEO can find many RNA-seq in MV4-11 or RS4-11 cells).

Others:

There are still many claims in the paper that have no cited reference or wrong reference. Many imprecise wording, mistakes and errors remain in many places that I cannot list them all. These should be carefully checked by the last author before it should be submitted to any journal. For example, Reference 1 and 2 are not good choices for Reviewer of enhancers. There are too many good review papers on enhancers, but the authors have chosen to use these two that are more about Enhancer promoter looping and 3D genome. For another example: "Line 59, Compared to other types of eRNAs, lnc-eRNAs are acknowledged to be more stable" was used without reference. It is even unclear if this statement is true. Line 80, BRD4 is "a common enhancer reader" - there is no such thing called "enhancer reader", and BRD4 also has ~20-30% binding at promoters per its ChIP-Seq peak distribution. These problems reflect a lack of precise knowledge of this field. Lines 30 and 103, "Here, by genome-wide screening…" - this is an oversold sentence as there is no screening done in this work. Looking into limited microarray or other datasets should not be considered a screening. These are all important problems raised in the original review, and they were raised for the sake to make this paper more readable, but unfortunately were not given attention.

-	Many grammatical problems remain.

-	Line 118,119, "These lnc-eRNAs were named as GENE-Enhancer-LncRNAs (ELAs) according to their predicted target genes", how was the prediction of target gene conducted?

-	The authors may cite two recent reviewers on eRNAs in gene regulation and in cancer: Vittorio Sartorelli and Shannon M. Lauberth, Nature Stru Mol Biol, 2020; Lee et al., RNA Biol, 2020; DOI:10.1080/15476286.2020.1712895.

-	Enhancer activity is often cell type specific. If SEELA1/2 are cell type specific, why deleting the enhancers affected SERINC2 expression in 293T? The authors explain to this original question that they want to distinguish if the DNA of enhancer or the RNAs are important. However, in 293T that the enhancer DNA itself is important (Figure S2A, without eRNA on it), and I assume HOXA9/10 did not bind this enhancer in 293T, then these data actually argue that the eRNAs from this DNA and HOXA9/10 binding are not important for its regulation of SERINC2. Did transfection of siRNAs to SEELA1/2 in 293T reduce SERINC2? This data is confusing, the authors may remove it, or should do enhancer KO in MV4 cells.

-	Technically, Figure S2A, is this a stable cell line with enhancer KO by Cas9? If so, the authors need to show the sanger sequencing of the deletion junction. If not, if this just a transient transfection, and the authors need to do this in MV4-11 cells. Is Figure S2A showing SERINC2 expression? The authors need to show some other genes in the neighborhood not changed by the Cas9 KO.

-	The amounts of in vitro transcribed eRNAs should be shown for the In vitro pull down in Figure 2H,I and Figure 3C,D,E,G.

-	Figure 3I, some genes nearby should be shown to see if the K31A-mutant-caused change is happening on every genes or only some genes.

-	Line 210, the author discussed that H4 1-50aa may not incorporate to chromatin as well as full length. That is possible. They may still want to add that a potential contribution of C terminus of Histone H4 in binding these eRNAs cannot be fully excluded. (as also pointed out in the original comment that in addition to Figure 3B, the in vitro data in Fig.3E supported that K31 is likely important, but is not the solely important region.)

-	The information about CRISPRa (VPR) in Figure 2D needs to be shown better. The location of gRNAs should be shown in Figure 1D. In addition, the method for the CRISPRa should be clearly included in the material and method section.

-	The title should have Histone H4 instead of H4; by H4 alone it is not clear what it is. (i.e. ... directly binds histone H4 to ...).

**Reviewer 2**

All the main concerns have been addressed and this reviewer does not have additional concerns.

**Authors Response**

**Point-by-point responses to the reviewers’ comments:**

Reviewer #1: The authors have addressed some of my questions. But they failed to address some other key questions raised in the first round of review. Two main contribution of this paper: 1). The eRNAs here are good new targets for cancer treatment. 2). It has a new mechanism that eRNA binds histone tails. There are still major problems mostly for the contribution 1:

**Reply:** Thank you for the further comments to make the statement clear. To address the concern regarding to the contribution 1, in this revision, we have performed following experiments: (1) SERINC2 rescue experiments to investigate whether the rescue could restore the phenotype caused by SEELA eRNA cluster knockdown (please see our reply to your comment 1). (2) RNA-seq to demonstrate whether *cis* role on SERINC2 is the major targeting for SEELA-mediated cancer progression (please see our reply to comment 2). We have also addressed other concerns raised, please see our point-by-point response to your criticism and comment below, thank you. As editor suggested, we are now submitting clean version and a marked version in which the changes have been specified as a supplemental file, highlighting these changes in the text.

**Comment 1.** The title claimed cis function of these RNAs. The data now is supportive of that these enhancer-produced RNAs can regulate SERINC2 gene expression in cis, but does not regulate two other genes nearby. But the data cannot fully prove that the moderate cis effect on SERINC2 gene expression is the main reason (if even the reason) of tumor regression when the eRNAs were knocked down.

a. To fully prove this, the authors need to add SERINC2 rescue in Fig.4A, B, and Figure 4H, I, J. Technically, western blots are needed to show successful rescue of the SERINC2 expression (not just qPCR in Figure S6). This needs to show the level of expression of SERINC2 protein after KD and how much the rescue can bring the SERINC2 protein level back to normal (or even higher level than normal). The rescue often needs the plasmid-driven SERINC2 to be tagged by a big tag such as GFP to distinguish its expression from the endogenous protein.

**Reply:** According to your suggestion, we have performed following additional experiments to address this concern:

(1) We applied the tetracycline-inducible system, which has been widely used for gene overexpression and rescue experiments [1], to induce SERINC2 expression in MV4-11 and RS4;11 cells. We also overexpressed SERINC2 in MV4-11 cells using a lentiviral construct to further confirm the result and conclusion. **As shown below,** silencing the lnc-eRNA SEELA resulted in a significant downregulation of SERINC2 protein level, and 4uM DOX can induce SERINC2 expression to as that of control, indicating the successful rescue of SERINC2 expression in SEELA knockdown cells. We then conducted CCK8 and cell cycle assay to evaluate whether SERINC2 rescue could restore the phenotype caused by SEELA eRNA cluster knockdown. Downregulation of SEELA eRNA cluster by si-SEELA2-1 dramatically reduced cell proliferation and induced the cell cycle arrest, which is consistent with our previous results. Notably, the SERINC2 overexpression induced by Dox could rescue the inhibition of cell proliferation and cell cycle arrest in SEELA silenced cells. Similar phenomena were also observed when SERINC2 was overexpressed by a lentiviral construct in MV4-11 cells. Overexpressing SERINC2 could rescue the inhibition of cell proliferation and cell cycle arrest in SEELA silenced cells. **These new data have been added into the revised Figure 4e, f, S6D and main text (page 12, line 288-293, marked in blue).**

**The revised Figure 4e, f.** Schematic outline of SERINC2 rescue strategy (e left upper) in MV4-11 (e) and RS4;11 (f) cells. Western blot showed the protein level of SERINC2 in indicated cells. CCK8 and cell cycle assay showed the cell proliferation and cell cycle arrest of indicated treatment. The error bars indicate the ±SEM values (***, p<0.001) in three independent experiments.

**The revised Figure S6D.** Western blot showed the protein level of SERINC2 in siRNAs transfected in lentiviral vector or SERINC2 expressed MV4-11 cells. CCK8 and cell cycle assay showed the cell proliferation and cell cycle arrest effect. The error bars indicate the ±SEM values (***, p<0.001) in three independent experiments.

(2) As suggested, we have also constructed an eGFP-tagged SERINC2 overexpression vector and transfected it into SEELA silenced cells or control cells. **As shown below**, western blot confirmed the successful expression of eGFP-tagged SERINC2 (100kda band, distinguished from the endogenous protein). Transfection of eGFP-tagged SERINC2 vector could indeed rescue the cell cycle arrest caused in SEELA silenced cells. **These new results have been provided in the Revised Fig S6E and main text (page 12, line 293-296, marked in blue).**

**The revised Figure S6E.** Schematic outline of SERINC2 rescue strategy with eGFP tag (left upper). Western blot of SERINC2 (right) and cell cycle assay (left bottom) of transfected cells. The error bars indicate the ±SEM values (***, p<0.001) in three independent experiments.

**Comment 2.** Similar as above, while this Reviewer appreciates the strong cancer growth inhibition effects after SEELA1 and 2 KD (Figure 4H, I, J and others), a key concern is that SERINC2 is not the main target that underlies the strong cancer regression. Figure 2C and Figure S3C showed very weak (~30-40% of SERINC2 mRNA, and 40-50% protein by SEELA1 shRNA) effect on SERINC2 when SEELA1 or 2 were knocked down. But the cancer-inhibitory effect by SEELA1 or 2 KD was striking, and was as strong as that after SERINC2 mRNA KD itself (Figure 4I). This is a concerning discrepancy. A second discrepancy is that while tumor growth is inhibited similarly by SEELA1/2shRNA KD or SERINC2 gene KD in Figure 4I, the survival of mouse with SEELA1 or 2 KD in Figure 4J is clearly worse than those with SERINC2 KD. These results strongly suggest SEELA1/2 acts via other genes than SERINC2.

**2a.** The authors should conduct an RNA-seq and see how many genes are altered genome wild when SEELA1/2 are KD, and if there are obvious other key oncogenes affected by the KD that can explain the cancer growth inhibition better. (per comment 3b, one or two siRNA KD is enough, as all these four siRNAs perhaps changed the eRNA clusters similarly). The conclusion can be that SERINC2 is one of the main targets if there are many genes affected. And the rescue as requested in 1a is important to conclude if the cis role on SERINC2 is the major reason for cancer regression.

**Reply:** To further address the concern raised by the reviewer, following the suggestion, we have performed RNA-seq in MV4-11 cells transfected with si-NC or si-SEELA2-1. Each sample has three replications. In total, 57 genes were found dysregulated in the si-SEELA samples with fold change>1.5, FDR<0.05 In the si-SEELA samples, SERINC2 was significantly downregulated (Fold change=2.45, FDR=8.64×10^-10^), while the other two nearby genes FABP3 or TINAGL1 did not show obvious variation (**as shown below**), consistent with our previous qPCR results.

We have also done RNA-seq in the MV4-11 cells transfected with si-SERINC2-1 in order to compare the dysregulated genes in si-SERINC2 group with those in si-SEELA group. We found that 49 of 57 dysregulated genes (86.0%) in si-SEELA group were also found to be dysregulated in si-SERINC2 group (42 out of 49 with fold change>1.5, FDR<0.05 and 7 out of 49 with fold change >1.38 and FDR<0.1)**( as shown below),** showing that SEELA and SERINC2 shared most of the same downstream gene sets. Thus, it can be inferred that SEELA regulates these downstream genes mainly through modulating SERINC2. **These new data have been provided in the in the revised figure 4G, S6F, Table S2 and main text (page 12-13, line 297-311, marked in blue).**

We also observed that 8 of the 57 (14.0%) genes were only dysregulated in si-SEELA, including the downregulation of *C3AR1*, *MUC1*, *TMEM156* and the upregulation of *MT1E*, *MT1G*, *TTC21A*, *IMT2A*, and *IL17RE*. We analyzed the loci of these 8 genes, they are located at different chromosome or chromosome arm from SEELA locus, implying that these eight genes might not be regulated by the lnc-eRNA SEELA *in cis*. We agree with the comment that, at this stage, we cannot completely exclude whether the lnc-eRNA SEELA could also play a regulatory role on other genes *in trans*. But based on the rescuer experiments and RNA-sequencing, SERINC2 showed to be an important downstream target of SEELA. **Thus, we have provided a brief discussion on this comment in the Discussion section (page 19, line 448-456, marked in blue).** We appreciate the comment to improve the manuscript.

**The revised Figure 4G and S6F**. Dysregulated genes in si-SEELA2 group and si-SERINC2 group (left, Fig 4g). Heatmap of the relative expression of SERINC2, SEELA, TINAGL1 and FABP3 in RNA-seq of si-NC group or si-SEELA2-1 group (right, Fig S6F).

As for the reviewer’ concern about *in vivo* assay in Figure 4I and J, the reviewer questioned why the proportion of GFP+ cell implantation in bone marrow of si-SEELA and si-SERINC2 group showed almost similar pattern, but the survival curve was not exactly the same. This could be the reason that the GFP+ cells were examined in early stage of cell implantation, and thus the discrepancy was not significant at this stage. Bone marrow microenvironment generally may affect the cell implantation. For the concern why the survival of mouse with SEELA KD in Figure 4J is worse than those with SERINC2 KD, we have taken the suggestion to analyze the protein expression level of SERINC2 in sh-SERINC2 cells (please see our reply to comment 2b), the result showed that 60% of SERINC2 mRNA and 85% protein were lost, which was significant than the effect on SERINC2 in sh-SEELA group, and might be an explanation for longer survival of mouse with SERINC2 KD than those with SEELA1 or 2 KD.

**2b.** Technically, how much did sh-SERINC2 KD reduce its protein (needs to be added to Figure S3C).

**Reply:** We agree and have performed western blots to show SERINC2 protein level. As shown below the protein level of SERINC2 was reduced to 15% in sh-SERINC2 cells. **We have added the data in revised Figure S6G and main text (page 13, line 313-314, marked in blue).** Thank you.

**The revised Fig. S6G.** qPCR and western blot assays detected the mRNA and protein level of sh-NC and sh-SERINC2 cells, respectively. The error bars indicate the ±SD values (***, p<0.001) in three independent experiments.

**Comment 3.** The names of these lnc-eRNAs were made shorter, which is a better way or presentation. However, their terminology still needs to be clarified further.  

**3a.** The authors now added the genome coordinates of these RNAs, and just a quick check via UCSC or Ensembl database, one can find that the so-called SEELA1 are just LDC1P-201, LDC1P-204 and LDC1P-205 (which are considered a pseudogene); and SEELA2 are likely LINC01226 and related isoforms. The authors need to describe the relationship of these RNAs versus these annotated lncRNAs. If they are just these annotated lncRNAs, the author should discuss these clearly in the first part of the study that they used SEELA to rename these existing annotated lncRNAs.

**Reply:** We agree and have rephrased the description to make the terminology accurate and clear as suggested. According to previous lncRNA profiling using the Human LncRNA Array v2. platform [2], SEELA1 and SEELA2 were referred to ENST00000418618 and NR_027085 in GRCh37/hg19 human reference genome, and were also named as LDC1P and LINC01226 in GRCh38/hg38, respectively. Although LDC1P and LINC01226 showed five and nine transcript variants in GRCh38/hg38, respectively; our RACE assays have verified that there are only three and two transcript variants detectable in leukemia cells, respectively. **Following the comment, we have added the related description about the relationship of SEELA1/2 versus these annotated lncRNAs in the first part of the study (page 6, line 134-136, marked in blue).** Thank you for the comment to make the naming clear.

**3b.** The fact that SEELA2 shared the same transcription start site with SEELA1 (new Figure 1D) made it more likely that all these RNAs are generated by the same enhancer (the enhancer 1). SEELA2_v2 started from nowhere (with no histone mark or anything), it may also start from the same start site. While this is still the authors' choice, I suggest the authors call these RNAs together as SEELA eRNA clusters. Importantly, for each Knockdown in Figure 2C and Figure.S3 (by either siRNAs or shRNAs to SEELA1 or SEELA2), the authors can check each of the five transcripts shown in Figure 1D. If all these eRNAs were changed by any of these siRNAs or shRNAs, then this entire eRNA cluster is transcribed coordinately. The authors should then simplify many of the data - it is not necessary to shown multiple siRNAs to SEELA1 or 2 as the KD is basicly reducing all these RNAs.

**Reply:** We agreed and have taken the suggestion to clarify SEELA1 and SEELA2 as SEELA eRNA clusters throughout the manuscript in the revised version. In the SEELA eRNA clusters, SEELA1 has three variants: SEELA1-v1, -v2, and –v3 and SEELA2 includes two: SEELA2-v1 and -v2. In response to your comment, we have re-checked the expression of all the five transcripts by using siRNAs against SEELA1 or SEELA2. The results showed that the expression of all the five transcripts were reduced under the condition indicated in the figure as shown below**. The related result and description have been added in the revised Figure. S3D and main text (page 7-8, line 173-176, marked in blue).** Thank you.

**The revised Figure S3D.** The relative expression level of SEELA1 and SEELA2 isoforms. The error bars indicate the ±SD values (*, P<0.05; **, p<0.01; ***, p<0.001) in three independent experiments.

**3c**. Third, while the authors included the track of ChIP-Seq in Figure 1D. They should include a large scale (for the entire locus as in Figure S3D, from gene FABP3 to gene TINAGL1). They can show the zoom in view as in Figure 1D, but only do so after showing the overview of large scale. It is important to add the ChIP-Seq of BRD4, K27ac and Pol2 in this entire large-scale locus and let the readers know how strong is the binding of these factors at this enhancer region as compared to the genes.

**Reply:** Following the comment, we have included the track of ChIP-Seq in a large scale and added chromatin signature (H3K27ac) and BRD4, Pol II occupancy at the entire loci including FABP3, SERINC2, SEELA and TINAGL1. **As shown below**, the enrichments of H3K27 and BRD4 at SEELA eRNA cluster locus are much higher than those at other gene loci, further supporting our finding that SEELA eRNA clusters are transcribed from enhancer region. **We have provided the related results in the revised Figure S2A and manuscript (page 6, line 136-140, marked in blue).**

**The revised Figure S2A**. Distribution of H3K27, BRD4, Pol II peaks across the *FABP3*, *SERINC2*, *SEELA* and *TINAGL1* transcripts.

**3d.** In the revision, the authors did not try to show an RNA-seq track for SEELA1/2, which is not acceptable (a quick check in SRA or GEO can find many RNA-seq in MV4-11 or RS4-11 cells).

**Reply:** We apologize for not showing an RNA-seq track for SEELA1/2. As suggested, we have shown two RNA-seq tracks for SEELA1/2 (SEELA eRNA clusters) in MV4-11 cells **(as shown below)**. The RNA-seq track clearly showed the expression of SEELA eRNA clusters in MV4-11 cells. We also observed RNA-seq peaks at the first intron of SEELA2. One possible reason for this observation might be that SEELA eRNA cluster are generated from the same pre-eRNA and the RNA-seq peaks represented the pre-RNA of SEELA1 and SEELA2, which is consistent with the notion of coordinate transcription of SEELA eRNA cluster. **The RNA-seq tracks have been provided in the in the revised Figure S2E and main text (page 6, line 145-148, marked in blue).**

**The revised Figure S2E.** Genome browser view of RNA-seq data at SEELA locus.

Others:
There are still many claims in the paper that have no cited reference or wrong reference. Many imprecise wording, mistakes and errors remain in many places that I cannot list them all. These should be carefully checked by the last author before it should be submitted to any journal. For example, Reference 1 and 2 are not good choices for Reviewer of enhancers. There are too many good review papers on enhancers, but the authors have chosen to use these two that are more about Enhancer promoter looping and 3D genome. For another example: "Line 59, Compared to other types of eRNAs, lnc-eRNAs are acknowledged to be more stable" was used without reference. It is even unclear if this statement is true. Line 80, BRD4 is "a common enhancer reader" - there is no such thing called "enhancer reader", and BRD4 also has ~20-30% binding at promoters per its ChIP-Seq peak distribution. These problems reflect a lack of precise knowledge of this field. Lines 30 and 103, "Here, by genome-wide screening…" - this is an oversold sentence as there is no screening done in this work. Looking into limited microarray or other datasets should not be considered a screening. These are all important problems raised in the original review, and they were raised for the sake to make this paper more readable, but unfortunately were not given attention.

- Many grammatical problems remain.

**Reply:** We thank the reviewer for pointing these out and have corrected the citation and description as below:

Reference 1 and 2 have been replaced by the References listed below:

1.	Plank JL, Dean A. Enhancer function: mechanistic and genome-wide insights come together. Mol Cell. 2014;55:5-14.

2.	Long HK, Prescott SL, Wysocka J. Ever-Changing Landscapes: Transcriptional Enhancers in Development and Evolution. Cell. 2016;167:1170-87.

The reference [3] has been added after the description “Compared to other types of eRNAs, lnc-eRNAs are acknowledged to be more stable” **(page 3, line 59-60, marked in blue)**.

The original sentence that we have cited in the reference review [3] is:

“and class III eRNAs, which fulfil RNA-dependent functions — this class is probably enriched in relatively abundant or stable eRNAs, especially lnc-eRNAs”

The description of BRD4 has been replaced as “epigenetic reader” **(page 4, line 81, marked in blue)**.

In line 30 and 103, the “genome-wide screening” description has been deleted as suggested.

To avoid any academic or grammatical errors, we have used language editing services listed on the journal website for language polishing; we believe that this editing has improved the language of this manuscript.

- Line 118,119, "These lnc-eRNAs were named as GENE-Enhancer-LncRNAs (ELAs) according to their predicted target genes", how was the prediction of target gene conducted?

**Reply:** Thanks for the comment. The prediction of lnc-eRNAs target genes was based on the previous report [4] and the genomic distance. Briefly, the nearest gene located within 100kb of lnc-eRNA locus is also considered as its target gene. We have provided the description in the revised Results section **(page 5, line 117-121, marked in blue)**.

- The authors may cite two recent reviewers on eRNAs in gene regulation and in cancer: Vittorio Sartorelli and Shannon M. Lauberth, Nature Stru Mol Biol, 2020; Lee et al., RNA Biol, 2020; DOI:10.1080/15476286.2020.1712895.

**Reply:** Yes, we agree and have cited these two articles in the revised manuscript. The review paper (Vittorio Sartorelli and Shannon M. Nature Stru Mol Biol, 2020) was cited as reference 16 in line 71 to explain the *cis* regulation of eRNAs at chromatin level. The second review paper (Lee et al., RNA Biol, 2020) was cited as reference 9 in line 64 to describe the dysregulation of eRNA in cancer.

Thank you.

- Enhancer activity is often cell type specific. If SEELA1/2 are cell type specific, why deleting the enhancers affected SERINC2 expression in 293T? The authors explain to this original question that they want to distinguish if the DNA of enhancer or the RNAs are important. However, in 293T that the enhancer DNA itself is important (Figure S2A, without eRNA on it), and I assume HOXA9/10 did not bind this enhancer in 293T, then these data actually argue that the eRNAs from this DNA and HOXA9/10 binding are not important for its regulation of SERINC2. Did transfection of siRNAs to SEELA1/2 in 293T reduce SERINC2? This data is confusing, the authors may remove it, or should do enhancer KO in MV4 cells.
- Technically, Figure S2A, is this a stable cell line with enhancer KO by Cas9? If so, the authors need to show the sanger sequencing of the deletion junction. If not, if this just a transient transfection, and the authors need to do this in MV4-11 cells. Is Figure S2A showing SERINC2 expression? The authors need to show some other genes in the neighborhood not changed by the Cas9 KO.

**Reply:** We apologize for not clearly explaining the data in previous version. As the influences of genomic loci on nearby genes are regulated by DNA elements themselves or transcripts from them or both, we originally attempted to seek a model, which can exclude effects of transcript (lncRNA) cis-regulation, to determine that SEELA locus is the enhancer of SERINC2. Because leukemic cell lines all expressed a high level of SEELA that may influence the expression of nearby genes via cis-regulation, and would affect the identification of active DNA regulatory element, this is why we did not use leukemic cell lines for this experiment, and instead chose 293T cells which express a very low level of SEELA. We agree with the reviewer’s comment that enhancer activity is cell type specific and 293T might not be an appropriate model, so we have deleted Fig. S2A as suggested. We have applied promoter reporter assay, a well-established experiment for enhancer activity analysis [5-7], to confirm the enhancer activity of SEELA locus **(the original figure S2B)**. Hope that this explanation is clear this time.

- The amounts of in vitro transcribed eRNAs should be shown for the In vitro pull down in Figure 2H, I and Figure 3C, D, E, G.

**Reply:** Thanks for the comment. Fifty pmol RNA of SEELA or tRSA was used in *in vitro* RNA pulldown assay for each sample. **We have provided this information in the revised Methods (page 26, line 637-639, marked in blue).**

- Figure 3I, some genes nearby should be shown to see if the K31A-mutant-caused change is happening on every genes or only some genes.

**Reply:** According to the comment, we have evaluated the expression of two other nearby genes FABP3 and TINAGL1 in H4-K31A and H4-FL cells. **As shown below and in the revised Figure S5G**, the mutation of H4K31 did not alert FABP3 and TINAGL1 expression. **We have also added related description in the revised manuscript (page 11, line 266-267, marked in blue).**

**The revised Fig. S5G.** The relative expression of TINAGL1 and FABP3 in H4-FL and H4-K31A cells. The error bars indicate the ±SD values (ns, no significant difference) in three independent experiments.

- Line 210, the author discussed that H4 1-50aa may not incorporate to chromatin as well as full length. That is possible. They may still want to add that a potential contribution of C terminus of Histone H4 in binding these eRNAs cannot be fully excluded. (as also pointed out in the original comment that in addition to Figure 3B, the in vitro data in Fig.3E supported that K31 is likely important, but is not the solely important region.)

**Reply:** Indeed, although H4K31 showed a strong impact on eRNA interaction, H4K31 mutation could also reduce the association of H4 and SEELA (the original Fig S4F), there may still be possible that the C terminus of H4 might also contribute to eRNA binding. **We have added a brief discussion in the revised version as suggested (page 9, line 219-220, marked in blue).**

- The information about CRISPRa (VPR) in Figure 2D needs to be shown better. The location of gRNAs should be shown in Figure 1D. In addition, the method for the CRISPRa should be clearly included in the material and method section.

**Reply:** Thanks for the comment. We have redone the schematic figure as suggested and the location of sgRNAs has been added **(as shown below and revised Figure 2D**), and included more detailed information about the experiment design in the revised Material and Method section **(page 26, line 627-631, marked in blue)**. Thanks.

**The revised Fig. 2D.** The schematic of CRISPRa experiment and gRNAs location. The relative expression of SEELA1, SEELA2 and SERINC2. The error bars indicate the ±SD values (***, p<0.001) in three independent experiments.

-      The title should have Histone H4 instead of H4; by H4 alone it is not clear what it is.  (i.e. ... directly binds histone H4 to ...).

**Reply:** Thanks for the comment. We agree and have used Histone H4 instead of H4 in the revised title **(page 1, line 1, marked in blue)**.

Reviewer #2: All the main concerns have been addressed and this reviewer does not have additional concerns.

**Reply:** Thank you!

**References**

1.	Baron U, Bujard H. Tet repressor-based system for regulated gene expression in eukaryotic cells: principles and advances. Methods Enzymol. 2000;327:401-21.

2.	Fang K, Han BW, Chen ZH, Lin KY, Zeng CW, Li XJ, et al. A distinct set of long non-coding RNAs in childhood MLL-rearranged acute lymphoblastic leukemia: biology and epigenetic target. Hum Mol Genet. 2014;23:3278-88.

3.	Li W, Notani D, Rosenfeld MG. Enhancers as non-coding RNA transcription units: recent insights and future perspectives. Nat Rev Genet. 2016;17:207-23.

4.	Vucicevic D, Corradin O, Ntini E, Scacheri PC, Orom UA. Long ncRNA expression associates with tissue-specific enhancers. Cell Cycle. 2015;14:253-60.

5.	Yang Y, Su Z, Song X, Liang B, Zeng F, Chang X, et al. Enhancer RNA-driven looping enhances the transcription of the long noncoding RNA DHRS4-AS1, a controller of the DHRS4 gene cluster. Sci Rep. 2016;6:20961.

6.	Alvarez-Dominguez JR, Knoll M, Gromatzky AA, Lodish HF. The Super-Enhancer-Derived alncRNA-EC7/Bloodlinc Potentiates Red Blood Cell Development in trans. Cell Rep. 2017;19:2503-14.

7.	Hsieh CL, Fei T, Chen Y, Li T, Gao Y, Wang X, et al. Enhancer RNAs participate in androgen receptor-driven looping that selectively enhances gene activation. Proc Natl Acad Sci U S A. 2014;111:7319-24.

**Third round of review**

**Reviewer 1**

The authors have addressed my major concerns with the new data. There are still several areas lacking details and/or contain unclear labeling that reduced the readability of this paper. These also prevent full understanding of what has been conducted.

1. The labeling of figure legends remains not clear in many places. For example Figure 4G, the labeling is confusing, and the legend has no details. Why there are two sets of siSERINC2? and why one set uses FC>1.38, the other FC>1.5, and why FDR cutoff is different? The authors should also sue |FC| > 1.5 to be correct, the current labeling indicates that they only showed genes that are unregulated. The authors should consider this paper is to be read by many graduate students who need the details to know what are the figures trying to present.

2. The authors are encouraged to put the sequencing depth of the RNA-Seq as a supplementary table. Are the total RNA-seq or poly-A RNA-Seq? where are the data? are they deposited to GEO or public domain? These should be made clear, and made available for assessment.

3. In describing gene expression changes, the authors should use fold change properly to show up or down regulation. For example, line 301, if this is a down regulation, it should be better presented log2 fold change of -1.3.

**Authors Response**

**Point-by-point responses to the reviewers’ comments:**

**Reviewer #1:** The authors have addressed my major concerns with the new data. There are still several areas lacking details and/or contain unclear labeling that reduced the readability of this paper. These also prevent full understanding of what has been conducted.

**Reply:** Thank you for the furthering comments, please see below our point-by point response, thanks.

**1.** The labeling of figure legends remains not clear in many places. For example Figure 4G, the labeling is confusing, and the legend has no details. Why there are two sets of siSERINC2? and why one set uses FC>1.38, the other FC>1.5, and why FDR cutoff is different? The authors should also sue |FC| > 1.5 to be correct, the current labeling indicates that they only showed genes that are unregulated. The authors should consider this paper is to be read by many graduate students who need the details to know what are the figures trying to present.

**Reply:** We thank the reviewer for pointing this out and have used |FC| instead to make the terminology accurate and clear as suggested.

Regarding to the labeling of Figure 4G, in the original version, we used the cutoff of |FC|>1.38, FDR<0.1 in SERINC2 group to show that this set of genes (49 of 57) were dysregulated in both si-SEELA and si-SERINC2 group. We agree with the reviewer’s concern, and have used the same cutoff (|FC|>1.5, FDR<0.05) for the Figure 4G. The original sentence has been revised as “We found that 42 of 57 dysregulated genes (73.7%) in si-SEELA group were also found to be dysregulated in si-SERINC2 group (|FC|>1.5, FDR<0.05) (Fig. 4g and Additional file 3: Table S2), indicating that SEELA and SERINC2 could affect a large number of same downstream gene sets.”(**page 13, lines 3-4 and lines 9-13 from the top).**

**2.** The authors are encouraged to put the sequencing depth of the RNA-Seq as a supplementary table. Are the total RNA-seq or poly-A RNA-Seq? where are the data? are they deposited to GEO or public domain? These should be made clear, and made available for assessment.

**Reply:** Thanks for the suggestion. The next generation sequencing in this study was performed by poly-A RNA-seq on an illumina noveSeq instrument. The sequencing depth of the RNA-Seq is up to 6 Gites in 150-base paired-end mode. We have added this information into the **Additional file 3: Table S2 and Methods section** (RNA sequencing part, page 27). The raw data with detailed information has been deposited to GEO, and approved number is GSE157040 and has been released in public.

**3.** In describing gene expression changes, the authors should use fold change properly to show up or down regulation. For example, line 301, if this is a down regulation, it should be better presented log2 fold change of -1.3.

**Reply:** We appreciate the comment and have corrected this mistake. Thank you.
